# Complete Inactivation of Sebum-Producing Genes Parallels the Loss of Sebaceous Glands in Cetacea

**DOI:** 10.1093/molbev/msz068

**Published:** 2019-03-20

**Authors:** Mónica Lopes-Marques, André M Machado, Luís Q Alves, Miguel M Fonseca, Susana Barbosa, Mikkel-Holger S Sinding, Marianne Helene Rasmussen, Maria Refsgaard Iversen, Mads Frost Bertelsen, Paula F Campos, Rute da Fonseca, Raquel Ruivo, L Filipe C Castro

**Affiliations:** 1 CIIMAR—Interdisciplinary Centre of Marine and Environmental Research, U. Porto—University of Porto, Porto, Portugal; 2 Department of Biology, Faculty of Sciences, U. Porto—University of Porto, Porto, Portugal; 3 Greenland Institute of Natural Resources, Nuuk, Greenland; 4 The University of Iceland’s Research Center in Húsavík, Húsavík, Iceland; 5 Centre for Zoo and Wild Animal Health, Copenhagen Zoo, Frederiksberg, Denmark; 6 Department of Biology, The Bioinformatics Centre, University of Copenhagen, Copenhagen, Denmark; 7 Center for Macroecology, Evolution, and Climate, Natural History Museum of Denmark, University of Copenhagen, Copenhagen, Denmark

**Keywords:** gene loss, skin lipids, marine mammals, comparative genomics

## Abstract

Genomes are dynamic biological units, with processes of gene duplication and loss triggering evolutionary novelty. The mammalian skin provides a remarkable case study on the occurrence of adaptive morphological innovations. Skin sebaceous glands (SGs), for instance, emerged in the ancestor of mammals serving pivotal roles, such as lubrication, waterproofing, immunity, and thermoregulation, through the secretion of sebum, a complex mixture of various neutral lipids such as triacylglycerol, free fatty acids, wax esters, cholesterol, and squalene. Remarkably, SGs are absent in a few mammalian lineages, including the iconic Cetacea. We investigated the evolution of the key molecular components responsible for skin sebum production: *Dgat2l6*, *Awat1*, *Awat2*, *Elovl3*, *Mogat3*, and *Fabp9*. We show that all analyzed genes have been rendered nonfunctional in Cetacea species (toothed and baleen whales). Transcriptomic analysis, including a novel skin transcriptome from blue whale, supports gene inactivation. The conserved mutational pattern found in most analyzed genes, indicates that pseudogenization events took place prior to the diversification of modern Cetacea lineages. Genome and skin transcriptome analysis of the common hippopotamus highlighted the convergent loss of a subset of sebum-producing genes, notably *Awat1* and *Mogat3*. Partial loss profiles were also detected in non-Cetacea aquatic mammals, such as the Florida manatee, and in terrestrial mammals displaying specialized skin phenotypes such as the African elephant, white rhinoceros and pig. Our findings reveal a unique landscape of “gene vestiges” in the Cetacea sebum-producing compartment, with limited gene loss observed in other mammalian lineages: suggestive of specific adaptations or specializations of skin lipids.

## Introduction

**Fig. 1.**
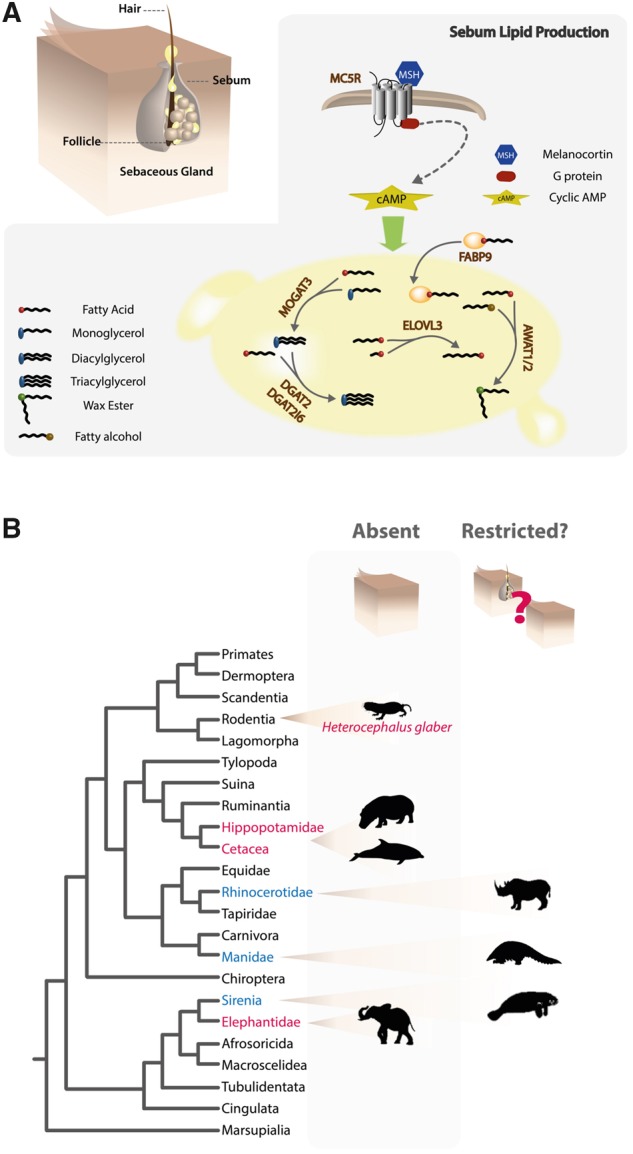
Sebum-producing pathways and phylogenetic distribution of SGs in mammals. (*A*) Schematic depiction of the HF, SG, and sebum lipid production. (*B*) Taxonomic distribution of SG degeneration: lineages suggested to lack SGs are highlighted in red, lineages with conflicting reports on the presence of SGs are highlighted in blue.

Mammalian radiation entailed the successful colonization of multiple and ecologically diverse habitats. This evolutionary path was accompanied by the appearance of novel phenotypic traits in association with genomic changes. A key functional innovation emerging in the ancestors of mammals was the pilosebaceous unit composed of the hair follicle (HF) and the sebaceous gland (SG) (fig. 1*A*) (Lobitz 1957). SGs are composed of sebum-producing cells (sebocytes) that release their content onto the skin surface, playing a key role in sustaining skin homeostasis. The secretion of sebum contributes toward the establishment of the skin barrier: minimizing water loss, supporting thermoregulation, protecting against pathogenic microbiota and UV-induced damage (Lobitz 1957; Smith and Thiboutot 2008; Pappas 2009; Niemann and Horsley 2012). Sebum is a mixture of mostly nonpolar lipids, which are de novo synthesized in the gland. Sebum composition is variable between species, both in lipid concentration and type; yet, six lipid categories are normally found: triacylglycerol (TGA), diacylglycerol (DAG), wax esters, squalene, free fatty acids, cholesterol, and sterol esters (fig. 1*A*) (Nikkari 1974; Stewart and Downing 1991; Pappas 2009). Whereas some of these lipids also occur in other tissues or cell types, squalene and wax esters are unique to SGs (Pappas 2009). In mammals, TGA is synthesized by two distinct pathways: the glycerol 3-phosphate pathway and the monoacylglycerol pathway (Bell and Coleman 1980). In the skin, several enzymes have been suggested to participate in TGA synthesis: notably, monoacylglycerol O-acyltransferases (MOGAT2 and MOGAT3), which produce DAG, and diacylglycerol O-acyltransferases (DGAT2 and DGAT2L6) that further transform DAG into TGA (fig. 1*A*) (Bell and Coleman 1980; Cheng et al. 2003; Holmes 2010; Jiang et al. 2014). Wax ester synthesis, on the other hand, is mediated by acyl-CoA wax alcohol acyltransferases (AWAT1 and AWAT2) (Kawelke and Feussner 2015). Besides esterification enzymes, other proteins have been suggested to participate in skin fatty acid synthesis, elongation, and binding: stearoyl-CoA desaturase 1 (SCD1) (Fluhr et al. 2003), long-chain fatty acid elongase 3 (ELOVL3) (Westerberg et al. 2004; Castro et al. 2016), and fatty acid binding protein 9 (FABP9) (Jiang et al. 2014). Further, knock-out (KO) models have uncovered specific contribution of genes coding for a few of these enzymes into the making of the sebum: such as *Scd1* (the asebia KO) (Fluhr et al. 2003), *Elovl3* (Westerberg et al. 2004), or *Dgat2* (Stone et al. 2004). KO *Elovl3* mice, for instance, yielded a combination of impaired ability to repel water, thermoregulation and an increased rate of transepidermal water loss, underscoring significant changes in the lipid profile of the skin (Westerberg et al. 2004).

The emergence of SGs is a mammalian phenotypic innovation; however, some lineages seem to lack SGs, a feature that was associated with the colonization of specific niches (Springer and Gatesy 2018). As such, Cetacea (whales and dolphins), Hippopotamidae, Elephantidae, and *Heterocephalus glaber* (naked mole rat) were reported to lack SGs; yet, their presence in Manidae (pangolins), Sirenia (manatees and dugongs), and Rhinocerotidae is still disputed, suggesting a complex correlation between SG degeneration and life history traits (fig. 1*B*) (Sokolov 1982; Graham 2005; Rodrigues et al. 2014; Plochocki et al. 2017; Springer and Gatesy 2018). Recently, the absence of SGs was associated to the inactivation of the melanocortin 5 receptor (*MC5R*) in some of these lineages (Springer and Gatesy 2018). *MC5R* is a signal-transducing receptor suggested to modulate lipid production in SGs (fig. 1*A*) (Chen et al. 1997). Disruption of this signaling pathway in mice severely impaired skin water repulsion mechanisms and thermoregulation (Chen et al. 1997). In agreement, complete or partial *MC5R* deletions were identified in Cetacea and Sirenia, whereas inactivating mutations were found in *Ceratotherium simum simum* (white rhinoceros) and *Loxodonta africana* (African elephant) (Springer and Gatesy 2018). Yet, counter-intuitively full *Mc5r* gene sequences were retrieved from the closely related *Elephas maximus* (Asian elephant), as well as from other species also seemingly lacking SGs, such as the naked mole rat or extant Hippopotamidae (*Hippopotamus amphibius* and *Choeropsis liberiensis*) (Springer and Gatesy 2018). This suggests that other evolutionary mechanisms are likely involved in SG degeneration.

Gene inactivation is a well-known driver of evolutionary change (Albalat and Cañestro 2016). The power of this evolutionary mechanism has been described in diverse vertebrate and nonvertebrate lineages (Jeffery 2009; Castro et al. 2014; Emerling and Springer 2014; Albalat and Cañestro 2016; Marti-Solans et al. 2016). For instance, in Cetacea, epidermal alterations, linked to gene loss events, were previously reported as a consequence of adaptation to the aquatic environment: notably hair loss, epidermal thickening, abnormal cornification and desquamation, reducing drag, and minimizing pathogen colonization (Parry 1949; Spearman 1972; Sokolov 1982; Hicks et al. 1985; Liu et al. 2010; Chen et al. 2013; Nery et al. 2014; Oh et al. 2015; Strasser et al. 2015; Plochocki et al. 2017; Sharma et al. 2018). Other examples include the immune system, sense organs, dentition, or digestion (McGowen et al. 2014; Braun et al. 2015; Keane et al. 2015; Huang et al. 2016; Lopes-Marques et al. 2018; Sharma et al. 2018). Here, we scanned the genomes and transcriptomes of ten species to assess the completeness and functionality of genes that are central to lipid production in SGs: *Mogat2*, *Mogat3*, *Dgat2*, *Dgat2l6*, *Awat1*, *Awat2, Scd1*, *Elovl3*, and *Fabp9*. Overall, our results show that skin lipid synthesis is strongly impaired in Cetacea, while presenting intermediary profiles in other Artiodactyls, including *H. amphibius* and *Sus scrofa* (pig), as well as in *Trichechus manatus latirostris* (Florida manatee), *C. s. simum* and *L. africana*.

## Results

To determine the integrity of the nine enzymes central to the lipid synthesis in mammalian SGs, we examined gene annotations and the corresponding genomic regions in a selected set of species, representative of the major mammalian lineages, including nine cetacean species (*Orcinus orca*—orca, *Tursiops truncatus*—common bottlenose dolphin, *Delphinapterus leucas*—beluga whale, *Lipotes vexillifer*—Yangtze river dolphin, *Physeter catodon*—sperm whale, *Balaenoptera acutorostrata*—minke whale, *Balaenoptera bonaerensis*—Antarctic minke whale, *Eschrichtius robustus*—gray whale, *Balaena mysticetus*—bowhead whale) (Zimin et al. 2009; Zhou et al. 2013; Yim et al. 2014; Foote et al. 2015; Park et al. 2015; DeWoody et al. 2017; Jones et al. 2017; Warren et al. 2017; Arnason et al. 2018). To fully investigate the coding status of these genes in Cetacea a three-step strategy was devised: first, gene orthology was assessed to authenticate one-to-one orthologue comparison in combination with synteny analysis; secondly, manual open reading frame (ORF) sequence annotation was performed to validate the coding status; and thirdly, manual annotation was further corroborated using available sequence read archives (SRAs) from at least two independent sequencing projects or two distinct individuals, when possible.

Finally, transcriptomic data from three skin-specific projects were analyzed: one Odontoceti species (*T. truncatus*) available in NCBI (BioProject-PRJNA385781), a novel skin transcriptome from *Balaenoptera musculus* (blue whale—Mysticetus), generated for this work, and, for comparative purposes, a skin-specific transcriptome from *H. amphibius* (common hippopotamus), also generated for this work, representing the closest relative to extant cetaceans and a pivotal species for the timing of gene loss events.

### Skin TGA Synthesis Is Compromised in Cetacea

**Fig. 2.**
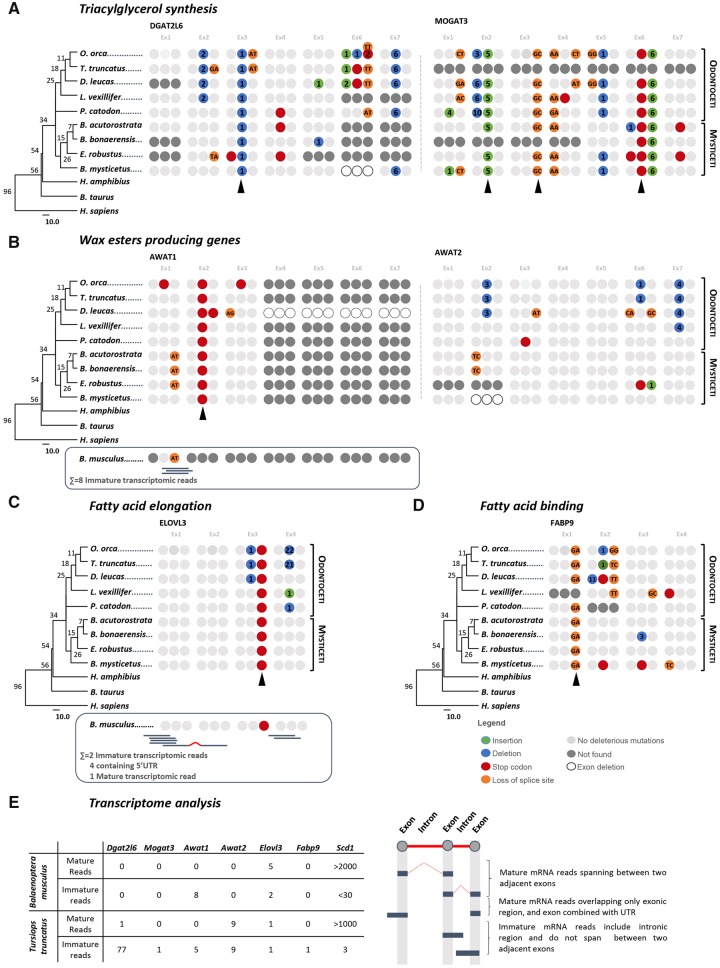
Gene annotation and transcription analysis of sebum-producing genes in Cetacea. Each group of three circles represents a single exon, numbers in the circles indicate number of bases inserted or deleted, whereas nucleotides indicate acceptor or donor splice site mutations. (*A*) *Dgat2l6* and *Mogat3*; (*B*) *Awat1* and *Awat2*; (*C*) *Elovl3*; (*D*) *Fabp9*; and (*E*) transcriptional analysis of the selected genes in *Balaenoptera musculus* and *Tursiops truncatus* skin. Conserved mutations across species are labeled with an arrowhead. Phylogenetic trees were calculated in www.timetree.org ; last accessed March 28, 2019 using species list; values at nodes indicate estimated time of divergence in million years ago.

Initial investigations reveal that *Dgat2* and *Mogat2* (the latter not shown) showed no evidence of inactivation in all analyzed Cetacea. However, a high number of cetacean species displayed “low quality” annotations or no annotation of *Dgat2l6* and *Mogat3* (supplementary material 1: tables S1 and S2, Supplementary Material online). The distribution of both *Dgat2l6* and *Mogat3* in mammals revealed that both genes are found in all major mammalian lineages (supplementary material 1: figs. S1 and S2, Supplementary Material online). *Dgat2l6* belongs to a gene cluster, together with *Awat1* and *Awat2*, which emerged in the ancestor of mammals before the divergence of the marsupial lineage through tandem duplication (supplementary material 1: fig. S3, Supplementary Material online). *Mogat3*, on the other hand is present in amniotes. Comparative synteny analysis showed that both *loci* (*Mogat3* and *Dgat2l6*) are conserved in Cetacea, when compared with the corresponding region in the reference species, thus indicating that the identified genes are orthologous to those found in *Bos taurus* and *Homo sapiens* (supplementary material 1: figs. S3 and S4, Supplementary Material online). Next, we investigated the coding status of *Dgat2l6* and *Mogat3*. We identified several ORF disrupting mutations in both *Dgat2l6* and *Mogat3* in all of the analyzed Cetacea (fig. 2*A*). Regarding *Dgat2l6*, a single mutation, namely a one nucleotide deletion in exon 3, was found to be conserved across all tested species, whereas in *Mogat3* several conserved mutations were identified, such as a five-nucleotide insertion in exon 2, a loss of canonical 3ʹsplice site in exon 3 (GT>GC) and the presence of a stop codon in exon 6 (fig. 2*A*). The identified mutations were next validated by searching at least one ORF disruptive mutation per species in the corresponding SRAs; reads corroborating the identified mutations were systematically found (supplementary material 2, Supplementary Material online). We next searched two skin transcriptomes from *T. truncatus* and *B. musculus* for transcriptional evidence of these genes. Searches were performed using the gene annotations as query as well as a coding gene, *Sdc1*, a fatty acid desaturase which affects sebocyte differentiation (Niemann 2009) and is coding in Cetacea (not shown), as a quality control. *Dgat2l6 and Mogat3* reads were not detected (or residual), in accordance with the presence of inactivating mutations. As expected, searches for *Sdc1* retrieved over 1,000 reads, mostly corresponding to mature RNA transcripts, a good indication of gene transcription (fig. 2*E*).

### Inactivating Mutations in the Wax Esters Producing Genes

We next examined the pathway leading to wax ester production, the second most common fraction of sebum. Using a similar approach, we investigated genes encoding two key enzymes that catalyze the synthesis of wax esters using Acyl-CoA as substrate, *Awat1* and *Awat2* (Holmes 2010; Kawelke and Feussner 2015). Initial analysis showed that these genes are widely distributed in all analyzed mammalian lineages and predicted to be present in the mammalian ancestor (supplementary material 1: fig. S2, Supplementary Material online). Also, as previously noted, comparative synteny analysis confirmed *locus* conservation and orthology of *Dgat2l6* as well as *Awat1* and *Awat2* (supplementary material 1: fig. S3, Supplementary Material online). Thus, we proceeded with the analysis and annotation of the corresponding genomic sequence which revealed numerous mutations in *Awat1* and *Awat2* sequences. In the case of *Awat1*, exons 4–7 were not found in all investigated species, possibly due to poor genome coverage or complete exon deletion (fig. 2*B*). Interestingly, in the case of *D. leucas* this region was found to be fully sequenced presenting no coverage gaps and yet the corresponding exons were not found, constituting a strong indication that these exons are most probably deleted. In addition to the potential exon loss in *Awat1*, a conserved stop codon was identified in exon 2 in all analyzed Cetacea species. This mutation was validated by several SRA projects (supplementary material 2, Supplementary Material online). In contrast, the mutational events identified in *Awat2* did not allow definite validation of pseudogenization in Cetacea. For example, *L. vexillifer* presents only a four-nucleotide deletion in the last exon, leading to a frameshift that culminates in a stop codon four nucleotides before the predicted stop codon. In *O. orca* and *T. truncatus* the deletion of a single nucleotide in exon 6 leads to a frameshift with the appearance of premature stop codon in exon 6. A premature stop codon was also found in exon 6 of *E. robustus*. However, amino- and carboxyl-terminal regions have been proposed to be under less selective pressure and frameshift mutations are highly frequent in these regions with none or little detrimental consequences (Sharma et al. 2016). Also, in *B. acutorostrata* and *B. bonaerensis* the presence of a single mutation in an acceptor splice site (AG>CT) is insufficient to determine the coding status of *Awat2* without further transcriptomic evidence. Regarding *P. catodon*, *Awat2* gene annotation revealed a premature stop codon in exon 3; given that the active site of this enzyme (HPHG) is located in exon 4 (Kawelke and Feussner 2015), it is reasonable to argue that *Awat2* is pseudogenized in this species. Finally, in *B. mysticetus* the deletion of exon 2, which encodes for a transmembrane region, is predicted to significantly alter protein conformation and function. Identified mutations were validated by SRA searches (supplementary material 2, Supplementary Material online). Additionally, using *Awat1* and *Awat2* sequences as query, searches were conducted in *T. truncatus* and *B. musculus* transcriptomes recovering a minute number of reads for *Awat1* and *Awat2* (fig. 2*E*). Yet, the collected reads were analyzed and revealed to be immature, containing intronic sequence remnants. The retrieved reads further disclosed an exon 1 donor splice site mutation in *B. musculus*, similarly to that observed in *B. bonaerensis* and *B. acutorostrata* (not shown).

### Fatty Acid Elongation and Fatty Acid Binding Is Impaired in Cetacea

We next sought to determine whether fatty acid synthesis pathways would be compromised. Not surprisingly, the complement of desaturases (*Fads*) and elongases (*Elovl*), participating in long-chain fatty acid biosynthesis, is apparently intact in the analyzed Cetacea (not shown), with the exception of *Elovl3* (supplementary material 1: fig. S5, Supplementary Material online). The analysis of *Elovl3* in Cetacea revealed that the genomic *locus* of this gene is highly conserved when compared with *B. taurus* and *H. sapiens* (supplementary material 1: fig. S6, Supplementary Material online). Subsequent annotation of the collected genomic sequences revealed a trans-species conserved premature stop codon in exon 3, further validated by SRA searches (fig. 2*C* and supplementary material 2, Supplementary Material online). These results constitute a strong indication that *Elovl3* is inactive in the analyzed species. We next assessed the status of *Fabp9* in cetaceans. *Fabp9* is also known as testis FABP (T-FABP) due to its expression in mouse testis (Oko and Morales 1994; Selvaraj et al. 2010). Yet, transcriptomic and expressed sequence tag analysis revealed that *Fabp9* was highly and exclusively expressed in the skin of Artiodactyls, including cattle and pig (Jiang et al. 2014). Additionally, when consulting the Human Protein Atlas (www.proteinatlas.org; last accessed March 28, 2019), we found that human *Fabp9* was mostly expressed in the internal root of the sheath of the HF, colocalizing with SGs within the pilosebaceous unit (Fagerberg et al. 2014; Yu et al. 2015). Searches in the NCBI database revealed an irregular distribution of *Fabp9* among the analyzed cetaceans, being only annotated in *B. acutorostrata* and *O. orca* (supplementary material 1: table S4 and fig. S7, Supplementary Material online)*.* Synteny analysis of *Fabp9* revealed *locus* conservation between cetaceans with *H. sapiens* and *B. taurus*, with no indication of substantial rearrangements that would substantiate *Fabp9* absence in several species (supplementary material 1: fig. S8, Supplementary Material online). Thus, we collected the genomic regions between neighboring *Pmp2* and *Fabp4* and fully or partially reconstructed *Fabp9* in all analyzed species, revealing a conserved noncanonical donor splice site in exon 1 (GT>GA) (fig. 2*D*); this mutation was further validated by SRA searches (supplementary material 2, Supplementary Material online). Aside from the splice site mutation, premature stop codons were identified in *D. leucas*, *L. vexillifer*, and *B. mysticetus* and frameshift mutations were present in *O. orca* and *T. truncatus*. Interestingly, annotation of the genomic region in *P. catodon* revealed that *Fabp9* exon 1 was placed downstream of exon 4 and inverted. Although we cannot exclude poor genome assembly in this region, the absence of exon 2 in *fabp9* of *P. catodon* may be attributed to this rearrangement.

Next, we investigated *B. musculus* and *T. truncatus* transcriptomes (fig. 2*E*). Regarding *Elovl3*, seven reads were recovered, one mature mRNA read spanning from exon 1 to exon 2, four reads covering the 5′UTR region and exon 1 and 2 immature reads. The second query using *Fabp9* retrieved numerous reads. However, subsequent analysis and assembly revealed that all transcripts corresponded to *Fabp4*, *Fabp5*, *Fabp7*, *Fabp3*, and *Fabp6* gene orthologues (data not shown).

### Partial Convergent Disruption of Sebum Genes in *H. amphibius* and Other Mammals

**Fig. 3.**
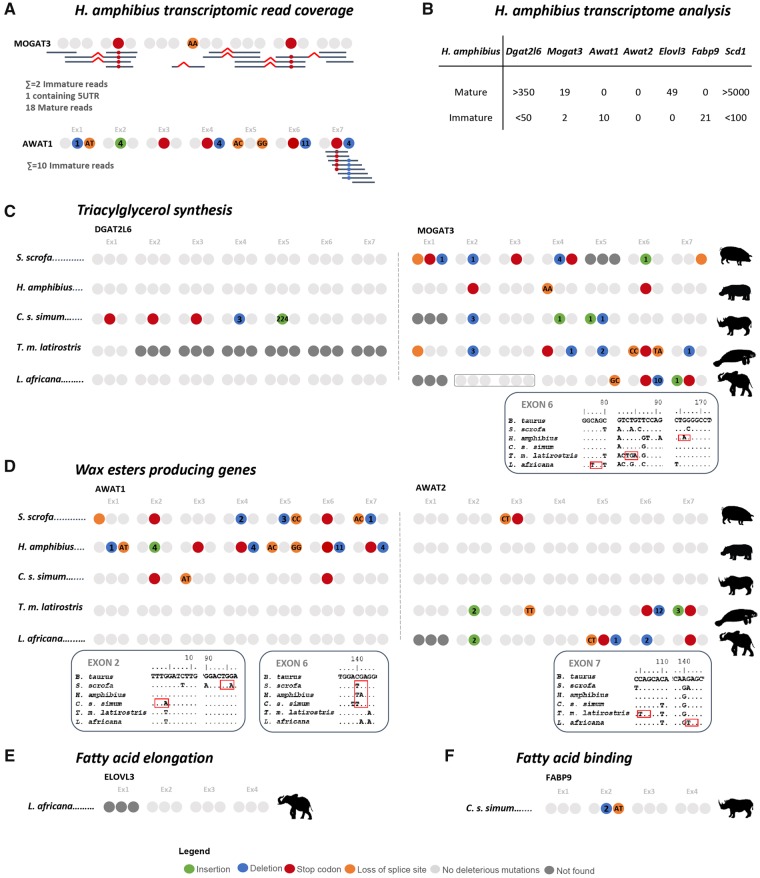
Analysis of coding status of sebum-producing genes in other mammals. (*A*, *B*) Gene annotation and transcriptional analysis in *Hippopotamus amphibius*; (*C*) gene annotation in *Trichechus manatus latirostris*; (*D*) gene annotation in *Sus scrofa*; (*E*) gene annotation in *Loxodonta africana*; and (*F*) gene annotation in *Ceratotherium simum simum.* Each group of three circles represents a single exon, numbers in the circles indicate number of bases inserted or deleted, whereas nucleotides indicate acceptor or donor splice site mutations.

With the exception *Awat2*, we were able to identify single conserved mutational events underscoring the erosion of the skin lipid synthesis-related genes in all analyzed Cetacea, a strong indication that this inactivation most probably took place before the diversification of modern Cetacea. To discern whether these inactivation events occurred in the common ancestor to all Cetacea or predate Cetacea divergence, we further investigated *H. amphibius* that, in addition to being the closest extant relative of cetacean lineage, was reported to have no apparent SGs (Luck and Wright 1964; Irwin and Árnason 1994; Gatesy 1997; McGowen et al. 2009). In *H. amphibius* several deleterious mutations were found in both *Awat1* and *Mogat3*, whereas the remaining genes presented no ORF-disrupting mutations (fig. 3*A*). Regarding *Mogat3*, gene annotation revealed stop codons in exons 2 and 6, the latter located in a distinct codon when compared with Cetacea *Mogat3*. For *Awat1* numerous disrupting mutations, also noncoincident with those observed in Cetacea *Awat1*, were identified. Premature stop codon mutations in exon 2 of *H. amphibius Mogat3* and exon 3 of *Awat1* were further confirmed by SRA (supplementary material 3, Supplementary Material online). We next searched for transcriptional evidence of the apparently functional genes using a de novo produced skin *H. amphibius* transcriptome. Searches for *Scd1* and *Dgat2l6*, genes with no detected deleterious mutation, retrieved a high number of reads, mostly corresponding to maturely spliced mRNA. In contrast, searches for *Elovl3* rescued a lower number of reads, nevertheless all corresponding to mature mRNA (fig. 3*B*). Interestingly, although no deleterious mutations were found in *Fabp9* and *Awat2*, transcriptome searches were unable to recover reads of these genes. This outcome may be explained by two alternative scenarios: (1) *Fabp9* and *Awat2* may be inactivated by mutations in regulatory sites or (2) the preferential site of gene expression of *Awat2* and *Fabp9* occurs in other tissues aside skin. To further evaluate the expression profiles of both genes, we next searched an additional *H. amphibius* RNA-seq project (SRX1164570 available in NCBI) comprising RNA extracted from muscle, skin, heart, liver, spleen, kidney, lung, and neural tissue and still recovered zero reads for both *Fabp9* and *Awat2* (supplementary material 4, Supplementary Material online), suggesting that both *Fabp9* and *Elovl3* expression is probably silenced in *H. amphibius*. Regarding *H. amphibius Mogat3*, although several deleterious mutations were identified, further transcriptome analysis, using a new skin transcriptome, identified some reads. Yet, read assembly and analysis of the corresponding transcripts revealed the presence of two distinct transcript types, one corresponding to *Mogat3* and the second possibly corresponding to a *Mogat3-like* gene (supplementary material 4, Supplementary Material online). Thus, of the identified reads 21 corresponded to *Mogat3*, with 19 maturely spliced reads (fig. 3*B*). Yet, all transcripts encompassing exon 2 and exon 6 exhibited the previously identified stop codons (fig. 3*A* and supplementary material 4, Supplementary Material online). The analysis of the transcripts corresponding to the *Mogat3-like* sequence, revealed no deleterious mutations and probably corresponds to a functional gene (supplementary material 4, Supplementary Material online). A similar scenario was observed when using the additional *H. amphibius* RNA-seq project (supplementary material 4, Supplementary Material online). Finally, for *H. amphibius Awat1*, few immature reads were also found, in our de novo produced skin transcriptome, overlapping the stop codon and deletion identified in exon 7 (fig. 3*A* and *B* and supplementary material 4, Supplementary Material online).

We next expanded our search to other mammalian lineages for which conflicting reports on the presence of SGs have previously been published. For example, the *T. m. latirostris* an exclusive aquatic noncetacean mammal: although Rodrigues *et al.* reported the presence of SGs in the mammary tissue (Rodrigues et al. 2014), others failed to identify the presence of SGs in this species (Sokolov 1982; Graham 2005). Also, for *C. s. simum* Plochocki e*t al.* reported the absence of SGs (Plochocki et al. 2017), whereas other studies reported the presence of small and saccular SGs associated to HFs in *C. s. simum* (Sokolov 1982). We also selected *L. africana* for which no SGs have previously been found (Spearman 1970) and lastly *S. scrofa* in which SGs have previously been identified (Sokolov 1982; Liu et al. 2010). Gene annotation of *Awat1*, *Awat2*, *Dgat2l6*, *Mogat3*, *Elovl*3, and *Fabp9* in these species revealed several episodes of genes inactivation, validated by SRAs (supplementary material 3, Supplementary Material online). Notably, the presence of ORF disruptive mutations in *Mogat3* in all analyzed species (fig. 3*C*), deleterious mutations in *S. scrofa* and *C. s. simum Awat1* (fig. 3*D*) and in *S. scrofa, L. africana* and *T. m. latirostris Awat2* (fig. 3*D*) and in both *Dgat2l6* and *Fabp9* from *C. simum* (fig. 3*C* and *F*)*.* With the exception of a shared stop codon in the exon 6 of *Awat1*, all other mutations are not conserved between these species/lineages (fig. 3*C* and *D*;supplementary material 3, Supplementary Material online).

## Discussion

**Fig. 4.**
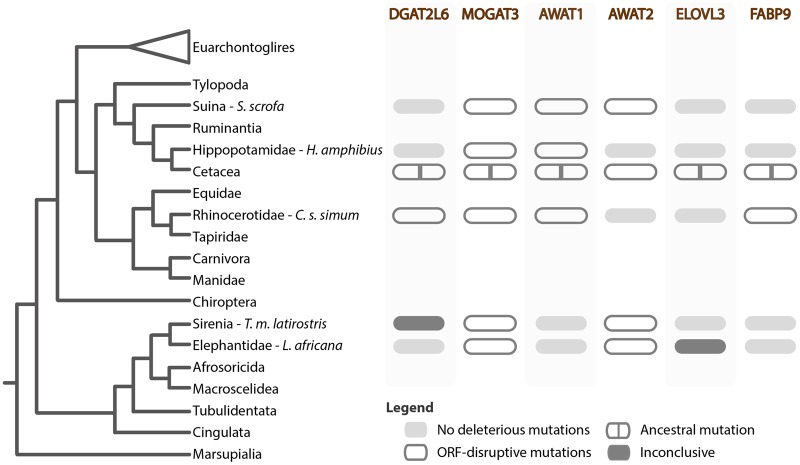
Schematic representation of ancestral and convergent gene loss events of sebum-producing genes in the analyzed species. Critical/representative ORF-disrupting mutations are indicated. Regarding the Cetacea clade, highlighted mutations are conserved between Odontoceti and Mysticeti.

The pilosebaceous unit was a successful innovation in mammalian evolution, playing an important role in epidermal homeostasis: lubricating the skin, creating a hydrophobic and thermoregulating layer, protecting from dehydration and pathogenic microorganisms (Parry 1949; Lobitz 1957; Spearman 1972; Sokolov 1982; Hicks et al. 1985; Niemann and Horsley 2012). Comparative analyses of nine Cetacea genomes demonstrate that the loss of SGs was concomitant with extensive gene inactivation in evolutionary independent molecular compartments responsible for sebum production (fig. 4). The observed transversal gene loss is remarkable but consistent with the absence of SGs in these species (Sokolov 1982; Springer and Gatesy 2018). The analysis and in-depth annotation of genes encoding essential enzymes for the synthesis of TGA (*Mogat3* and *Dgat2l6*), wax esters (*Awat1* and possibly *Awat2*) and fatty acids (*Elovl3* and *Fabp9*) revealed the presence of multiple ORF abolishing mutations in Cetacea (fig. 4). The observed disruption was further confirmed by transcriptome analysis in both toothed (*T. truncatus*) and baleen whales (*B. musculus*), showing little or no transcriptional evidence for the mutated sebum-producing genes. Interestingly, the mutational pattern deduced in *Dgat2l6*, *Mogat3*, *Awat1*, *Elovl3*, and *Fabp9* allowed the identification of conserved founding mutations common to all analyzed Cetacea (fig. 4). The sharing of mutations constitutes a strong indication of common pseudogenization events prior to the diversification of modern Cetacea lineages.

To further investigate the timing of the gene loss events, we analyzed sebum-producing genes from the semiaquatic *H. amphibius*, belonging to the sister taxon of Cetacea (Tsagkogeorga et al. 2015). In *H. amphibius* both *Mogat3* and *Awat1* were clearly pseudogenized, whereas the apparently intact *Fabp9* and *Awat2* yielded no transcriptomic reads in skin samples, as well as in other tissues. No clear shared mutations with Cetacea were identified suggesting independent inactivation of these genes after the divergence of these two lineages (fig. 4). In agreement, previous analysis detected a strong enrichment of positively selected genes in extant cetaceans after the split with Hippopotamidae: suggesting Cetacea-specific molecular adaptations to the fully aquatic environment when compared with the colonization of stagnant and muddy waters by Hippopotamidae (Tsagkogeorga et al. 2015). In this convergent evolution scenario, the loss of a subset of genes in Hippopotamidae could be concomitant with SG degeneration in this lineage. Unlike Cetacea, Hippopotamidae retain an intact *MC5R* gene, previously suggested to parallel SG inactivation (Springer and Gatesy 2018). Also, this alteration of the skin lipid profile could underscore the high transepidermal water loss observed in Hippopotamidae, suggested to serve as cooling mechanism while on land (Luck and Wright 1964).

The extended analysis of sebum-producing genes in other mammals revealed additional episodes of genes loss. Notably, we found that *Mogat3* was transversally inactivated in *T. m. latirostris*, *C. s. simum*, *L. africana*, and *S. scrofa*. Outside Artiodactyls, *Mogat3* was suggested to be commonly expressed in the digestive tract and liver, participating in fat absorption and homeostasis (Cheng et al. 2003; Jiang et al. 2014). Yet, evidence of gene expression specificities is restricted to a few species. The analysis of the *Ovis aries* (sheep) genome and transcriptomes further revealed that, in ruminants, expanded *Mogat3* genes lacked expression in these tissues while being highly expressed in the skin (Jiang et al. 2014). Thus, two scenarios could be put forward regarding the inactivation of *Mogat3*. First, it could reflect convergent skin-specific molecular changes, more specifically degeneration of SGs or changes in sebum lipid composition (Nikkari 1974). In agreement, *T. m. latirostris*, *C. s. simum*, and *L. africana* exhibit derived skin profiles: with absent or restricted SGs and smooth (*T. m. latirostris*) or highly cornified (*C. s. simum* and *L. africana*) thick epidermis (Spearman 1970; Sokolov 1982; Plochocki et al. 2017). Alternatively, *Mogat3* loss could be related to dietary adaptations, especially to herbivory, as in the case of *T. m. latirostris*, *C. s. simum* and *L. africana*. Nonetheless, other inactivating mutations were found, markedly in the wax ester biosynthesis compartment (*Awat1* and/or *Awat2*) in all four species. In addition, *C. s. simum* also displayed erosion in *Dgat2l6* and *Fabp9*. Unlike other analyzed lipid compartments, wax esters are specific to SGs (Pappas 2009). Interestingly, although SGs are clearly present in *S. scrofa*, the wax ester synthesis pathway seems fully impaired. Pigs are notoriously sensitive to environmental temperatures, exacerbated by their lack of sweat glands, similarly to *L. africana* (Weissenböck et al. 2010; Justino et al. 2014). Thus, the apparent lack of wax esters could alter their thermoregulatory abilities and/or the transepidermal water loss to cope with heat, as suggested for Hippopotamidae (Luck and Wright 1964).

In conclusion, our study supports convergent gene loss events in key molecular components responsible for skin sebum production in lineages reported to lack functioning SGs. Complete or partial impairment of the analyzed gene repertoire, along with the lineage-specific inactivation of other SG-related genes such as *MC5R*, provide a unique mosaic distribution pattern of gene loss events which emphasizes the parallelism between SG degeneration and the adaptation to specific ecological niches.

## Materials and Methods

### Sequence Collection and Phylogenetic Analysis

The nucleotide coding sequences (CDS) of the target genes (*Mogat3*, *Dgat2l6*, *Awat1*, *Awat2*, *Elovl3*, and *Fabp9*) were collected from the NCBI (National Center for Biotechnology Information) for a selected set of species representative of all major mammalian lineages (see supplementary material 1: tables S1–S4, Supplementary Material online). To investigate gene distribution in mammals a total of four phylogenetic calculations were performed. The first including *Mogat3* and *Dgat2*; the second including *Awat1*, *Awat2*, and *Dgat2l6*; the third containing *Elovl3* and *Elovl6*; and finally the fourth with *Fabp9*. For this, CDS nucleotide sequences collected in the previous step were translation aligned in the Geneious software (Geneious 7.1.9). Sequence alignment was inspected, and partial sequences were removed. The resulting alignment was subsequently submitted to PhyML3.0 server (Guindon et al. 2010) with the evolutionary model determined automatically using the built in smart model selection (Lefort et al. 2017) for each phylogenetic analysis. Branch support for each phylogenetic analysis was determined using aBayes (Anisimova et al. 2011). The resulting trees were visualized and analyzed in FigTree V1.3.1 available at http://tree.bio.ed.ac.uk/software/figtree/ (supplementary material 1, Supplementary Material online).

### Synteny Analysis and Gene Annotation

Synteny analysis was performed to further assess the orthology of the annotated genes in Cetacea, clarify cases where no gene annotation was found and define genomic regions to be collected for gene annotation. Synteny analysis was performed using as reference *H. sapiens* and *B. taurus locus* of *Mogat3*, *Dgat2l6*, *Awat1*, *Awat2*, *Elovl3*, and *Fabp9* and the available annotated genomes assemblies (*H. sapiens*—GCF_000001405.38 [ID: 109], *B. taurus*—GCF_002263795.1 [ID: 106], *O. orca*—GCF_000331955.1 [ID: 101], *T. truncatus—*GCF_001922835.1 [ID: 101], *D. leucas*—GCF_002288925.1 [ID: 100], *L. vexillifer*—GCF_000442215.1 [ID: 100], *P. catodon*—GCF_002837175.1 [ID: 101], *B. acutorostrata*—GCF_000493695.1 [ID: 100] and *T. latirostris*—GCF_000243295.1 [ID: 102]). For gene annotation, the genomic sequence of the target gene (ranging from the upstream to the downstream flanking genes) was collected from NCBI. For species with no annotated genome available, namely *E. robustus* (GCA_002738545.1), *B. bonaerensis* (GCA_000978805.1), and *H. amphibius* (GCA_002995585.1), the available genome assemblies in NCBI were searched through blastn using as query *B. taurus Mogat3*, *Dgat2l6*, *Awat1*, *Awat2*, *Elovl3*, and *Fabp9*. The best matching genome scaffold was retrieved. Finally for *B. mysticetus*, also with no genome annotation available, genomic sequences containing the target genes were retrieved through blastn searches in the Bowhead Whale Genome Resource (http://www.bowhead-whale.org/; last accessed March 28, 2019) (Keane et al. 2015). The collected genomic sequences were next loaded into Geneious (Geneious 7.1.9). Gene annotation was performed using *B. taurus* genes as reference and for each target gene the corresponding exons were individualized and also loaded into Geneious software (Geneious 7.1.9). The reference exons were mapped onto the corresponding raw genomic nucleotide data with the map to reference tool, the aligned regions were individually analyzed and compared the *B. taurus* exons. Identified ORF-disrupting mutations were annotated and further validated by at least two independent SRA projects per species when available.

### Sample Collection and RNA Extraction for Transcriptomic Analysis

A skin biopsy sample was obtained from a free-ranging blue whale (*B. musculus*) by a Larsen gun (Palsbøll et al. 1991). The sample was obtained in the North Atlantic Ocean, off the coast of Iceland at GPS coordinates: 66.18517, -017.51138 in 2015. The sample was imported to Denmark for examination under Article VII, paragraph 6 CITES convention for import as scientific exchange between CITES institution Natural History Museum of Denmark (DK-003) and University of Iceland (IS 005). A skin biopsy sample was obtained from a Copenhagen Zoo adult male hippopotamus named Jeppe (ZIMS GAN: 27913663) (*H. amphibius*) during a routine veterinary inspection in January 2012, and stored at −80 °C. The samples (two replicates of the skin biopsies) were homogenized using a IKA homogenizer with a plastic dispersing tip. Mixer tips were decontaminated between samples with immersion in 10% sodium hypochlorite, followed by 70% ethanol and 15-min UV light exposure, to avoid cross contamination. Total RNA was purified with Purelink RNA Mini Kit (Invitrogen, Carlsbad, USA) and DNase treated using PureLink DNase (Invitrogen, Carlsbad, USA), following the manufacturer’s instructions with the following modifications. Samples were eluted in 32 µl of RNase free water and incubated at room temperature for 10 min to increase RNA yield. Final RNA sample integrity (RIN, RNA integrity number) and concentration were checked using an Agilent Bioanalyzer 2100 (Agilent Technologies, USA). RNA-seq libraries and sequencing were obtained commercially at Novogene (Hong Kong). After the sequencing, the reads were trimmed and the quality control was done using Trimmomatic v0.36 (Bolger et al. 2014) and FastQC (https://www.bioinformatics.babraham.ac.uk/projects/fastqc/; last accessed March 28, 2019) software (default parameters). Both data sets were submitted to the NCBI SRA (https://www.ncbi.nlm.nih.gov/sra/; last accessed March 28, 2019), under the Bioprojects *H. amphibius*: PRJNA507170 and *B. musculus*: PRJNA507895.

### Transcriptome Assembly and Comparative Analysis

To retrieve skin transcriptomic evidence of the annotated genes in *H. amphibius* and *B. musculus*, two custom local BLAST databases, one per species, were built. These databases were constructed using the skin trimmed sequencing reads of each organism and the makeblastdb application of the BLAST+ application suite. In addition, all skin samples of the *T. truncatus* (BioProject: PRJNA385781) were scrutinized, to obtain evidence of gene expression. Thus, several blastn searches were done, and depending on the organism’s reference sequence, two different strategies were applied. The discontiguous megablast method (dc-megablast) was used whenever the reference sequences and the database belonged to a different species, and megablast was used when both query and database sequence corresponded to the same species. In detail, both megablast and dc-megablast (with the default parameters) were applied to the *H. amphibius* database, with *H. amphibius* (in-house annotated sequences supplementary material 5, Supplementary Material online) and *B. taurus* genes as reference. The same strategy was used to investigate the skin transcriptomic data sets of *T. truncatus*, in NCBI SRA (https://www.ncbi.nlm.nih.gov/sra/; last accessed March 28, 2019), using *T. truncatus* (in-house annotated sequences supplementary material 5, Supplementary Material online) and *B. taurus* as references. On the other hand, only dc-megablast searches were performed on the *B. musculus* database using *B. taurus* and *B. acutorostrata* gene sequences as reference. After screening the transcriptomic data sets, for each species, the entire group of reads with positive matches against the respective target genes, were extracted and loaded into Geneious. Regarding *H. amphibius* and *T. truncatus*, using the map to reference tool from the same software, the collected mRNA reads were mapped against the corresponding in-house annotated genes. In the case of *B. musculus*, using the same tool, the collected reads were mapped against the corresponding *B. taurus* genes. The aligned regions were then manually curated, and poorly aligning reads manually removed. Next, reads were then classified as mature (reads overlapping only exonic regions or spanning over two different exons) and immature (reads containing intronic sequence) (fig. 2*E*).

## Supplementary Material

Supplementary data are available at *Molecular Biology and Evolution* online.

## Supplementary Material

msz068_Supplementary_Material
