# Supplementary material for: Complete Inactivation of Sebum-Producing Genes Parallels the Loss of Sebaceous Glands in Cetacea
Source: Mol Biol Evol. 2019 Mar 20;36(6):1270–80. doi: 10.1093/molbev/msz068 (PMC6526905; doi:10.1093/molbev/msz068)
Supplement: msz068_Supplementary_Material [file msz068_supplementary_material.zip › SUPPLEMENTARY_MATERIAL_1.pdf]

Supplementary Table S1: Accession numbers of *Mogat3* and *Dgat2* sampled in vertebrates and used for phylogenetic analysis. *indicates annotations tagged as Low-quality, Ψ indicates genes found to be pseudogenized.

		Species	Order	Accession number MOGAT3	Accession number DGAT2
**1**	HSA	*Homo sapiens*	Primate-Hominoidae	NM_178176.3	NM_032564.4
**2**	NLE	*Nomascus leucogenys*	Primate-Hominoidae	XM_003276608.3 poor coverage	XM_012505702.1
**3**	MMUL	*Macaca mulatta*	Primate-Cercopithecoidea	XM_015134527.1	XM_015115224.1
**4**	PAN	*Papio anubis*	Primate-Cercopithecoidea	XM_021936509.1 poor coverage	XM_021926332.1
**5**	ANA	*Aotus nancymaae*	Primate-Platyrrhini	XM_012448994.1	XM_012469642.1
**6**	OGA	*Otolemur garnettii*	Primate-Platyrrhini	XM_003794230.1	XM_003780888.3
**7**	TSY	*Tarsius syrichta*	Primate- Tarsiidae	XM_008049431.1	XM_008057821.1
**8**	PCO	*Propithecus coquereli*	Primate- Lemuriformes	XM_012663948.1	XM_012645750.1
**9**	MMUR	*Microcebus murinus*	Primate- Lemuriformes	XM_012759498.1	XM_012744894.1
**10**	NGA	*Nannospalax galili*	Rodentia-Myomorpha	XM_008832401.1Ψ*	XM_008844233.1
**11**	MMU	*Mus musculus*	Rodentia-Myomorpha	EG639992 Ψ	NM_026384.3
**12**	MMA	*Marmota marmota marmota*	Rodentia-Sciuromorpha	XM_015491469.1 poor coverage	XM_015477398.1
**13**	ITR	*Ictidomys tridecemlineatus*	Rodentia-Sciuromorpha	XM_005328487.2 XM_005328488.2 3B	XM_005323214.2
**14**	DOR	*Dipodomys ordii*	Rodentia-Castorimorpha	Not annotated	XM_013014204.1
**15**	CPO	*Cavia porcellus*	Rodentia-Hystricomorpha	XM_023565125.1 Ψ	XM_003468552.4
**16**	HGL	*Heterocephalus glaber*	Rodentia-Hystricomorpha	XM_004840147.2 Ψ	XM_004863213.3
**17**	FDA	*Fukomys damarensis*	Rodentia-Hystricomorpha	XM_010618923.1 Ψ	XM_010612024.2
**18**	OCU	*Oryctolagus cuniculus*	Lagomorpha	XM_002722832.3 XM_008250711.2	XM_008263828.2
**19**	OPR	*Ochotona princeps*	Lagomorpha	XM_004587187.1 XM_012927484.1	XM_004589878.2
**20**	SSC	*Sus scrofa*	Cetartiodactyla- Suina	XM_021087978.1 Ψ XM_021086259.1 3B	NM_001160080.1
**21**	CDR	*Camelus dromedarius*	Cetartiodactyla- Camelidae	XM_010984066.1 Ψ	XM_010989763.1
**22**	BTA	*Bos taurus*	Cetartiodactyla- Ruminantia-Bovinae	XM_024985259.1	NM_205793.2
**23**	OAR	*Ovis aries*	Cetartiodactyla- Ruminantia-Caprinae	XM_004020997.3 XM_004020996.3	XM_012096078.2
**24**	CHI	*Capra hircus*	Cetartiodactyla- Ruminantia-Caprinae	XM_013976798.2	XM_018058853.1
**25**	OOR	*Orcinus orca*	Cetartiodactyla-Cetacea-Odontoceti	XM_012532584.1 Ψ*	XM_004279809.2
**26**	TTR	*Tursiops truncatus*	Cetartiodactyla-Cetacea-Odontoceti	Not annotated	XM_019946545.1
**27**	DLE	*Delphinapterus leucas*	Cetartiodactyla-Cetacea-Odontoceti	XM_022588621.1 Ψ *	XM_022593852.1
**28**	LVE	*Lipotes vexillifer*	Cetartiodactyla-Cetacea-Odontoceti	Not annotated	
**29**	PCA	*Physeter catodon*	Cetartiodactyla-Cetacea-Mysticeti	XM_024125870.1 Ψ *	XM_007118120.2
**30**	BAC	*Balaenoptera acutorostrata*	Cetartiodactyla-Cetacea-Mysticeti	XM_007186602.1 Ψ *	XM_007194068.1
**31**	BBO	*Balaenoptera bonaerensis*	Cetartiodactyla-Cetacea-Mysticeti	Not annotated	
**32**	ERO	*Eschrichtius robustus*	Cetartiodactyla-Cetacea-Mysticeti	Not annotated	
**33**	BMY	*Balaena mysticetus*	Cetartiodactyla-Cetacea-Mysticeti	Not annotated	
**34**	HAM	*Hippopotamus amphibius*	Cetartiodactyla- Hippopotamidae	Predicted in this work Ψ	
**35**	CSI	*Ceratotherium simum simum*	Perissodactyla-Rhinoceratidae	XM_014796527.1 Ψ*	XM_004434014.2
**36**	ECA	*Equus caballus*	Perissodactyla-Equidea	XM_023616872.1 Ψ*	XM_023645689.1
**37**	EPR	*Equus przewalskii*	Perissodactyla-Equidea	XM_008543533.1 Ψ*	XM_008515902.1
**38**	CFA	*Canis lupus familiaris*	Carnivora	XM_845212.5	XM_022407666.1
**39**	FCA	*Felis catus*	Carnivora	XM_006942010.4	XM_011286632.2
**40**	PVA	*Pteropus vampyrus*	Chiroptera	XM_023537499.1 Ψ*	XM_011368400.2
**41**	MDA	*Myotis davidii*	Chiroptera	XM_015570281.1	XM_006777907.2
**42**	TMA	*Trichechus manatus latirostris*	Afrotheria- Sirenia	XM_023740983.1 Ψ	XM_023736570.1
**43**	ETE	*Echinops telfairi*	Afrotheria-Tenrecidae	XM_013005436.1 poor coverage	
**44**	CAS	*Chrysochloris asiatica*	Afrotheria-Afrosoricida	XM_006859824.1	XM_006875437.1
**45**	OAF	*Orycteropus afer afer*	Afrotheria-Tubulidentata	XM_007942285.1	XM_007940923.1
**46**	LAF	*Loxodonta africana*	Afrotheria-Proboscidea	XM_023555826.1 Ψ	
**47**	EED	*Elephantulus edwardii*	Afrotheria-Macroscelidea	XM_006889805.1*	XM_006882862.1
**48**	TCH	*Tupaia chinensis*	Scandentia	XM_006155742.1	XM_006141009.2
**49**	GVA	*Galeopterus variegatus*	Dermoptera	XM_008589478.1 3A* XM_008589483.1 3B	XM_008577261.1
**50**	MJA	*Manis javanica*	Pholidota	XM_017658278.1 3A Ψ* XM_017658292.1 3B	XM_017672083.1
**51**	CCR	*Condylura cristata*	Insectivora-Talpidae	XM_004688259.1	XM_004683213.1
**52**	SAR	*Sorex araneus*	Insectivora-Eulipotyphla	XM_004620900.1	XM_012933260.1
**53**	EEU	*Erinaceus europaeus*	Insectivora- Erinaceomorpha	XM_007517983.2	XM_016186910.1
**54**	DNO	*Dasypus novemcinctus*	Xenartha-Cingulata	XM_004482131.1 3A Ψ* XM_012521042.1 3B	
**55**	SHA	*Sarcophilus harrisii*	Marsupialia	XM_003768836.2	XM_003764648.3
**56**	MDO	*Monodelphis domestica*	Marsupialia	XM_007483270.2	XM_007490952.2
**57**	OAN	*Ornithorhynchus anatinus*	Monotremata		
**58**	GGA	*Gallus gallus*	Birds		XM_419374.6

**59**	MGA	*Meleagris gallopavo*	Birds		XM_010706269.2
**60**	APL	*Anas platyrhynchos*	Birds		XM_005024101.3
**61**	FAL	*Ficedula albicollis*	Birds		ENSFALT00000012493.1
**62**	ACA	*Anolis carolinensis*	Reptile		XM_008117776.2
**63**	AMI	*Alligator mississippiensis*	Reptile		
**64**	PSI	*Pelodiscus sinensis*	Reptile	XM_006127277.2	
**65**	XTR	*Xenopus tropicalis*	Amphibia		NM_204041.2

Supplementary Table S2: Accession numbers of *Awat1*, *Awat2* and *Dgat2l6* sampled in vertebrates and used for phylogenetic analysis. *indicates annotations tagged as low-quality, Ψ indicates genes found to be pseudogenized.

		Species	Order	AWAT1	AWAT2	DGAT2L6
**1**	HSA	*Homo sapiens*	Primate-Hominoidae	NM_001013579.2	XM_011530876.2	NM_198512.2
**2**	NLE	*Nomascus leucogenys*	Primate-Hominoidae	XM_003272676.2 poor annotation	XM_003272673.1	XM_003272675.1
**3**	MMUL	*Macaca mulatta*	Primate-Cercopithecoidea	XM_001083656.2	XM_001085075.3	XM_001083431.3
**4**	PAN	*Papio anubis*	Primate-Cercopithecoidea	XM_021933015.1 poor annotation	XM_009197760.3	XM_003917833.3
**5**	ANA	*Aotus nancymaae*	Primate-Platyrrhini	XM_012460813.1	XM_012460817.2	XM_021673167.1
**6**	OGA	*Otolemur garnettii*	Primate-Platyrrhini	XM_003801766.1	XM_003801768.2	XM_003801774.1
**7**	TSY	*Tarsius syrichta*	Primate- Tarsiidae	XM_008058814.1	XM_008058821.2	XM_008058816.1
**8**	PCO	*Propithecus coquereli*	Primate- Lemuriformes	XM_012643436.1	XM_012643429.1	XM_012642951.1
**9**	MMUR	*Microcebus murinus*	Primate- Lemuriformes		XM_012736247.1	XM_020285060.1
**10**	NGA	*Nannospalax galili*	Rodentia-Myomorpha	XM_008844385.1	XM_008844383.2	XM_008844403.2*
**11**	MMU	*Mus musculus*	Rodentia-Myomorpha	NM_001081136.1	NM_177746.4	NM_001114084.1
**12**	MMA	*Marmota marmota marmota*	Rodentia-Sciuromorpha	XM_015501605.1	XM_015501608.1	XM_015501606.1
**13**	ITR	*Ictidomys tridecemlineatus*	Rodentia-Sciuromorpha	XM_005339988.2	XM_005340168.2	ENSSTOT00000028225.1
**14**	DOR	*Dipodomys ordii*	Rodentia-Castorimorpha	XM_013031611.1		XM_013031610.1
**15**	CPO	*Cavia porcellus*	Rodentia-Hystricomorpha	XM_003471013.1	XM_003471016.2	ENSCPOT00000004036.3
**16**	HGL	*Heterocephalus glaber*	Rodentia-Hystricomorpha	XM_004872602.2	XM_021249864.1Ψ	XM_004872679.2
**17**	FDA	*Fukomys damarensis*	Rodentia-Hystricomorpha	XM_010632168.1	XM_010632173.	XM_010632219.1*Ψ
**18**	OCU	*Oryctolagus cuniculus*	Lagomorpha	XM_002720072.3	XM_002720065.3	XM_008272742.2
**19**	OPR	*Ochotona princeps*	Lagomorpha	XM_004595221.2	XM_004595219.1	XM_004595220.1
**20**	SSC	*Sus scrofa*	Cetartiodactyla- Suina	XM_003484124.1*Ψ	XM_021080857.1*Ψ	ENSSSCT00000040022.1
**21**	CDR	*Camelus dromedarius*	Cetartiodactyla- Camelidae	XM_010978745.1	XM_010978767.1 poor coverage	XM_010978744.1
**22**	BTA	*Bos taurus*	Cetartiodactyla- Ruminantia-Bovinae	NM_001192683.1	XM_002700046.5	NM_001101861.1
**23**	OAR	*Ovis aries*	Cetartiodactyla- Ruminantia-Caprinae	XM_012142233.1	XM_004022623.3	XM_012142235.1
**24**	CHI	*Capra hircus*	Cetartiodactyla- Ruminantia-Caprinae	XM_005700705.3	XM_005700711.3	XM_005700706.2
**25**	OOR	*Orcinus orca*	Cetartiodactyla-Cetacea-Odontoceti	Not annotated Ψ	XM_004275829.1* Ψ	XM_004275782.1*Ψ
**26**	TTR	*Tursiops truncatus*	Cetartiodactyla-Cetacea-Odontoceti	Not annotated Ψ	XM_019948891.1 *Ψ	XM_019948854.1*Ψ
**27**	DLE	*Delphinapterus leucas*	Cetartiodactyla-Cetacea-Odontoceti	Not annotated Ψ	Not annotated Ψ	Not annotated
**28**	LVE	*Lipotes vexillifer*	Cetartiodactyla-Cetacea-Odontoceti	Not annotated Ψ	XM_007470150.1 *Ψ	XM_007472333.1*Ψ
**29**	PCA	*Physeter catodon*	Cetartiodactyla-Cetacea-Mysticeti	Not annotated Ψ	XM_007119597.2 *Ψ	XM_024116553.1*Ψ
**30**	BAC	*Balaenoptera acutorostrata*	Cetartiodactyla-Cetacea-Mysticeti	Not annotated Ψ	XM_007184960.1 Ψ	XM_007198671.1*Ψ
**31**	BBO	*Balaenoptera bonaerensis*	Cetartiodactyla-Cetacea-Mysticeti	Not annotated Ψ	Not annotated Ψ	Not annotated
**32**	ERO	*Eschrichtius robustus*	Cetartiodactyla-Cetacea-Mysticeti	Not annotated Ψ	Not annotated Ψ	Not annotated
**33**	BMY	*Balaena mysticetus*	Cetartiodactyla-Cetacea-Mysticeti	Not annotated Ψ	Not annotated Ψ	Not annotated
**34**	HAM	*Hippopotamus amphibius*	Cetartiodactyla-Hippopotamidae	Predicted in this work Ψ	Predicted in this work	Predicted in this work
**35**	CSI	*Ceratotherium simum simum*	Perissodactyla-Rhinoceratidae	XM_004439879.2 Ψ	XM_004439882.2	XM_004439922.2Ψ
**36**	ECA	*Equus caballus*	Perissodactyla-Equidea	XM_001490112.4	XM_001496730.3	XM_001490803.4
**37**	EPR	*Equus przewalskii*	Perissodactyla-Equidea	XM_008507290.1	XM_008508126.1	XM_008507291.1
**38**	CFA	*Canis lupus familiaris*	Carnivora	XM_549058.3	XM_549056.3	XM_844262.4
**39**	FCA	*Felis catus*	Carnivora	XM_004000591.4	XM_004000588.4	XM_004000645.2
**40**	PVA	*Pteropus vampyrus*	Chiroptera	XM_011371445.1	XM_023537450.1*Ψ	XM_011371446.1
**41**	MDA	*Myotis davidii*	Chiroptera	XM_006766595.2	XM_006779554.2Ψ	KB109555.1
**42**	TMA	*Trichechus manatus latirostris*	Afrotheria- Sirenia	Predicted in this work	XM_012556621.1 Ψ	Not annotated poor coverage
**43**	ETE	*Echinops telfairi*	Afrotheria-Tenrecidae	XM_004716689.1	Not annotated poor coverage	XM_004716690.1
**44**	CAS	*Chrysochloris asiatica*	Afrotheria-Afrosoricida	XM_006868518.1	Not annotated poor coverage	XM_006868517.1*Ψ
**45**	OAF	*Orycteropus afer afer*	Afrotheria-Tubulidentata	XM_007956990.1	LOC103211074 Ψ	XM_007956994.1
**46**	LAF	*Loxodonta africana*	Afrotheria-Proboscidea	XM_003412737.3	XM_023551399.1*Ψ	XM_003412684.3
**47**	EED	*Elephantulus edwardii*	Afrotheria-Macroscelidea	XM_006895961.1	Not annotated poor coverage	XM_006895960.1
**48**	TCH	*Tupaia chinensis*	Scandentia	XM_006166656.1	XM_006169972.1	XM_006169968.1

**49**	GVA	*Galeopterus variegatus*	Dermoptera	XM_010632168.1	XM_008580330.1	XM_008580343.1*Ψ
**50**	MJA	*Manis javanica*	Pholidota	Not annotated poor coverage	Not annotated poor coverage	XM_017670833.1*Ψ
**51**	CCR	*Condylura cristata*	Insectivora-Talpidae	XM_004694454.1	XM_004694429.2	XM_004694430.2
**52**	SAR	*Sorex araneus*	Insectivora-Eulipotyphla	XM_004615754.1	Not annotated	XM_004615774.1
**53**	EEU	*Erinaceus europaeus*	Insectivora- Erinaceomorpha	XM_007530768.1*Ψ	XM_007530767.1	XM_016191553.1
**54**	DNO	*Dasypus novemcinctus*	Xenartha-Cingulata	XM_004475923.1	XM_004474301Ψ	Not annotated poor coverage
**55**	SHA	*Sarcophilus harrisii*	Marsupialia	ENSSHAT00000007086.1	XM_003774960.2	XM_003774967
**56**	MDO	*Monodelphis domestica*	Marsupialia	ENSMODT00000005962.3	XM_003342312.3*	XM_007507615.2
**57**	OAN	*Ornithorhynchus anatinus*	Monotremata	Not annotated poor coverage	Not annotated poor coverage	Not annotated poor coverage
**58**	GGA	*Gallus gallus*	Birds	NM_001318429.1		
**59**	MGA	*Meleagris gallopavo*	Birds		XM_010715099.2 (DGAT2)	
**60**	APL	*Anas platyrhynchos*	Birds		XM_013099502.2 (DGAT2) poor coverage	
**61**	FAL	*Ficedula albicollis*	Birds		XM_016298199.2 (DGAT2)	
**62**	ACA	*Anolis carolinensis*	Reptile		XM_008123819.2* poor coverage	
**63**	AMI	*Alligator mississippiensis*	Reptile		XM_006266229.3 (DGAT2)	
**64**	PSI	*Pelodiscus sinensis*	Reptile		XM_006113680.3 (DGAT2)	
**65**	XTR	*Xenopus tropicalis*	Amphibia			XM_002934893.4
**66**	XLA	*Xenopus laevis*	Amphibia			XM_018229711.1 XM_002934893.4

Supplementary Table S3: Accession numbers of *Elovl3* and *Elovl6* sampled in vertebrates and used for phylogenetic analysis. *indicates annotations tagged as low-quality, Ψ indicates genes found to be pseudogenized.

		Species	Order	Accession number ELOVL3	Accession number ELOVL6
**1**	HSA	*Homo sapiens*	Primate-Hominoidae	NM_152310.2	NM_024090.2
**2**	NLE	*Nomascus leucogenys*	Primate-Hominoidae	XM_003255389.2	XM_012502811.1
**3**	MMUL	*Macaca mulatta*	Primate-Cercopithecoidea	NM_001194552.1	NM_001266921.1
**4**	PAN	*Papio anubis*	Primate-Cercopithecoidea	XM_003904175.3	XM_017958964.2
**5**	ANA	*Aotus nancymaae*	Primate-Platyrrhini	XM_012439308.1	XM_012436581.2
**6**	OGA	*Otolemur garnettii*	Primate-Platyrrhini	XM_012803584.2	XM_012810357.2
**7**	TSY	*Tarsius syrichta*	Primate- Tarsiidae	XM_008048436.2<br>Partial poor coverage	XM_021716255.1
**8**	PCO	*Propithecus coquereli*	Primate- Lemuriformes	XM_012661683.1	XM_012655092.1<br>Partial poor coverage
**9**	MMUR	*Microcebus murinus*	Primate- Lemuriformes	XM_012769725.2	XM_012766192.2
**10**	NGA	*Nannospalax galili*	Rodentia-Myomorpha	XM_008845631.1	XM_008843449.2
**11**	MMU	*Mus musculus*	Rodentia-Myomorpha	NM_007703.2	NM_130450.2
**12**	MMA	*Marmota marmota marmota*	Rodentia-Sciuromorpha	XM_015494898.1	Not annotated poor coverage
**13**	ITR	*Ictidomys tridecemlineatus*	Rodentia-Sciuromorpha	XM_005333633.3	XM_013360685.2
**14**	DOR	*Dipodomys ordii*	Rodentia-Castorimorpha	XM_013017592.1	XM_013023725.1
**15**	CPO	*Cavia porcellus*	Rodentia-Hystricomorpha	XM_003474994.3	XM_013150609.2
**16**	HGL	*Heterocephalus glaber*	Rodentia-Hystricomorpha	XM_004866397.3	XM_004867583.3
**17**	FDA	*Fukomys damarensis*	Rodentia-Hystricomorpha	XM_010626409.2	XM_010641599.2<br>Partial poor coverage
**18**	OCU	*Oryctolagus cuniculus*	Lagomorpha	XM_002718575.3	
**19**	OPR	*Ochotona princeps*	Lagomorpha	XM_004579916.1	XM_004594525.1
**20**	SSC	*Sus scrofa*	Cetartiodactyla- Suina	NM_001167634.1	XM_021100705.1
**21**	CDR	*Camelus dromedarius*	Cetartiodactyla- Camelidae	XM_010974486.1	XM_010981089.1
**22**	BTA	*Bos taurus*	Cetartiodactyla- Ruminantia-Bovinae		NM_001102155.1
**23**	OAR	*Ovis aries*	Cetartiodactyla- Ruminantia-Caprinae	XM_012102841.2	XM_004009618.3
**24**	CHI	*Capra hircus*	Cetartiodactyla- Ruminantia-Caprinae	XM_005698356.2	XM_005681307.3
**25**	OOR	*Orcinus orca*	Cetartiodactyla-Cetacea-Odontoceti	XM_004268506.1* Ψ	XM_004269571.2
**26**	TTR	*Tursiops truncatus*	Cetartiodactyla-Cetacea-Odontoceti	XM_004318260.2* Ψ	XM_019927806.1
**27**	DLE	*Delphinapterus leucas*	Cetartiodactyla-Cetacea-Odontoceti	Not annotated Ψ	XM_022596434.1
**28**	LVE	*Lipotes vexillifer*	Cetartiodactyla-Cetacea-Odontoceti	XM_007470393.1* Ψ	XM_007463221.1
**29**	PCA	*Physeter catodon*	Cetartiodactyla-Cetacea-Mysticeti	XM_007126871.1* Ψ	XM_007105849.2
**30**	BAC	*Balaenoptera acutorostrata*	Cetartiodactyla-Cetacea-Mysticeti	XM_007187279.1* Ψ	XM_007191038.1
**31**	BBO	*Balaenoptera bonaerensis*	Cetartiodactyla-Cetacea-Mysticeti	Not annotated Ψ	Not annotated
**32**	ERO	*Eschrichtius robustus*	Cetartiodactyla-Cetacea-Mysticeti	Not annotated Ψ	Not annotated
**33**	BMY	*Balaena mysticetus*	Cetartiodactyla-Cetacea-Mysticeti	Not annotated Ψ	Not annotated
**34**	HAM	*Hippopotamus amphibius*	Cetartiodactyla- Hippopotamidae	Predicted in this work	Not annotated
**35**	CSI	*Ceratotherium simum simum*	Perissodactyla-Rhinoceratidae	XM_004427998.2	XM_014784594.1
**36**	ECA	*Equus caballus*	Perissodactyla-Equidea	XM_001499143.5	XM_005607891.3
**37**	EPR	*Equus przewalskii*	Perissodactyla-Equidea	XM_008507786.1	XM_008539516.1
**38**	CFA	*Canis lupus familiaris*	Carnivora	ENSCAFT00000044364.1	XM_005639271.3
**39**	FCA	*Felis catus*	Carnivora	XM_003994357.4	XM_003985082.5
**40**	PVA	*Pteropus vampyrus*	Chiroptera	XM_011378910.2	XM_011361434.2
**41**	MDA	*Myotis davidii*	Chiroptera	XM_006756493.2	XM_006779570.2*
**42**	TMA	*Trichechus manatus latirostris*	Afrotheria- Sirenia	XM_004370075.2	XM_004380239.2
**43**	ETE	*Echinops telfairi*	Afrotheria-Tenrecidae	XM_004700963.1	XM_013004877.1
**44**	CAS	*Chrysochloris asiatica*	Afrotheria-Afrosoricida	XM_006831285.1	XM_006869315.1
**45**	OAF	*Orycteropus afer afer*	Afrotheria-Tubulidentata	XM_007939907.1	XM_007940750.1
**46**	LAF	*Loxodonta africana*	Afrotheria-Proboscidea	XM_023546790.1<br>Partial poor coverage	XM_023548580.1
**47**	EED	*Elephantulus edwardii*	Afrotheria-Macroscelidea	XM_006879988.1	XM_006881090.1
**48**	TCH	*Tupaia chinensis*	Scandentia	XM_006166342.1	XM_014593154
**49**	GVA	*Galeopterus variegatus*	Dermoptera	XM_008569694.1	XM_008570178.1
**50**	MJA	*Manis javanica*	Pholidota	XM_017674341.1	XM_017676120.1
**51**	CCR	*Condylura cristata*	Insectivora-Talpidae	XM_004681009.2	XM_012727973.1
**52**	SAR	*Sorex araneus*	Insectivora-Eulipotyphla	XM_004616402.1<br>Partial poor coverage	XM_004612761.1<br>Partial poor coverage
**53**	EEU	*Erinaceus europaeus*	Insectivora- Erinaceomorpha	XM_007531049.2	XM_007530779.2
**54**	DNO	*Dasypus novemcinctus*	Xenartha-Cingulata	XM_004478871.3	XM_004476163.2
**55**	SHA	*Sarcophilus harrisii*	Marsupialia	ENSSHAT00000022499.1<br>Partial poor coverage	XM_003772988.3
**56**	MDO	*Monodelphis domestica*	Marsupialia	XM_007479117.2<br>Partial poor coverage	XM_007481027.2
**57**	OAN	*Ornithorhynchus anatinus*	Monotremata	XM_016228601.1<br>Partial poor coverage	XM_003428607.2<br>Partial poor coverage
**58**	GGA	*Gallus gallus*	Birds	NM_001318410.1	HQ264103.1
**59**	MGA	*Meleagris gallopavo*	Birds	XM_010714424.2	XM_010710094.2

					Partial poor coverage
**60**	APL	*Anas platyrhynchos*	Birds	XM_005025218.2	XM_021272653.1<br>Partial poor coverage
**61**	FAL	*Ficedula albicollis*	Birds	XM_005048626.2	XM_005044995.1
**62**	ACA	*Anolis carolinensis*	Reptile	Not annotated	XM_003221752.3
**63**	AMI	*Alligator mississippiensis*	Reptile	XM_006276236.3	XM_006264078.3
**64**	PSI	*Pelodiscus sinensis*	Reptile	Not annotated	XM_006119276.2
**65**	XTR	*Xenopus tropicalis*	Amphibia	XM_002935763.4	NM_001017257.3
**66**	XLA	*Xenopus laevis*	Amphibia	XM_018227602.1 (7s)<br>XM_018225418.1 (7L)	XM_018243349.1 (1S)<br>XM_018251326.1 (1L)
**67**	LCH	*Latimeria chalumnae*	Coelacanthiformes	XM_005995138	XM_006011026.2
**68**	DRE	*Danio rerio*	Teleostei- Otomorpha	ENSDART00000007202.9<br>NM_201500.1	NM_199532.1
**69**	AME	*Astyanax mexicanus*	Teleostei- Otomorpha	ENSAMXT00000004293.1	XM_007245513.2
**70**	SFO	*Scleropages formosus*	Teleostei- Osteoglossomorpha		XM_018759499.1
**71**	LOC	*Lepisosteus oculatus*	Holostei-	ENSLOCT00000015292.1	XM_006629830.2
**72**	RTY	*Rhincodon typus*	Chondrichthyes- Elasmobranchii	XM_020529222.1	
**73**	CMI	*Callorhinchus milii*	Chondrichthyes- Chimaeriformes	XM_007899814.1	XM_007896995.1
**74**	BFL	*Branchiostoma floridae*	Cephalochordata	XM_002601874.1	
**75**	BLA	*Branchiostoma belcheri*	Cephalochordata	XM_019783923.1	

Supplementary Table S4: Accession numbers of *FABP9* sampled in vertebrates and used for phylogenetic analysis. *indicates annotations tagged as Low-quality, Ψ indicates genes found to be pseudogenized.

		Species	Order	Accession numbers FABP9
**1**	HSA	*Homo sapiens*	Primate-Hominoidae	NM_001080526.1
**2**	NLE	*Nomascus leucogenys*	Primate-Hominoidae	XM_003269489.1
**3**	MMUL	*Macaca mulatta*	Primate-Cercopithecoidea	NM_001193941.1
**4**	PAN	*Papio anubis*	Primate-Cercopithecoidea	XM_003831269.1
**5**	ANA	*Aotus nancymaae*	Primate-Platyrrhini	Partial
**6**	OGA	*Otolemur garnettii*	Primate-Platyrrhini	XM_003782526.1
**7**	TSY	*Tarsius syrichta*	Primate- Tarsiidae	XM_008053298.1
**8**	PCO	*Propithecus coquereli*	Primate- Lemuriformes	XM_012663152.1
**9**	MMUR	*Microcebus murinus*	Primate- Lemuriformes	XM_012740033.1
**10**	NGA	*Nannospalax galili*	Rodentia-Myomorpha	XM_008826074.1
**11**	MMU	*Mus musculus*	Rodentia-Myomorpha	NM_011598.3
**12**	MMA	*Marmota marmota marmota*	Rodentia-Sciuromorpha	XM_015485909.1
**13**	ITR	*Ictidomys tridecemlineatus*	Rodentia-Sciuromorpha	XM_013363204.2
**14**	DOR	*Dipodomys ordii*	Rodentia-Castorimorpha	XM_013012447.1
**15**	HGL	*Heterocephalus glaber*	Rodentia-Hystricomorpha	XM_004842003.1
**16**	FDA	*Fukomys damarensis*	Rodentia-Hystricomorpha	XM_010636255.1
**17**	OCU	*Oryctolagus cuniculus*	Lagomorpha	XM_002710656.3
**18**	OPR	*Ochotona princeps*	Lagomorpha	XM_004588077.1
**19**	SSC	*Sus scrofa*	Cetartiodactyla- Suina	Not annotated
**20**	VPA	*Vicugna pacos*	Cetartiodactyla- Camelidae	XM_006202374.2
**21**	CDR	*Camelus dromedarius*	Cetartiodactyla- Camelidae	XM_010979387.1
**22**	BTA	*Bos taurus*	Cetartiodactyla- Ruminantia-Bovinae	NM_001192410.1
**23**	OAR	*Ovis aries*	Cetartiodactyla- Ruminantia-Caprinae	XM_004011763.2
**24**	CHI	*Capra hircus*	Cetartiodactyla- Ruminantia-Caprinae	XM_005689129.3
**25**	OOR	*Orcinus orca*	Cetartiodactyla-Cetacea-Odontoceti	Not annotated Ψ
**26**	TTR	*Tursiops truncatus*	Cetartiodactyla-Cetacea-Odontoceti	Not annotated Ψ
**27**	DLE	*Delphinapterus leucas*	Cetartiodactyla-Cetacea-Odontoceti	Not annotated Ψ
**28**	LVE	*Lipotes vexillifer*	Cetartiodactyla-Cetacea-Odontoceti	Not annotated Ψ
**29**	PCA	*Physeter catodon*	Cetartiodactyla-Cetacea-Mysticeti	Not annotated Ψ
**30**	BAC	*Balaenoptera acutorostrata*	Cetartiodactyla-Cetacea-Mysticeti	XM_007185102.1 Ψ
**31**	BBO	*Balaenoptera bonaerensis*	Cetartiodactyla-Cetacea-Mysticeti	Not annotated Ψ
**32**	ERO	*Eschrichtius robustus*	Cetartiodactyla-Cetacea-Mysticeti	Not annotated Ψ
**33**	BMY	*Balaena mysticetus*	Cetartiodactyla-Cetacea-Mysticeti	Not annotated Ψ
**34**	HAM	*Hippopotamus amphibius*	Cetartiodactyla- Hippopotamidae	Not annotated Ψ
**35**	CSI	*Ceratotherium simum simum*	Perissodactyla-Rhinoceratidae	XM_004427146.2 Ψ
**36**	ECA	*Equus caballus*	Perissodactyla-Equidea	XM_001489404.4
**34**	EPR	*Equus przewalskii*	Perissodactyla-Equidea	XM_008535214.1
**35**	CFA	*Canis lupus familiaris*	Carnivora	XM_544145.4
**36**	FCA	*Felis catus*	Carnivora	XM_003999922.5
**37**	MLU	*Myotis lucifugus*	Chiroptera	XM_006102144.3
**38**	MDA	*Myotis davidii*	Chiroptera	XM_006759136.2
**39**	TMA	*Trichechus manatus latirostris*	Afrotheria- Sirenia	XM_004385997.2
**40**	ETE	*Echinops telfairi*	Afrotheria-Tenrecidae	XM_004697496.1
**41**	CAS	*Chrysochloris asiatica*	Afrotheria-Afrosoricida	XM_006830810.1
**42**	OAF	*Orycteropus afer afer*	Afrotheria-Tubulidentata	XM_007944657.1
**43**	LAF	*Loxodonta africana*	Afrotheria-Proboscidea	XM_003408364.2
**44**	EED	*Elephantulus edwardii*	Afrotheria-Macroscelidea	XM_006880493.1
**45**	TCH	*Tupaia chinensis*	Scandentia	XM_006168932.1
**46**	GVA	*Galeopterus variegatus*	Dermoptera	XM_008580732.1
**47**	MJA	*Manis javanica*	Pholidota	Not annotated
**48**	CCR	*Condylura cristata*	Insectivora-Talpidae	XM_004679772.2
**49**	SAR	*Sorex araneus*	Insectivora-Eulipotyphla	XM_004602370.1
**50**	EEU	*Erinaceus europaeus*	Insectivora- Erinaceomorpha	XM_007520145.2
**51**	DNO	*Dasypus novemcinctus*	Xenartha-Cingulata	XM_012519860.1
**52**	SHA	*Sarcophilus harrisii*	Marsupialia	XM_003759678.1
**53**	MDO	*Monodelphis domestica*	Marsupialia	XM_001367113.3

Phylogenetic and synteny analysis

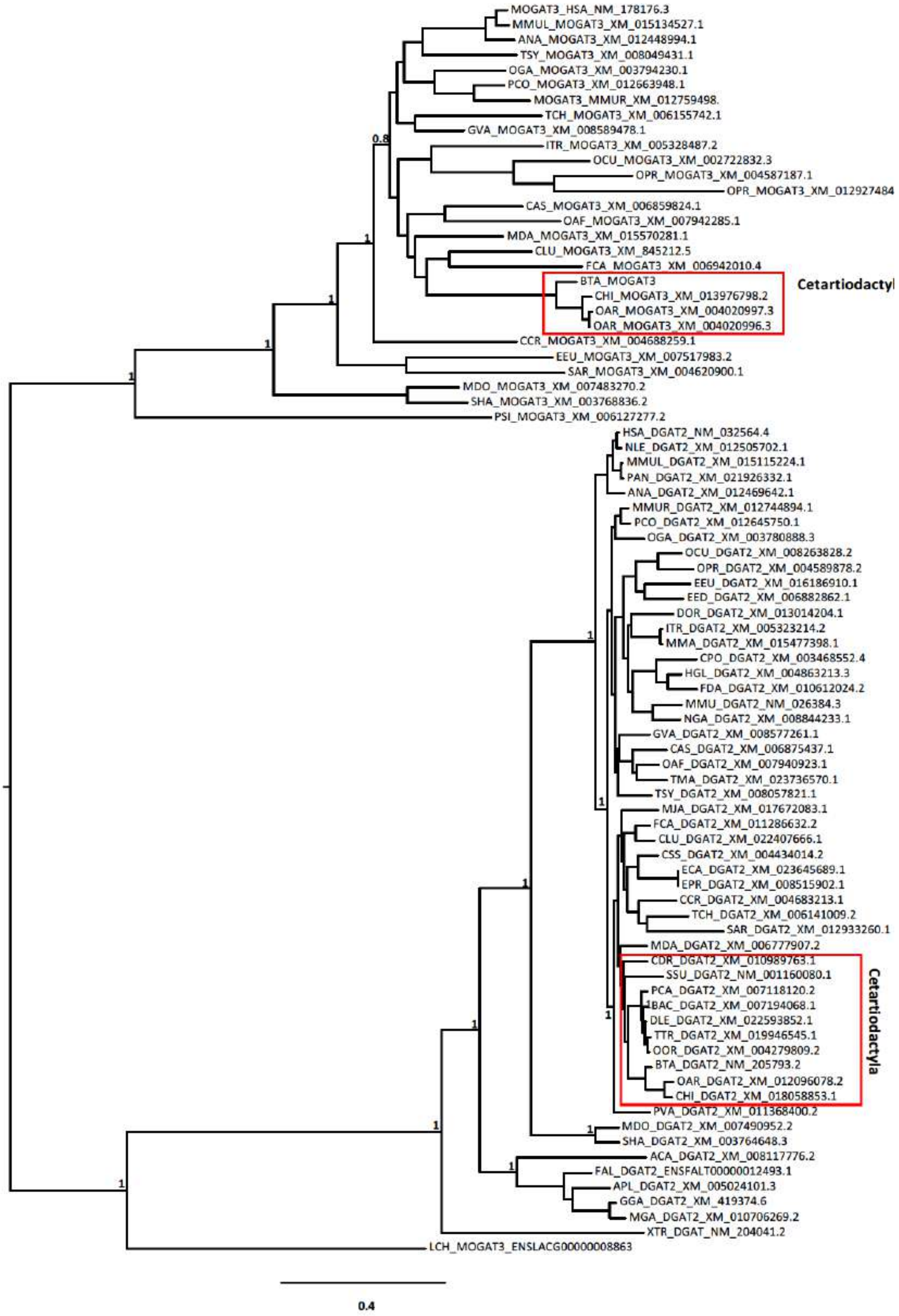

Supplementary Figure S1 - Maximum likelihood phylogenetic analysis of DGAT2 and MOGAT3, branch support calculated using aBayes values at node indicate posterior probabilities and are shown only in the major branches. The analysis comprised 83 taxa, 1573 positions and the best fitting evolutionary model was GTR +G+I. Species acronyms are available in corresponding supplementary table S1.

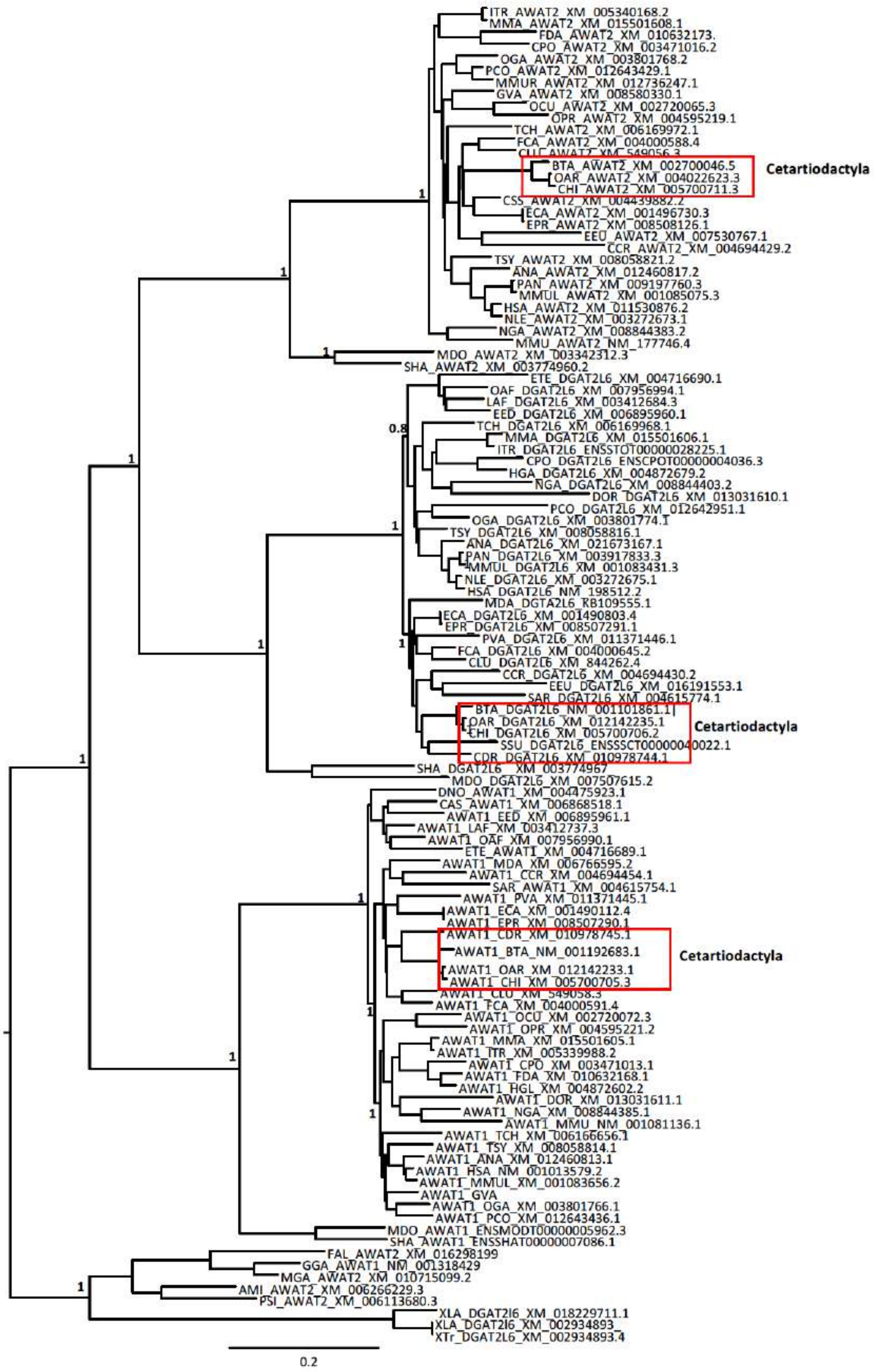

Supplementary Figure S2 - Maximum likelihood phylogenetic analysis of AWAT1, AWAT2 and DGAT2L6 branch support calculated using aBayes values at node indicate posterior probabilities and are shown only in the major branches. The analysis comprised 113 taxa, 1124 positions and the best fitting evolutionary model was GTR +G+I. Species acronyms are available in corresponding supplementary table S1. Species acronyms are available in corresponding supplementary table S2.

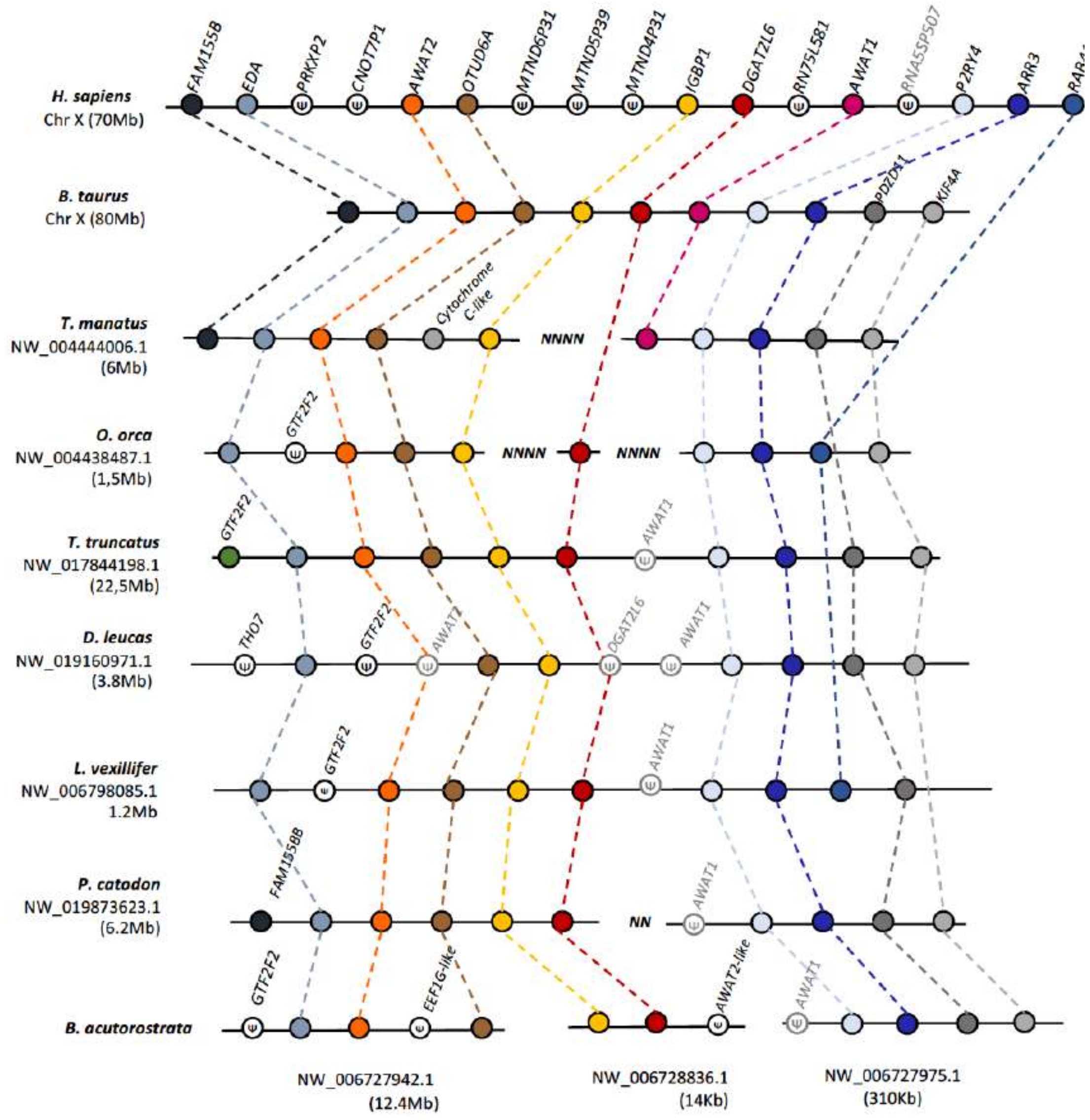

Supplementary Figure S3- Comparative synteny analysis of *Awat1 Awat2* and *Dgat2l6*.

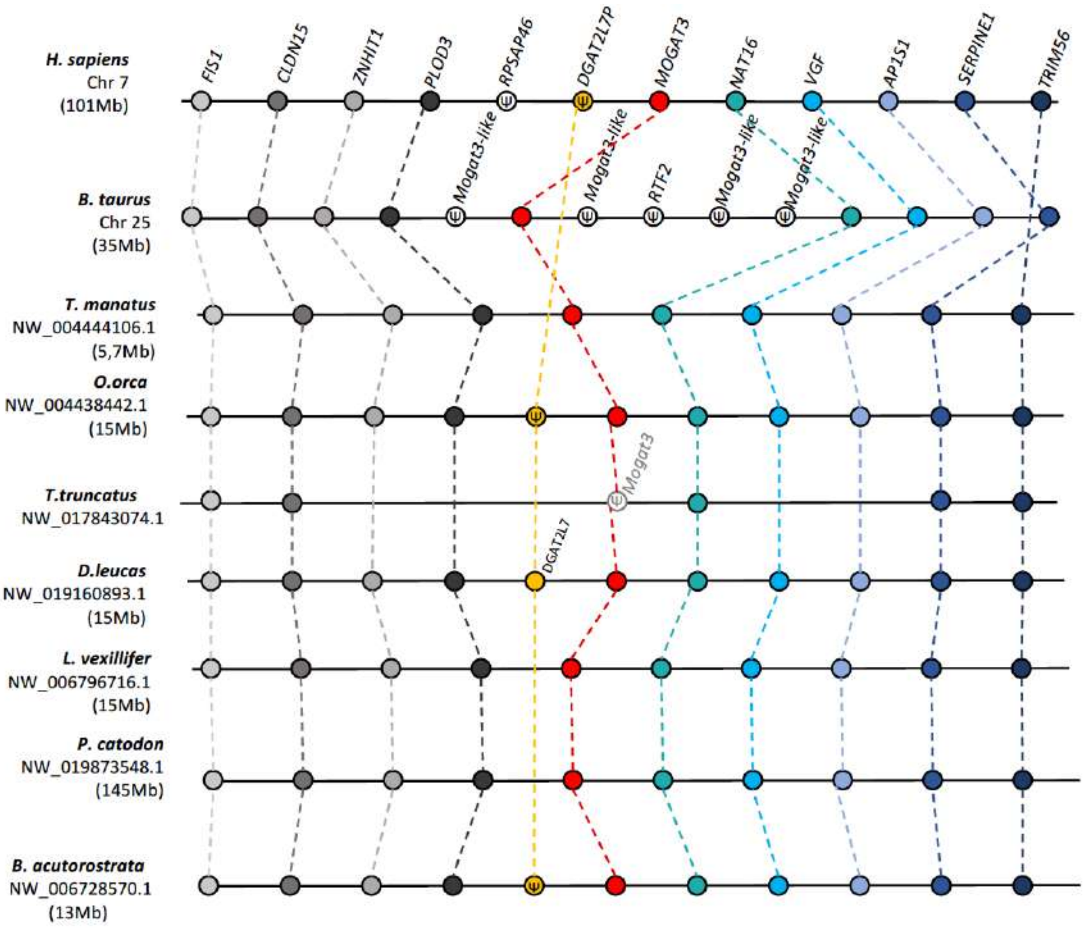

Supplementary Figure S4- Comparative synteny analysis of *Mogat3*.

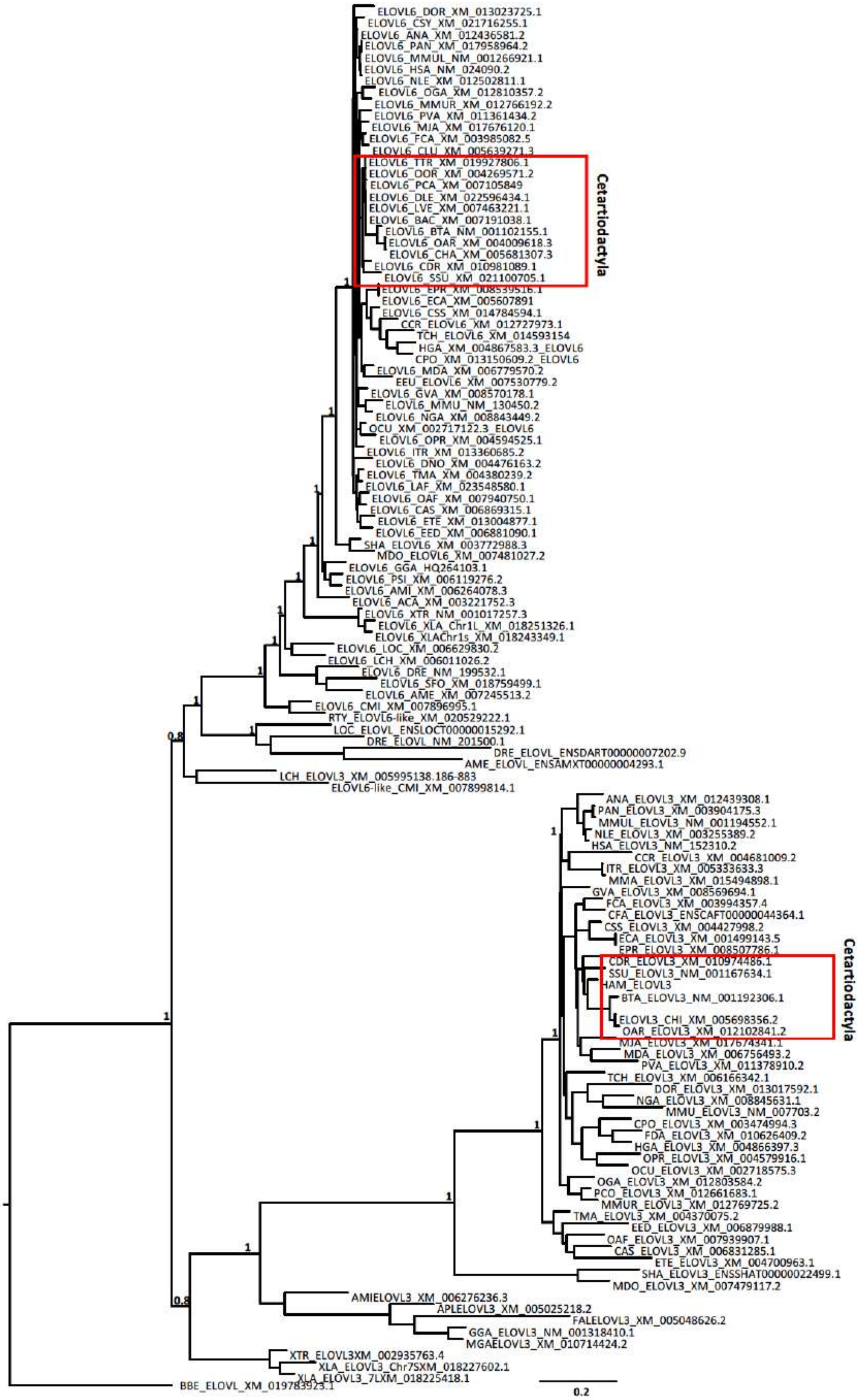

Supplementary Figure S5- Maximum likelihood phylogenetic analysis of ELOVL3 and ELOVL6, branch support calculated using aBayes values at node indicate posterior probabilities and are shown only in the major branches. The analysis comprised 120 taxa, 1058 positions and the best fitting evolutionary model was GTR +G+I. Species acronyms are available in corresponding supplementary table S1. Species acronyms are available in corresponding supplementary table 3.

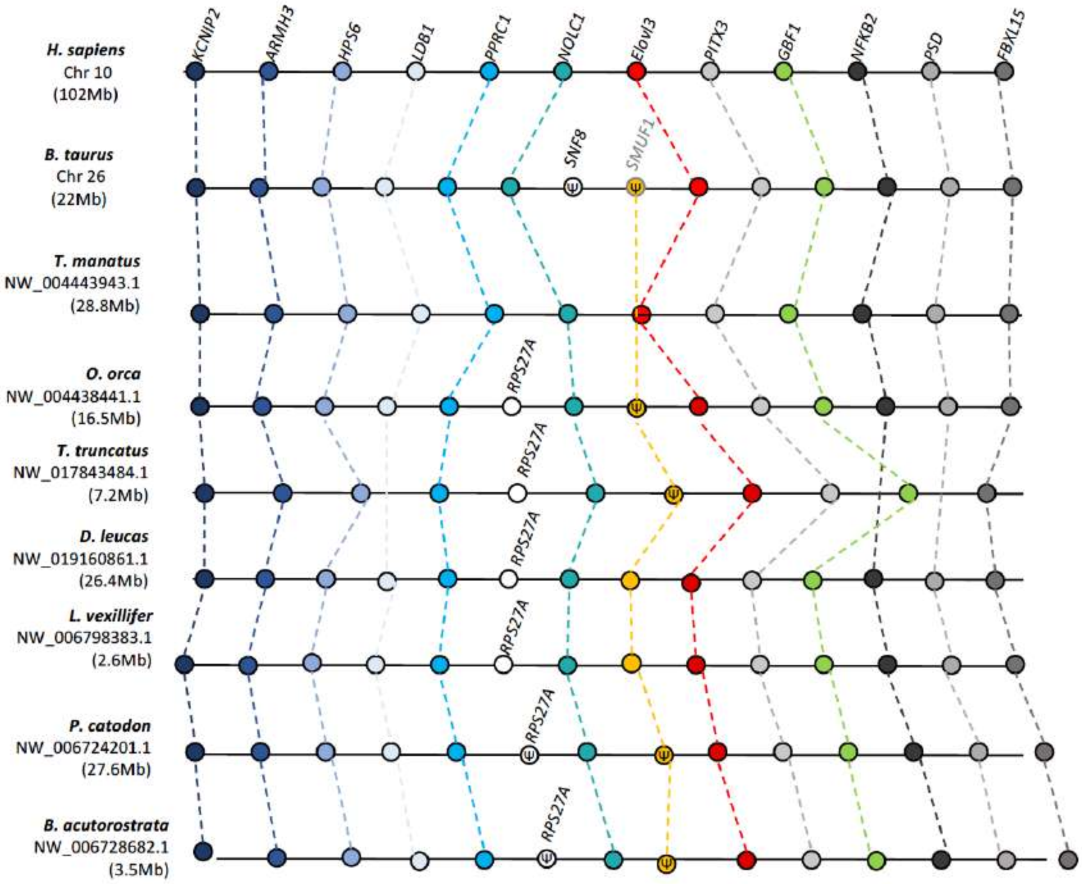

Supplementary Figure S6- Comparative synteny analysis of *Elovl3.*

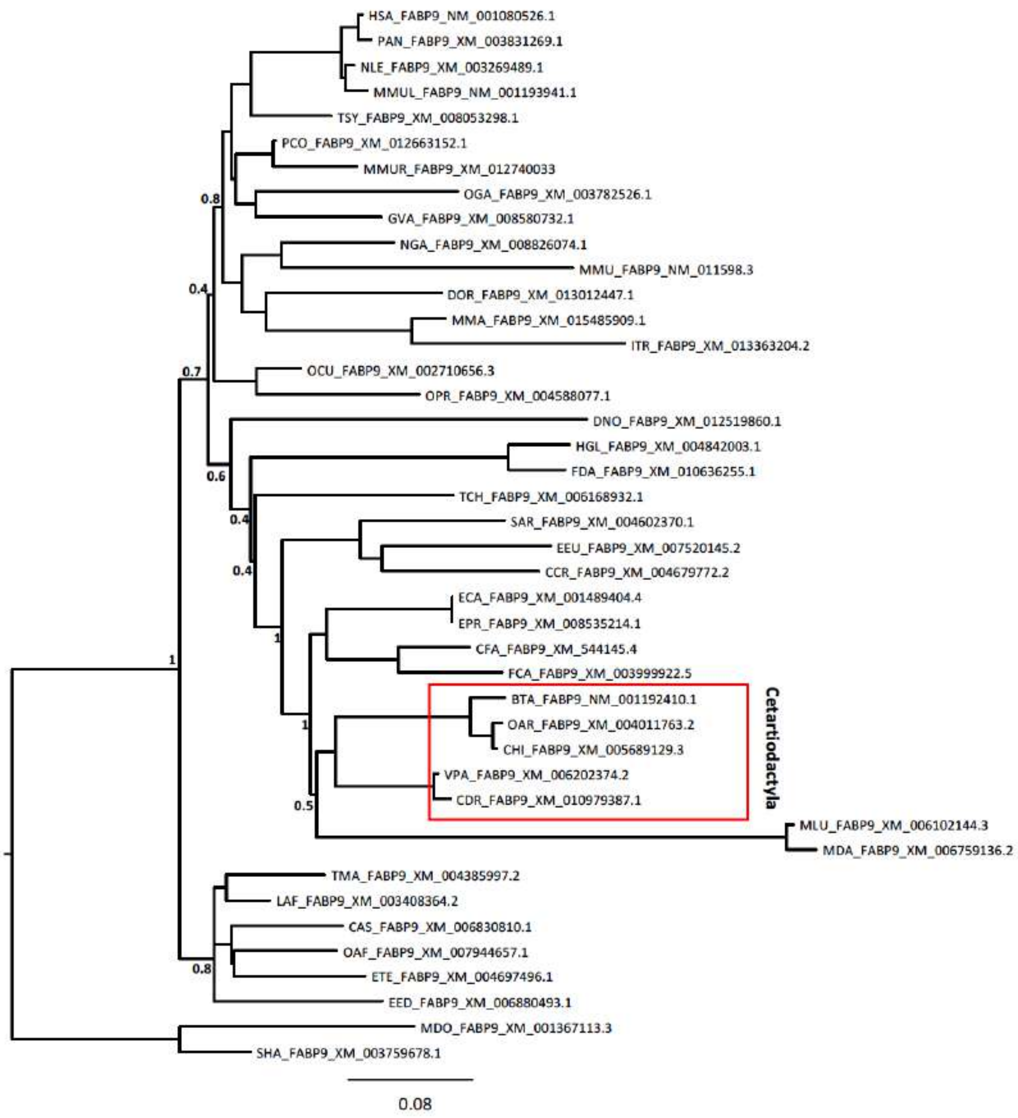

Supplementary Figure S7- Maximum likelihood phylogenetic analysis of FABP9, branch support calculated using aBayes values at node indicate posterior probabilities and are shown only in the major branches. The analysis comprised 42 taxa, 402 positions and the best fitting evolutionary model was GTR +G+I. Species acronyms are available in corresponding supplementary table S1.Species acronyms are available in corresponding supplementary table 4.

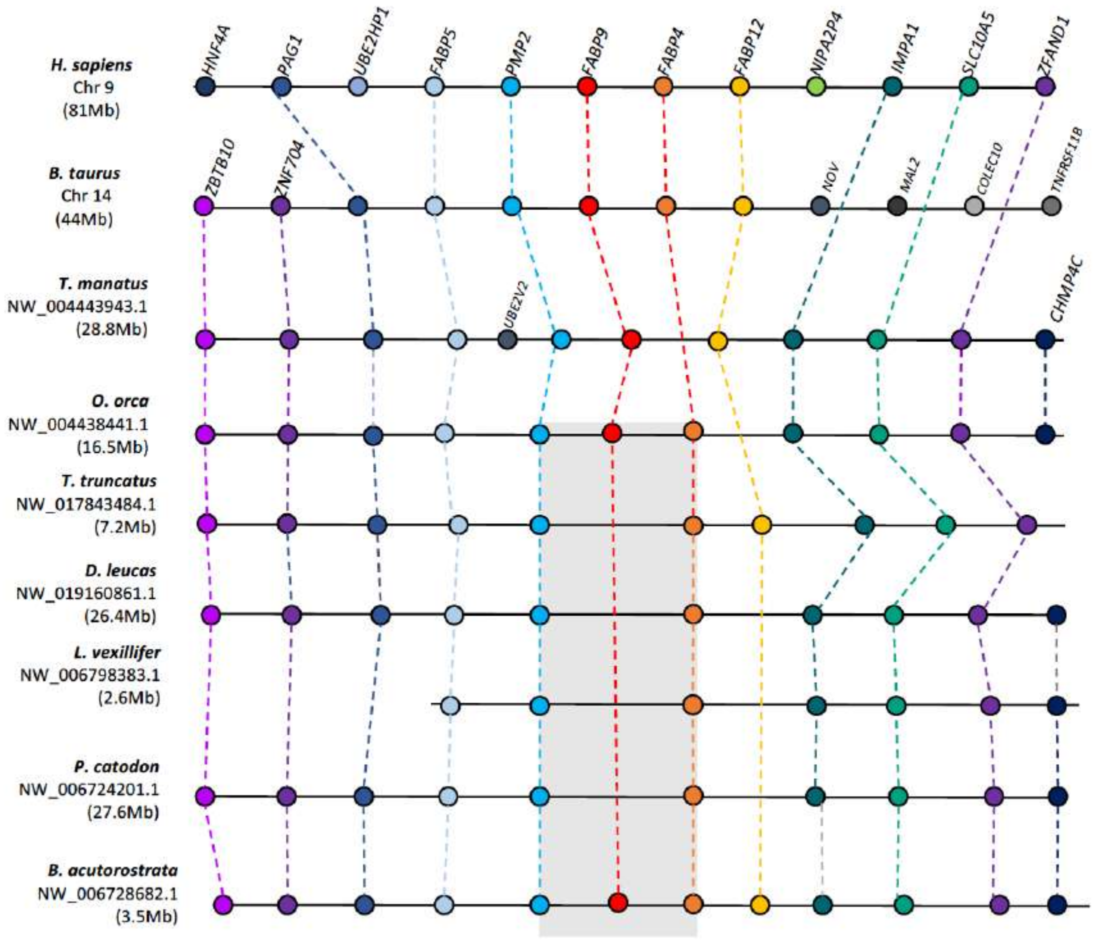

Supplementary Figure S8- Comparative synteny analysis of *FABP9*.
