# Supplementary material for: Complete Inactivation of Sebum-Producing Genes Parallels the Loss of Sebaceous Glands in Cetacea
Source: Mol Biol Evol. 2019 Mar 20;36(6):1270–80. doi: 10.1093/molbev/msz068 (PMC6526905; doi:10.1093/molbev/msz068)

Supplementary Material 2

SRA validation of stop codon in Exon 6 of ***MOGAT3***

Orcinus orca
SRA searched
SRR1164379- University of Durham 2014-02-13 **Sample ID:** SAMN02595096 (AR-Genome)
SRR574977/82- Baylor College of Medicine 2012-09-20 **Sample ID:** SAMN01180276 (AForca1)

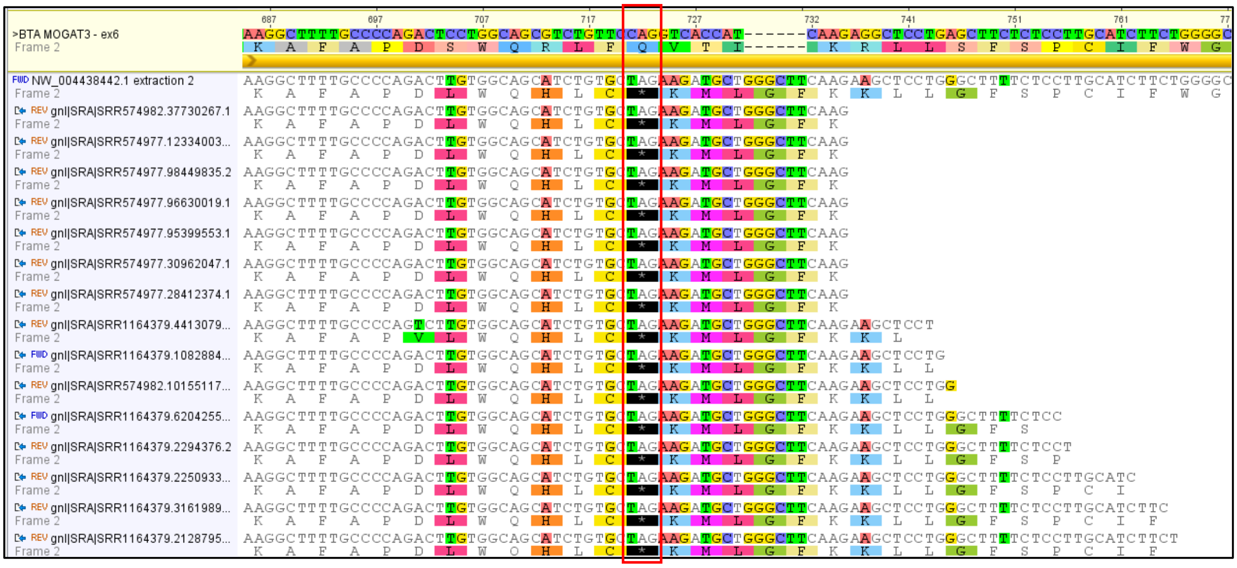

Delphinapterus leucas
SRA searched
SRR5197962- BC Cancer Agency Michael Smith Genome Sciences Centre 2017-06-27 **Sample ID:** SAMN06217832 (Qila21)
SRR5197961- BC Cancer Agency Michael Smith Genome Sciences Centre 2017-06-27 **Sample ID:** SAMN06216270 (Aurora29)

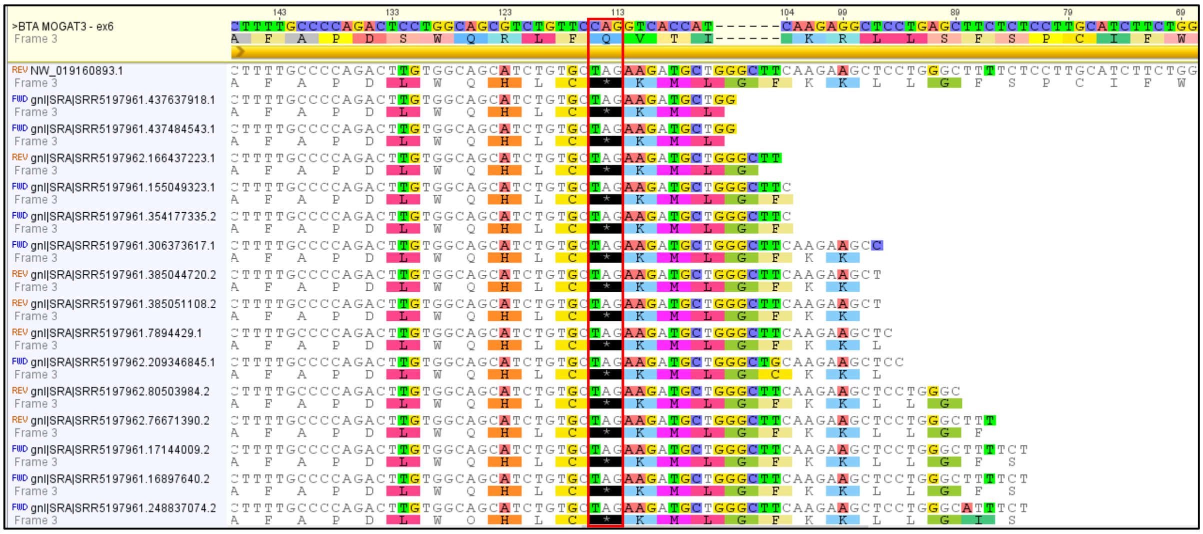

Physeter catodon
SRA searched
SRR5146847- The Genome Center at Washington University School of Medicine in St. Louis 2017-01-05 **Sample ID:** SAMN06187412
SRR5146843- The Genome Center at Washington University School of Medicine in St. Louis 2017-01-05 **Sample ID:** SAMN06187413
SRR5146865- The Genome Center at Washington University School of Medicine in St. Louis 2017-01-05 **Sample ID:** SAMN06187411

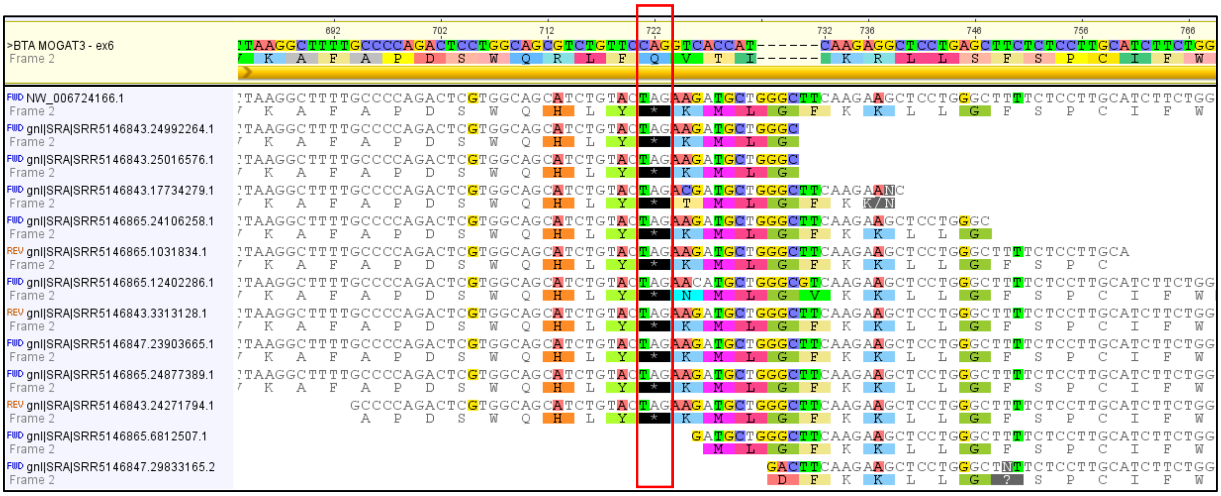

Balaenoptera acutorostrata

SRA searched

SRR924087- Korea Institute of Ocean Science and Technology 2013-10-31 **Sample ID:** SAMN02192644 **(**MinkeWhale-01)

SRR4011112- Institute of Marine Research 2016-08-13 **Sample ID:** SAMN05447714 (AT)

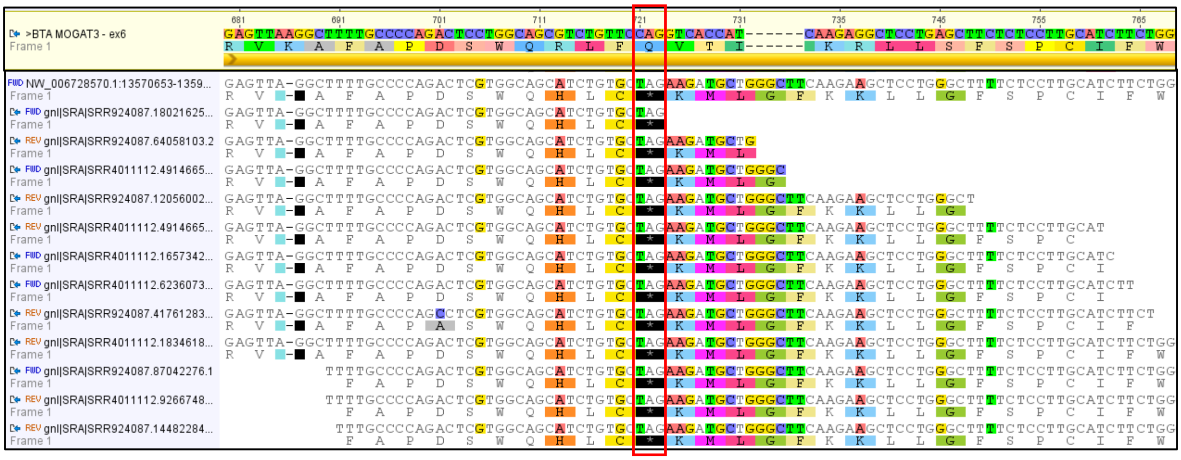

Balaenoptera bonaerensis

SRA searched

SRR4011114- Institute of Marine Research 2016-08-13 **Sample ID:** SAMN05447715

SRR4011113- Institute of Marine Research 2016-08-13 **Sample ID:** SAMN05447715

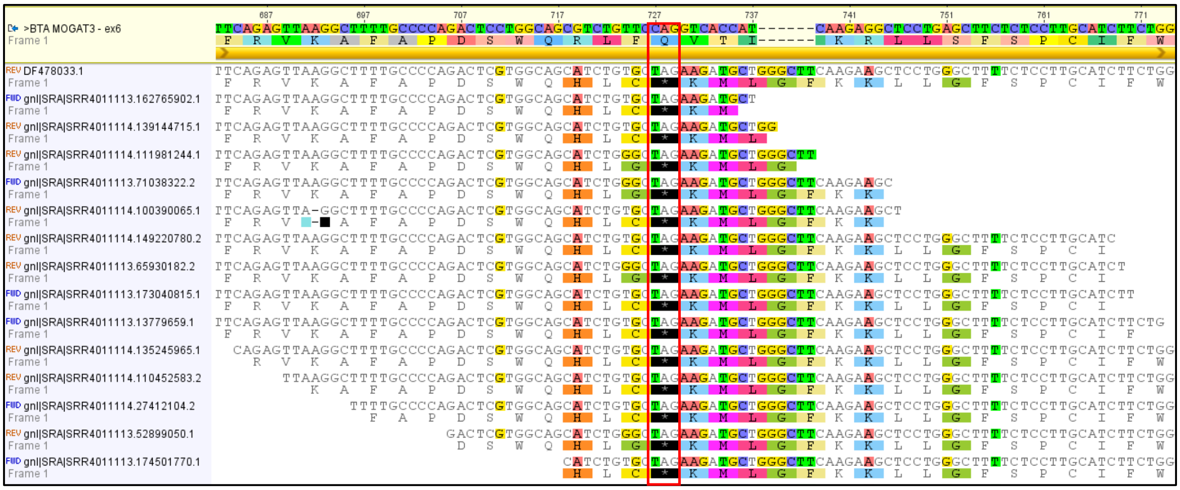

Eschrichtius robustus

SRA searched

SRR5495100- Purdue University 2017-05-02 **Sample ID:** SAMN06837694 (GFD-02)

SRR5495106- Purdue University 2017-05-02 **Sample ID:** SAMN06837692 (ER-14-168)

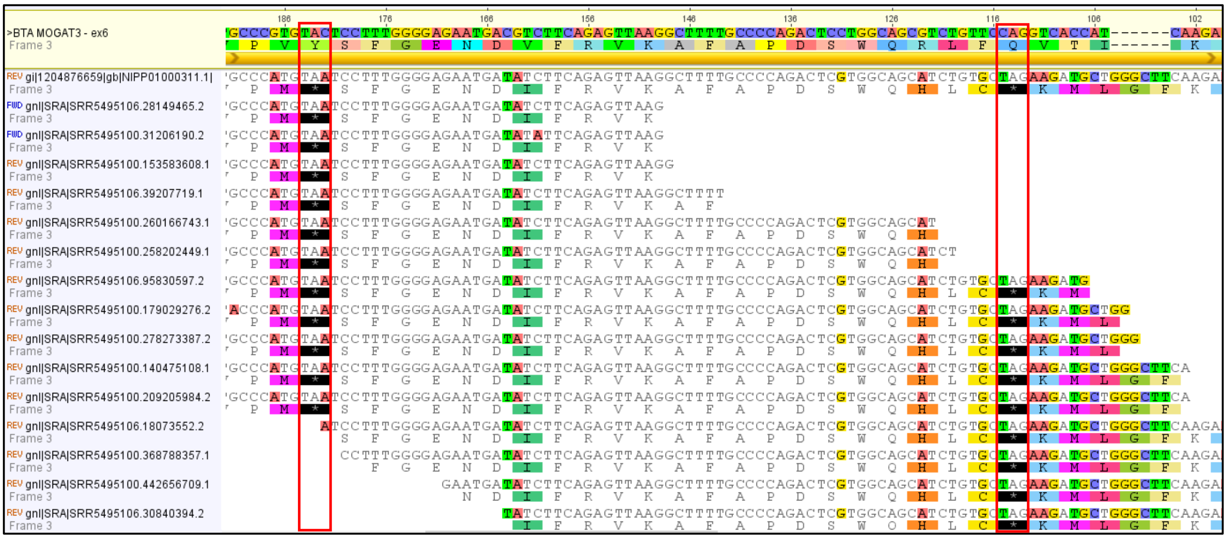

Balaena mysticetus

SRA searched

SRR1685385- University of Liverpool 2015-01-06 **Sample ID:** SAMN03225705 (Bowhead ID325)

SRR1685386- University of Liverpool 2015-01-06 **Sample ID:** SAMN03225705 (Bowhead ID325)

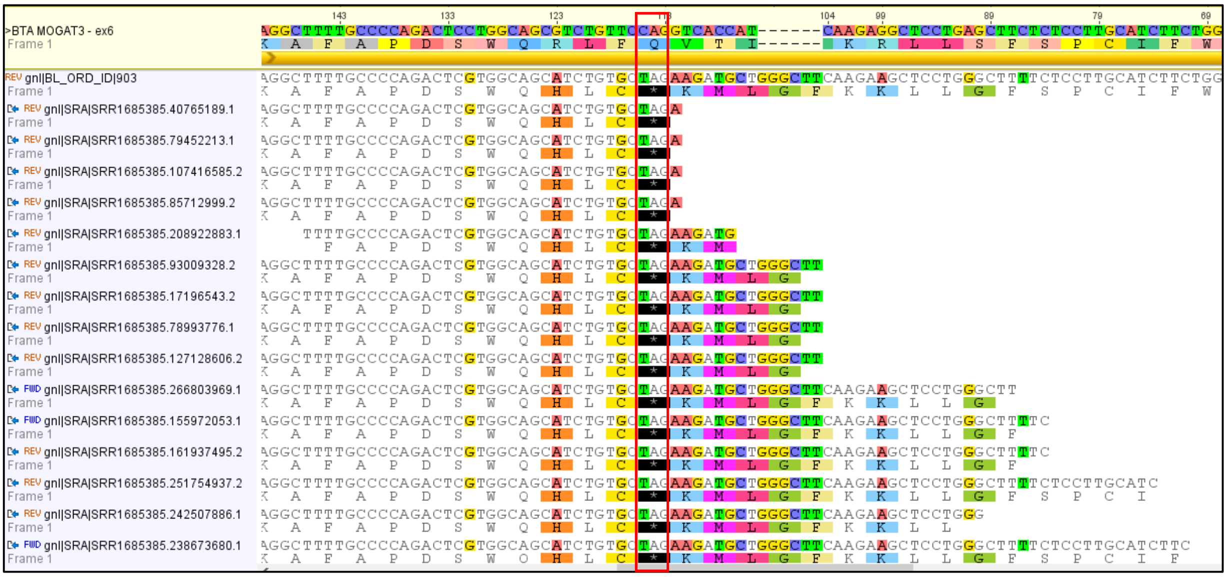

SRA validation of 1 nucleotide deletion in Exon 3 and loss of canonical 3´splice site of ***DGAT2L6***

Orcinus orca
SRA searched
SRR1164379- University of Durham 2014-02-13 **Sample ID:** SAMN02595096 (AR-Genome)
SRR574977/82- Baylor College of Medicine 2012-09-20 **Sample ID:** SAMN01180276 (AForca1)

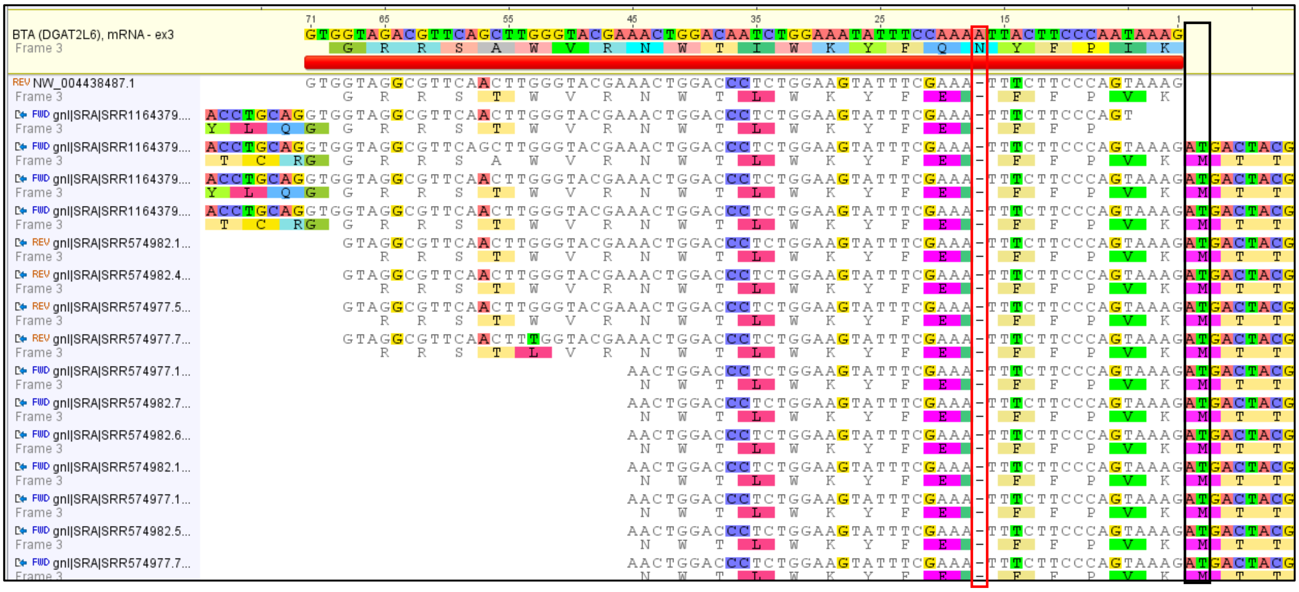

Tursiops truncatus
SRA searched
SRR5125024- National Institute of Standards and Technology 2016-12-27 **Sample ID:** SAMN06114300 (ACU285439A)
SRR606319/20//21- Baylor College of Medicine 2015-07-22 **Sample ID:** SAMN00000070
SRR2148845 - Beijing Genome Institute 2016-08-10 **Sample ID**: SAMN03968479

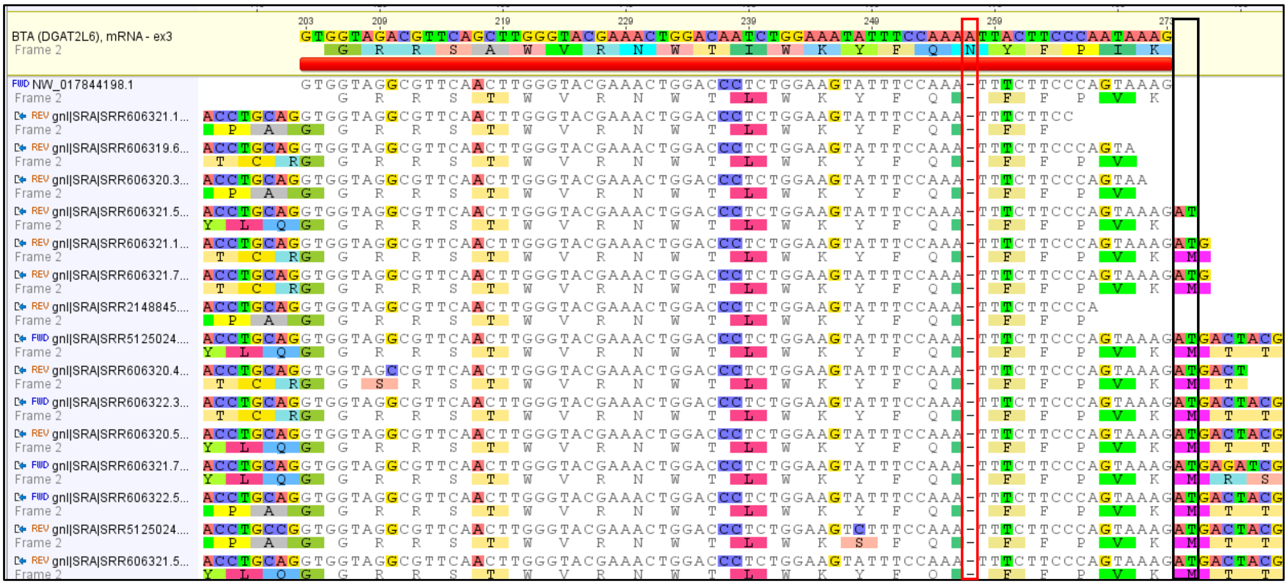

Delphinapterus leucas

SRA searched

SRR5197962- BC Cancer Agency Michael Smith Genome Sciences Centre 2017-06-27 **Sample ID:** SAMN06217832 (Qila21)

SRR5197961- BC Cancer Agency Michael Smith Genome Sciences Centre 2017-06-27 **Sample ID:** SAMN06216270 (Aurora29)

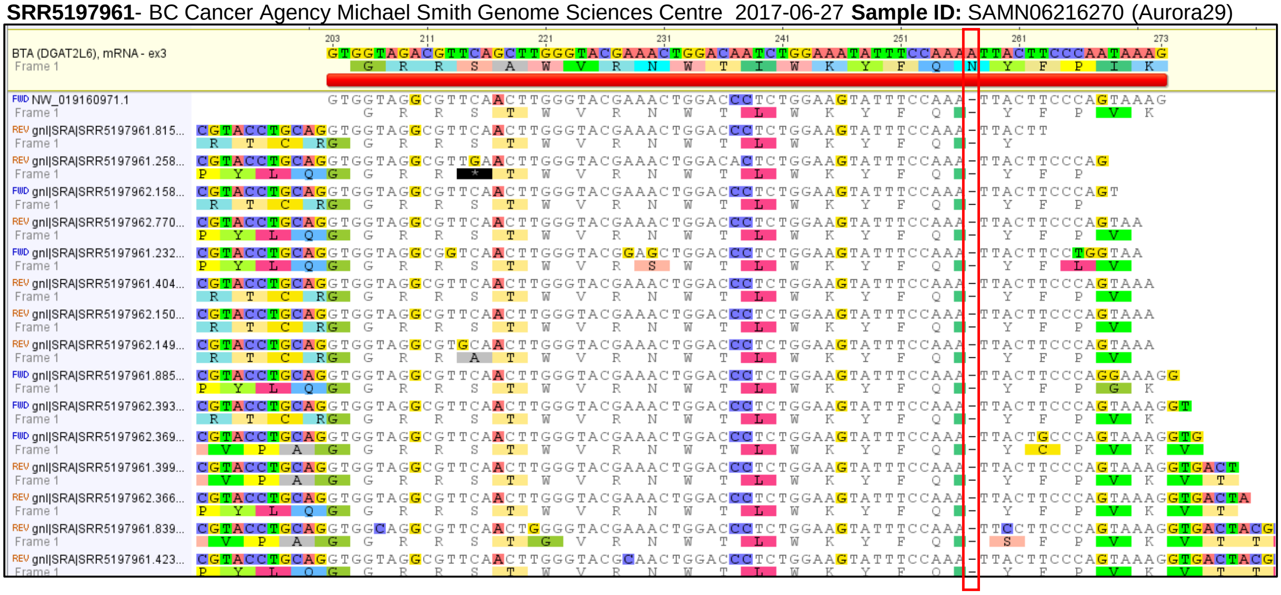

Physeter catodon

SRA searched

SRR5146843- The Genome Center at Washington University School of Medicine in St. Louis 2017-01-05 **Sample ID:** SAMN06187413

SRR5146865- The Genome Center at Washington University School of Medicine in St. Louis 2017-01-05 **Sample ID:** SAMN06187411

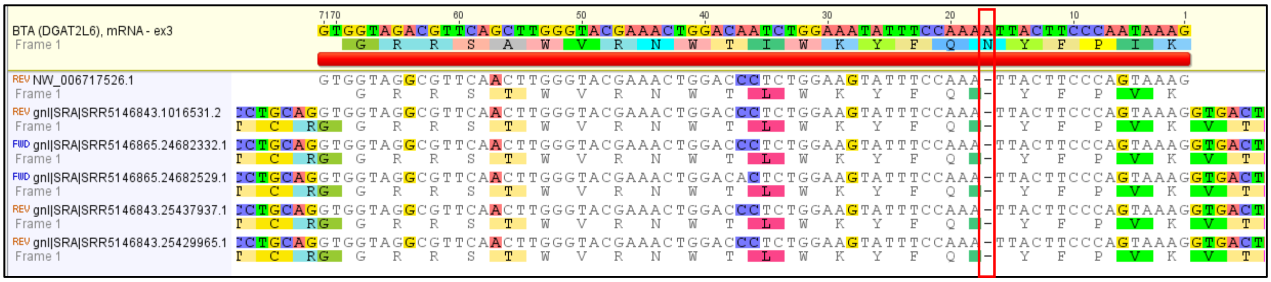

Balaenoptera acutorostrata

SRA searched

SRR924087- Korea Institute of Ocean Science and Technology 2013-10-31 **Sample ID:** SAMN02192644 **(**MinkeWhale-01)

SRR4011112- Institute of Marine Research 2016-08-13 **Sample ID:** SAMN05447714 (AT)

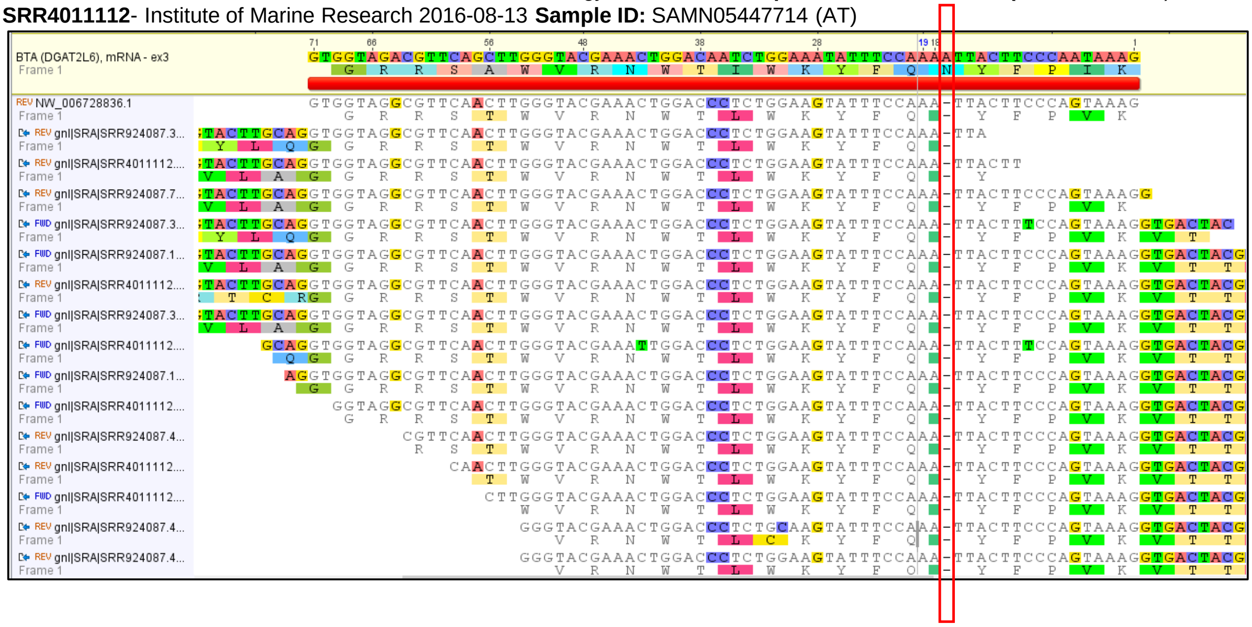

Balaenoptera bonaerensis

SRA searched

SRR4011114- Institute of Marine Research 2016-08-13 **Sample ID:** SAMN05447715

SRR4011113- Institute of Marine Research 2016-08-13 **Sample ID:** SAMN05447715

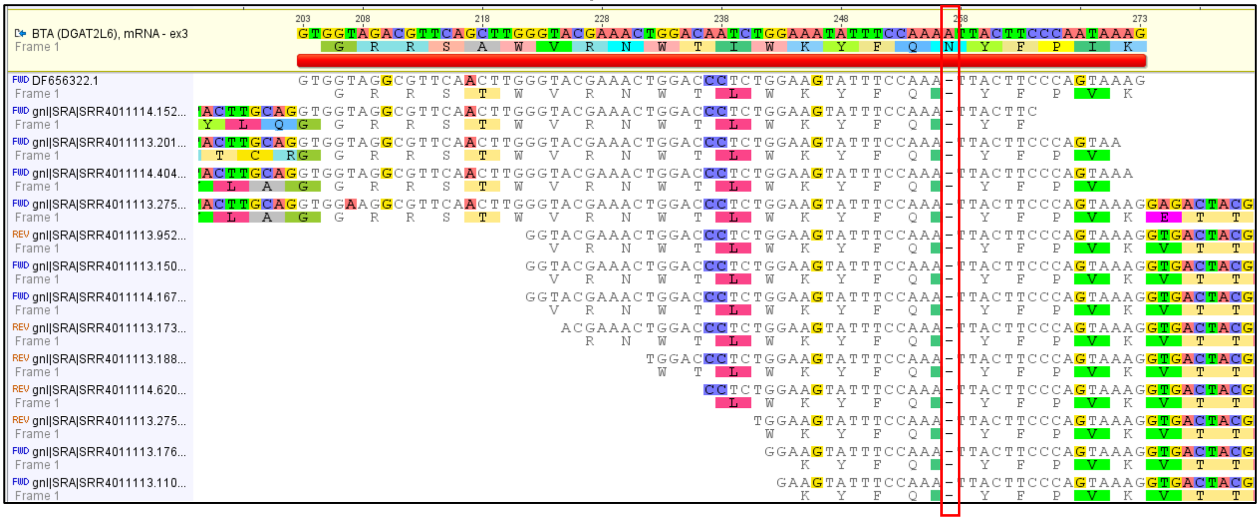

Eschrichtius robustus
SRA searched
SRR5495100- Purdue University 2017-05-02 **Sample ID:** SAMN06837694 (GFD-02)
SRR5495106- Purdue University 2017-05-02 **Sample ID:** SAMN06837692 (ER-14-168)

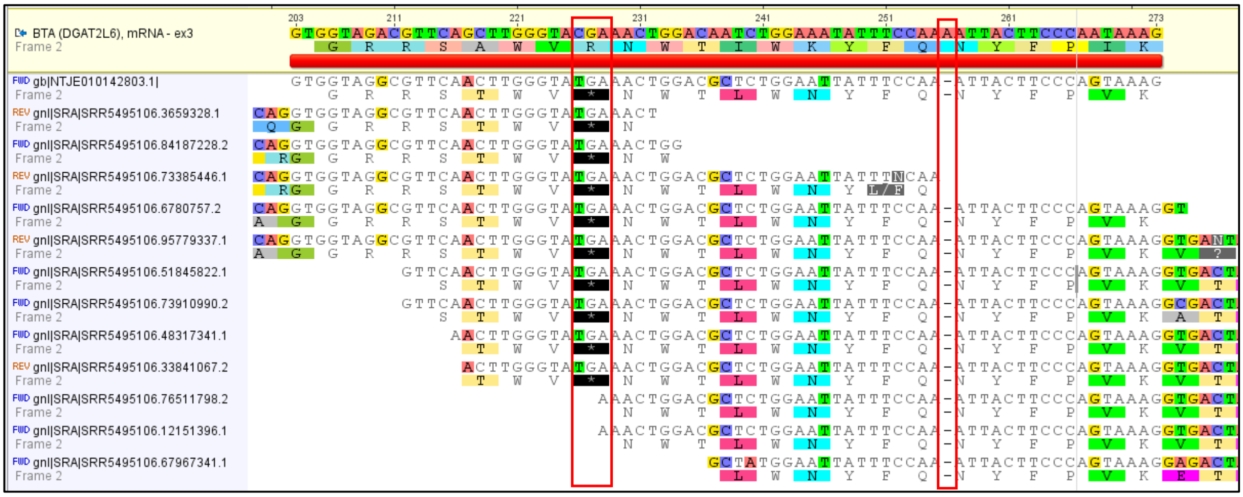

Balaena mysticetus
SRA searched
SRR1685385- University of Liverpool 2015-01-06 **Sample ID:** SAMN03225705 (Bowhead ID325)
SRR1685386- University of Liverpool 2015-01-06 **Sample ID:** SAMN03225705 (Bowhead ID325)

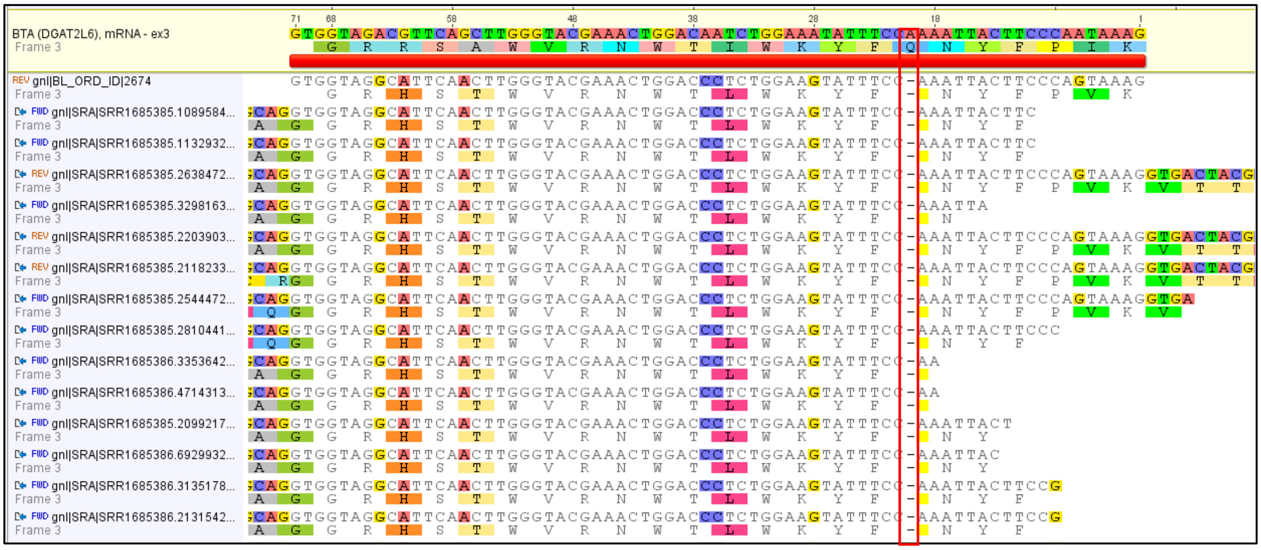

Supplementary Material 2

SRA validation of stop codon in Exon 2 of ***AWAT1***

Orcinus orca
SRA searched
SRR1164379- University of Durham 2014-02-13 **Sample ID:** SAMN02595096 (AR-Genome)
SRR574977- Baylor College of Medicine 2012-09-20 **Sample ID:** SAMN01180276 (AForca1)

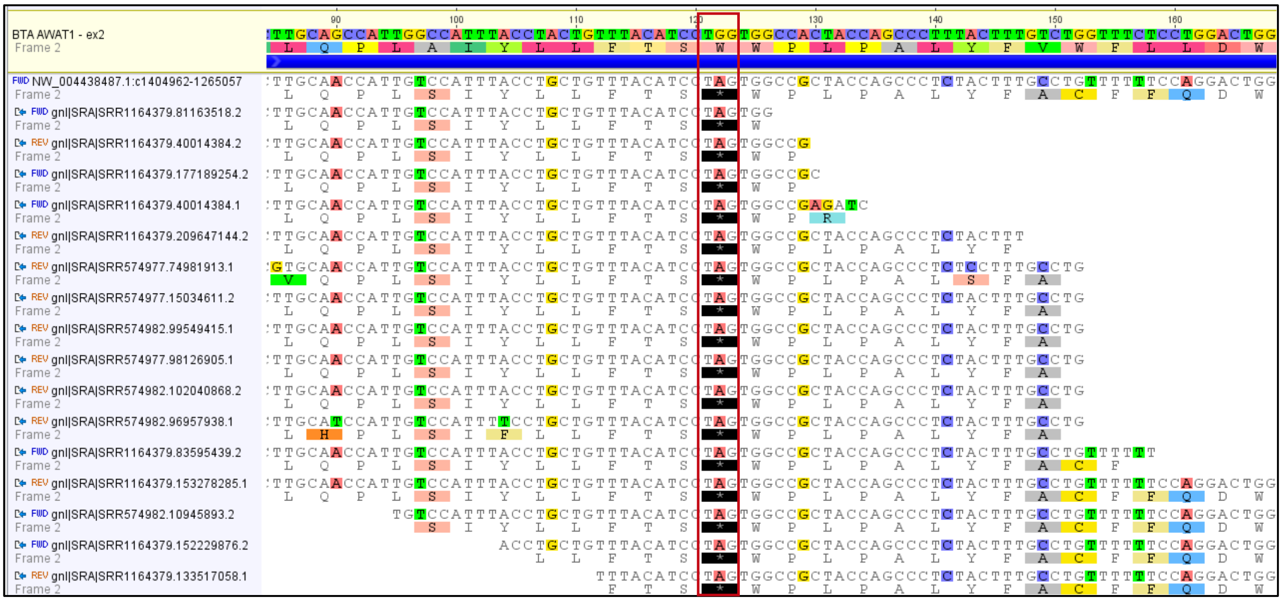

Tursiops truncatus
SRA searched
SRR5125024- National Institute of Standards and Technology 2016-12-27 **Sample ID:** SAMN06114300 (ACU285439A)
SRR606320/22- Baylor College of Medicine 2015-07-22 **Sample ID:** SAMN00000070
SRR2148845 - Beijing Genome Institute 2016-08-10 **Sample ID**: SAMN03968479

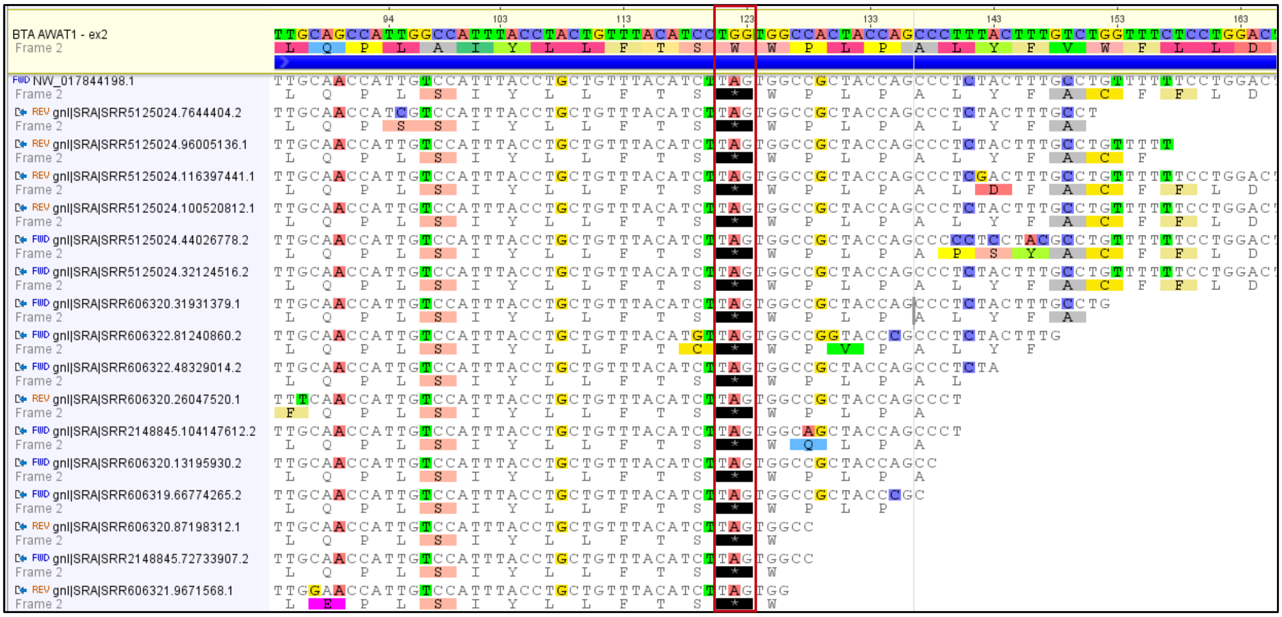

Delphinapterus leucas
SRA searched
SRR5197962- BC Cancer Agency Michael Smith Genome Sciences Centre 2017-06-27 **Sample ID:** SAMN06217832 (Qila21)
SRR5197961- BC Cancer Agency Michael Smith Genome Sciences Centre 2017-06-27 **Sample ID:** SAMN06216270 (Aurora29)

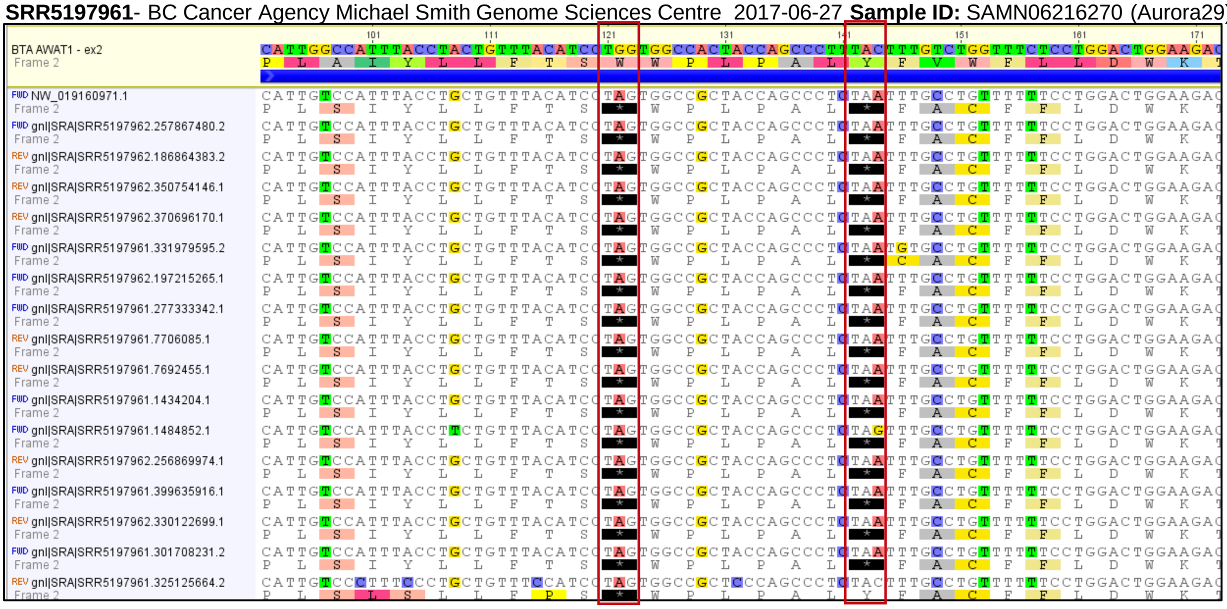

Physeter catodon
SRA searched
SRR5146847- The Genome Center at Washington University School of Medicine in St. Louis 2017-01-05 **Sample ID:** SAMN06187412
SRR5146843- The Genome Center at Washington University School of Medicine in St. Louis 2017-01-05 **Sample ID:** SAMN06187413
SRR5146865- The Genome Center at Washington University School of Medicine in St. Louis 2017-01-05 **Sample ID:** SAMN06187411

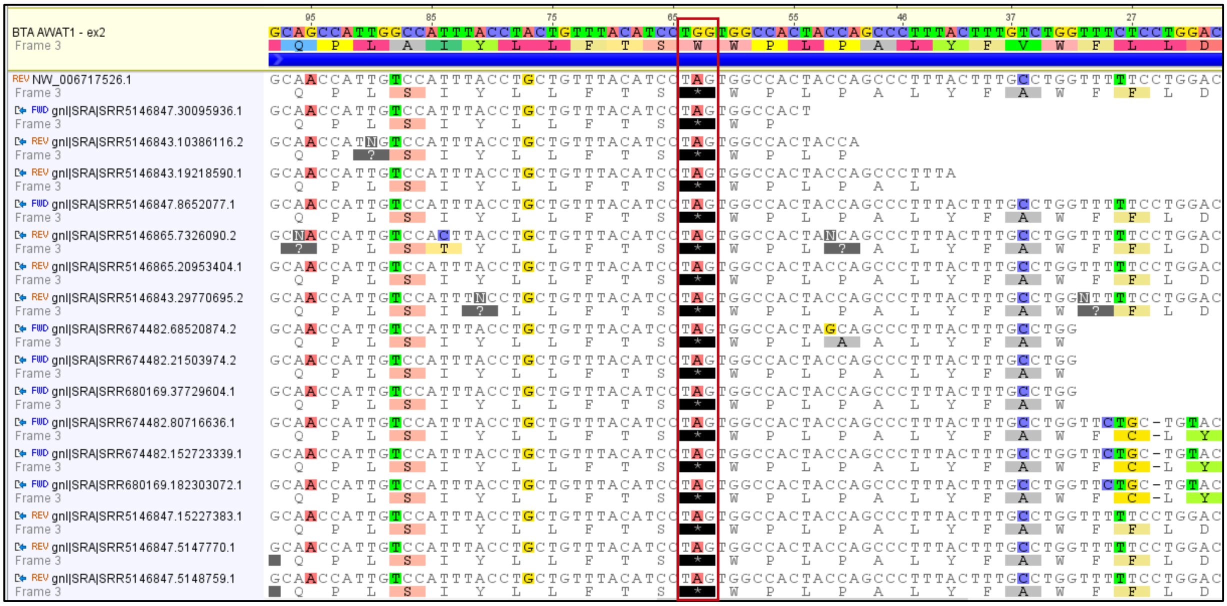

Balaenoptera acutorostrata

SRA searched

SRR924087- Korea Institute of Ocean Science and Technology 2013-10-31 **Sample ID:** SAMN02192644 **(**MinkeWhale-01)

SRR4011112- Institute of Marine Research 2016-08-13 **Sample ID:** SAMN05447714 (AT)

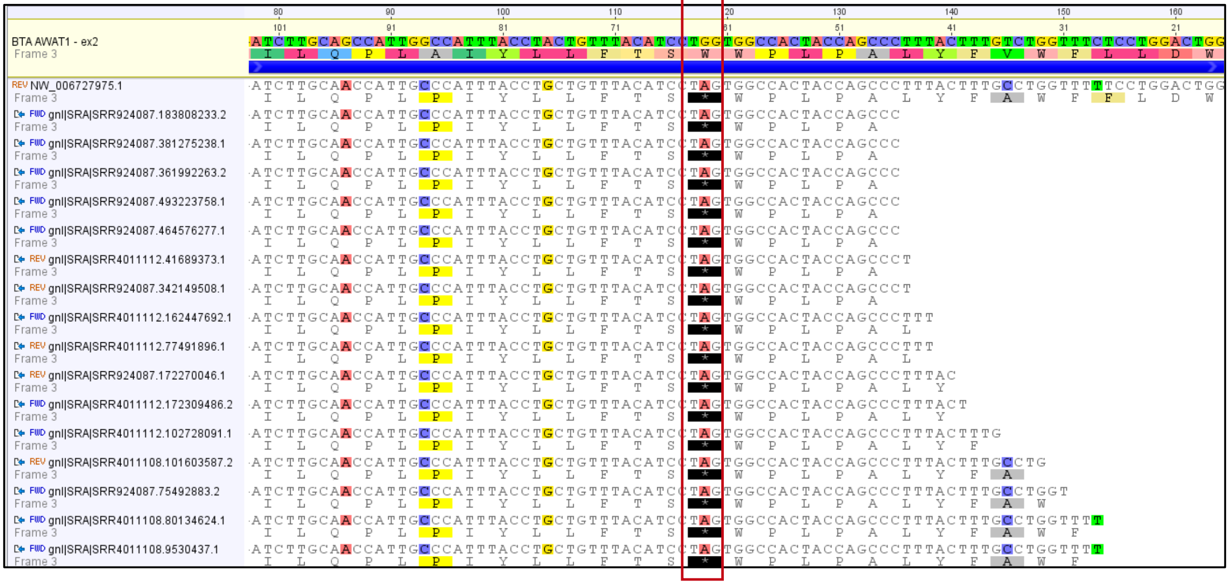

Balaenoptera bonaerensis

SRA searched

SRR4011114- Institute of Marine Research 2016-08-13 **Sample ID:** SAMN05447715

SRR4011113- Institute of Marine Research 2016-08-13 **Sample ID:** SAMN05447715

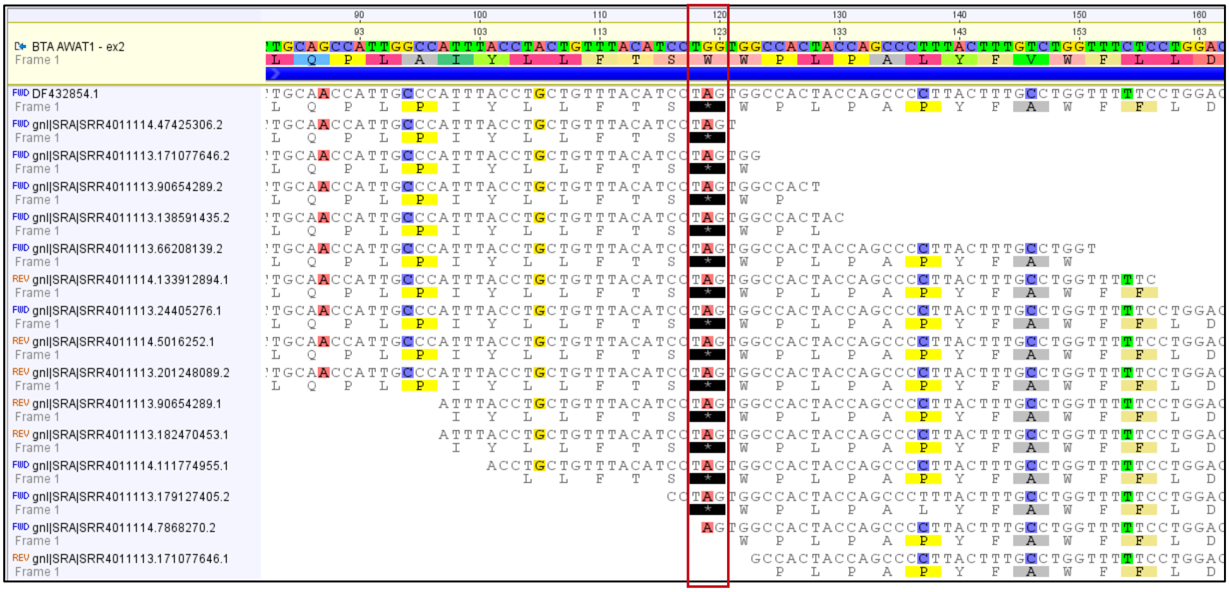

Eschrichtius robustus
SRA searched
SRR5495100- Purdue University 2017-05-02 **Sample ID:** SAMN06837694 (GFD-02)
SRR5495106- Purdue University 2017-05-02 **Sample ID:** SAMN06837692 (ER-14-168)

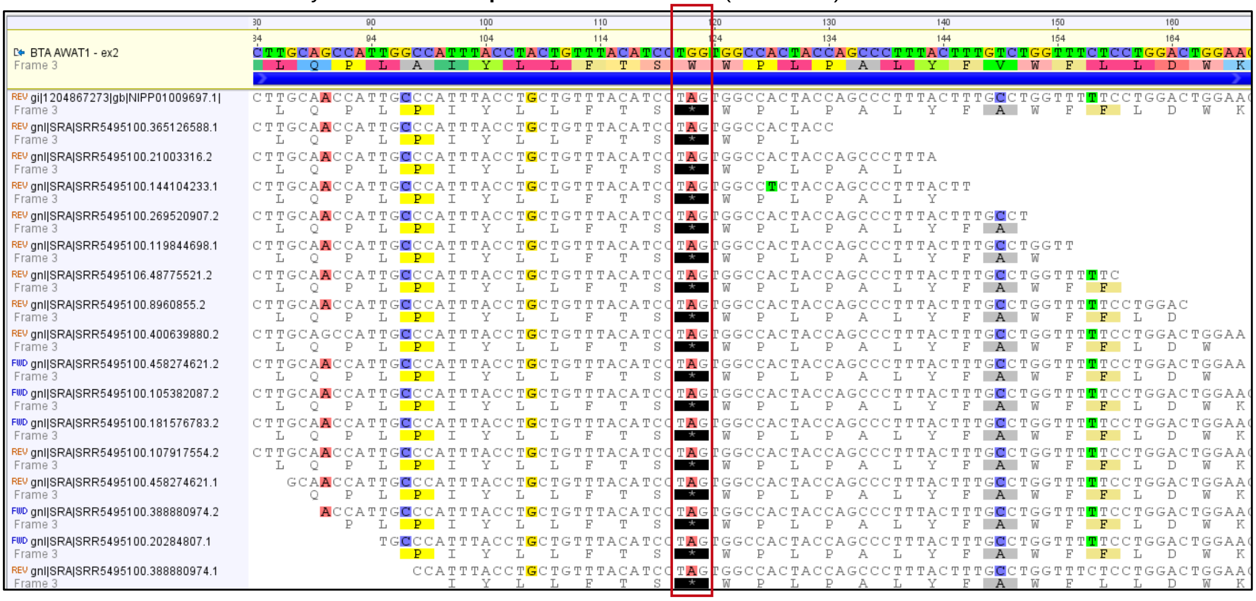

Balaena mysticetus
SRA searched
SRR1685385- University of Liverpool 2015-01-06 **Sample ID:** SAMN03225705 (Bowhead ID325)
SRR1685386- University of Liverpool 2015-01-06 **Sample ID:** SAMN03225705 (Bowhead ID325)

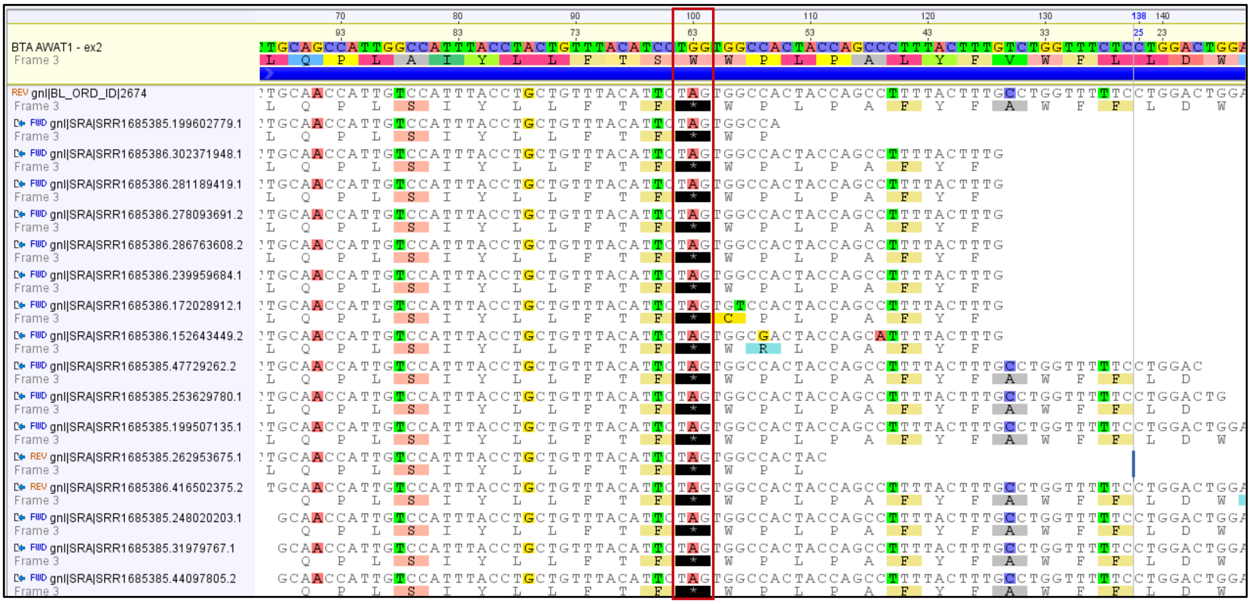

SRA validation of inactivating mutations in ***AWAT2***

Orcinus orca 1 nt deletion in ex6
SRA searched
SRR1164379- University of Durham 2014-02-13 **Sample ID:** SAMN02595096 (AR-Genome)
SRR574977- Baylor College of Medicine 2012-09-20 **Sample ID:** SAMN01180276 (AForca1)

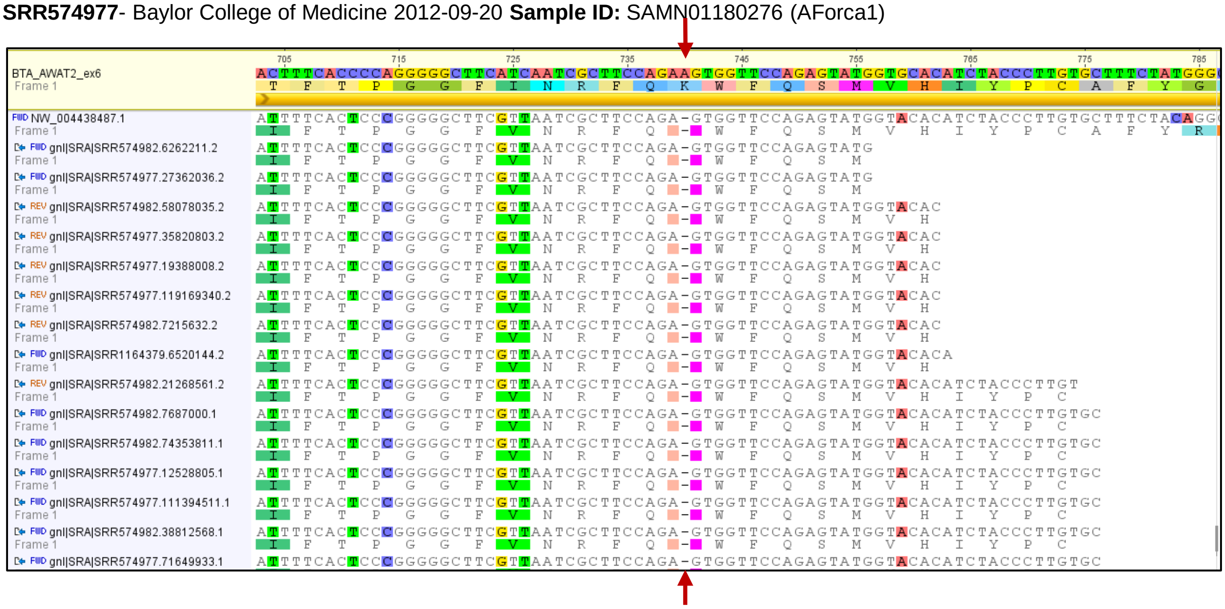

Tursiops truncatus
SRA searched
SRR5125024- National Institute of Standards and Technology 2016-12-27 **Sample ID:** SAMN06114300 (ACU285439A)
SRR606320/21//22- Baylor College of Medicine 2015-07-22 **Sample ID:** SAMN00000070
SRR2148845 - Beijing Genome Institute 2016-08-10 **Sample ID**: SAMN03968479

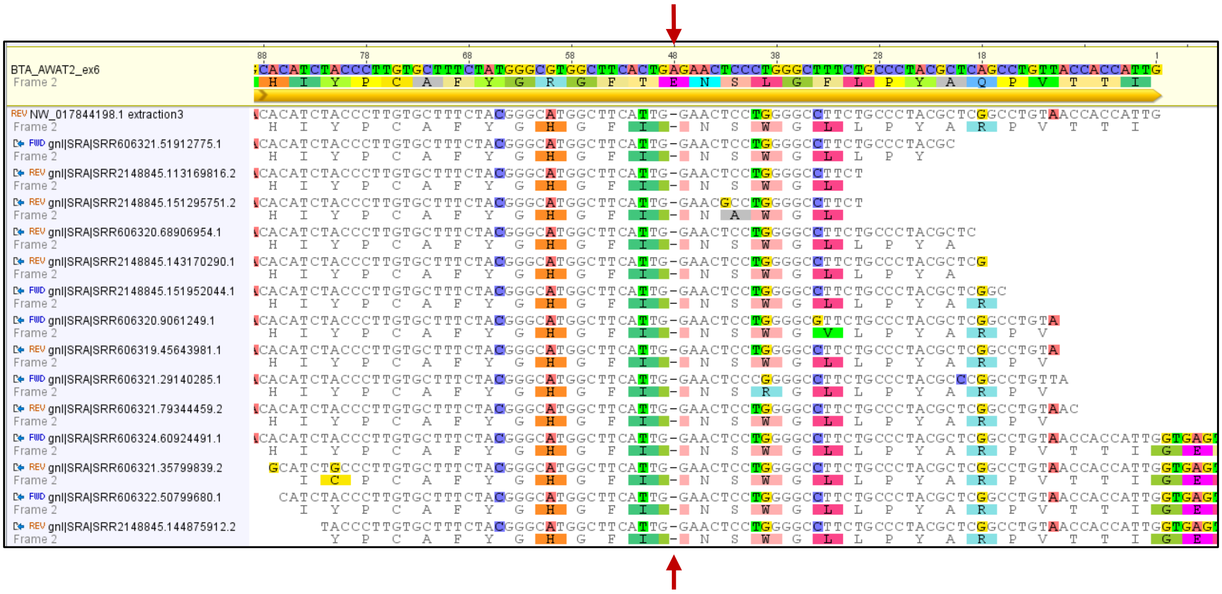

Delphinapterus leucas
SRA searched
SRR5197962- BC Cancer Agency Michael Smith Genome Sciences Centre 2017-06-27 **Sample ID:** SAMN06217832 (Qila21)
SRR5197961- BC Cancer Agency Michael Smith Genome Sciences Centre 2017-06-27 **Sample ID:** SAMN06216270 (Aurora29)

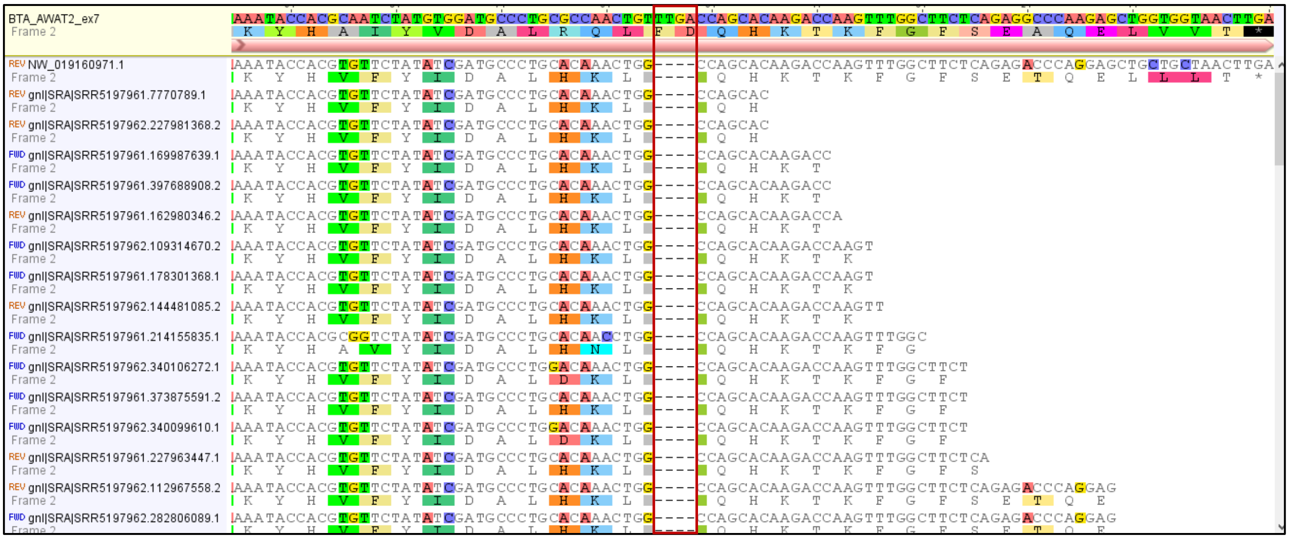

Physeter catodon
SRA searched
SRR5146847- The Genome Center at Washington University School of Medicine in St. Louis 2017-01-05 **Sample ID:** SAMN06187412
SRR5146843- The Genome Center at Washington University School of Medicine in St. Louis 2017-01-05 **Sample ID:** SAMN06187413
SRR5146865- The Genome Center at Washington University School of Medicine in St. Louis 2017-01-05 **Sample ID:** SAMN06187411

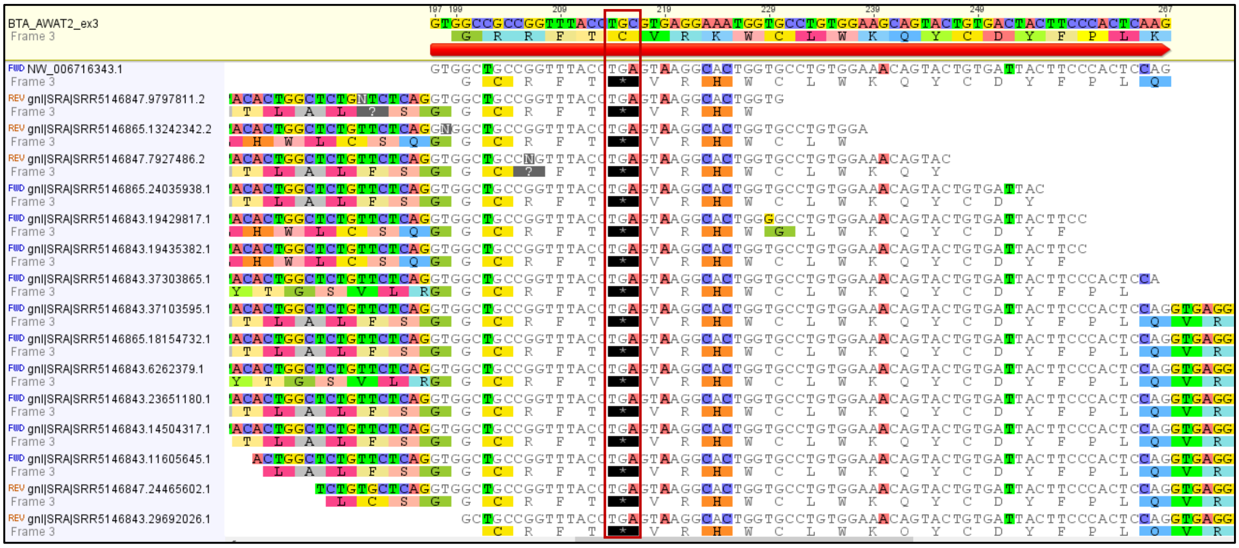

Balaenoptera acutorostrata

SRA searched

SRR924087- Korea Institute of Ocean Science and Technology 2013-10-31 **Sample ID:** SAMN02192644 **(**MinkeWhale-01)

SRR4011112- Institute of Marine Research 2016-08-13 **Sample ID:** SAMN05447714 (AT)

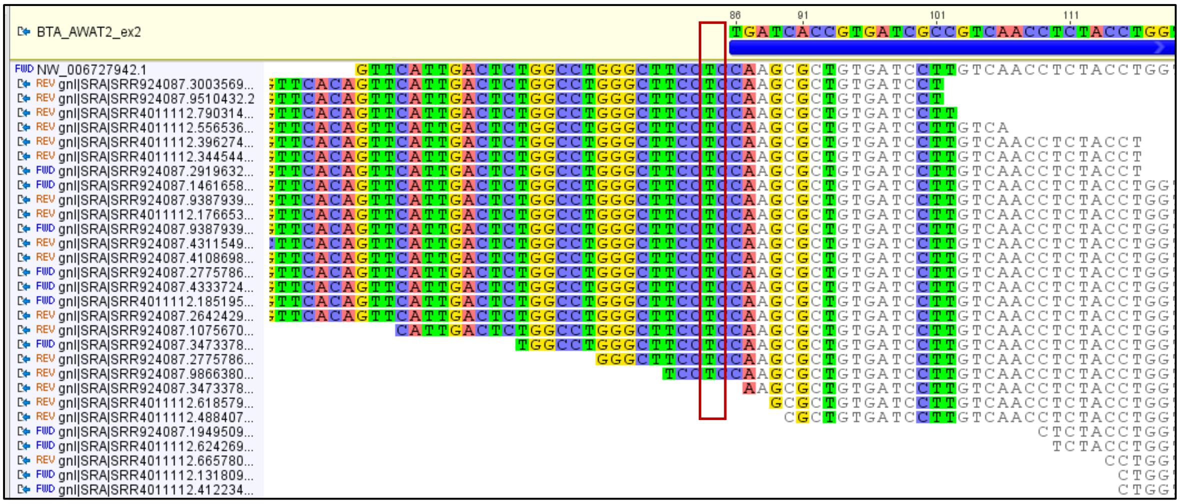

Balaenoptera bonaerensis

SRA searched

SRR4011114- Institute of Marine Research 2016-08-13 **Sample ID:** SAMN05447715

SRR4011113- Institute of Marine Research 2016-08-13 **Sample ID:** SAMN05447715

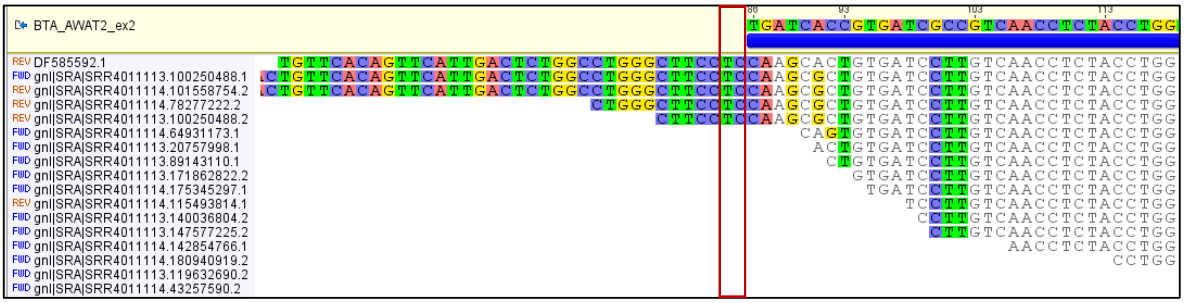

Eschrichtius robustus

SRA searched

SRR5495100- Purdue University 2017-05-02 **Sample ID:** SAMN06837694 (GFD-02)

SRR5495106- Purdue University 2017-05-02 **Sample ID:** SAMN06837692 (ER-14-168)

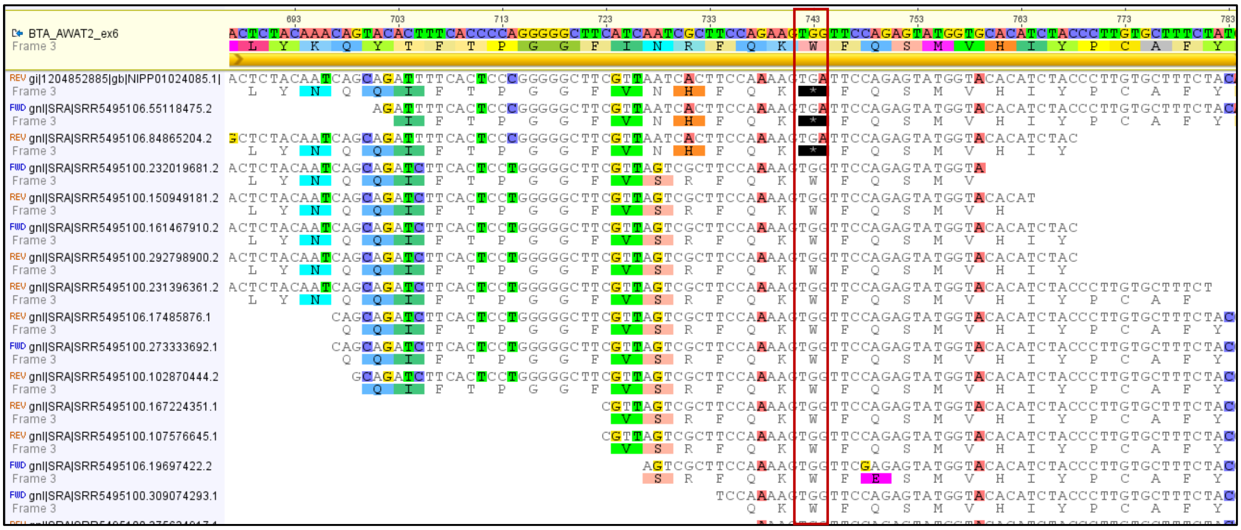

Supplementary Material 2

SRA validation of stop codon in Exon 3 of ***ELOVL3***

Orcinus orca
SRA searched
SRR1164379- University of Durham 2014-02-13 **Sample ID:** SAMN02595096 (AR-Genome)
SRR574977- Baylor College of Medicine 2012-09-20 **Sample ID:** SAMN01180276 (AForca1)

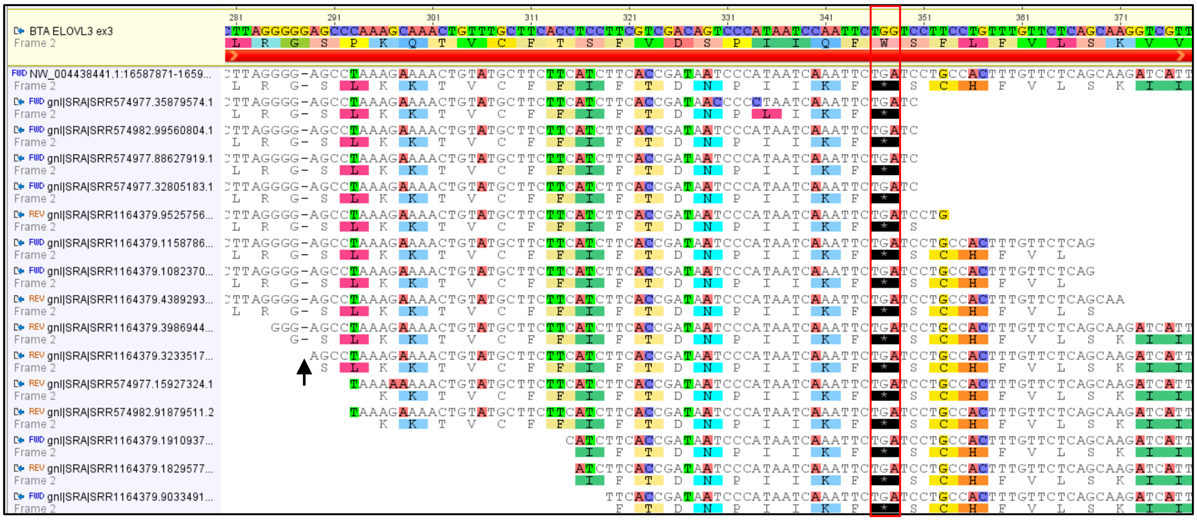

Tursiops truncatus
SRA searched
SRR5125024- National Institute of Standards and Technology 2016-12-27 **Sample ID:** SAMN06114300 (ACU285439A)
SRR606320/21/22/24- Baylor College of Medicine 2015-07-22 **Sample ID:** SAMN00000070
SRR2148845 - Beijing Genome Institute 2016-08-10 **Sample ID**: SAMN03968479

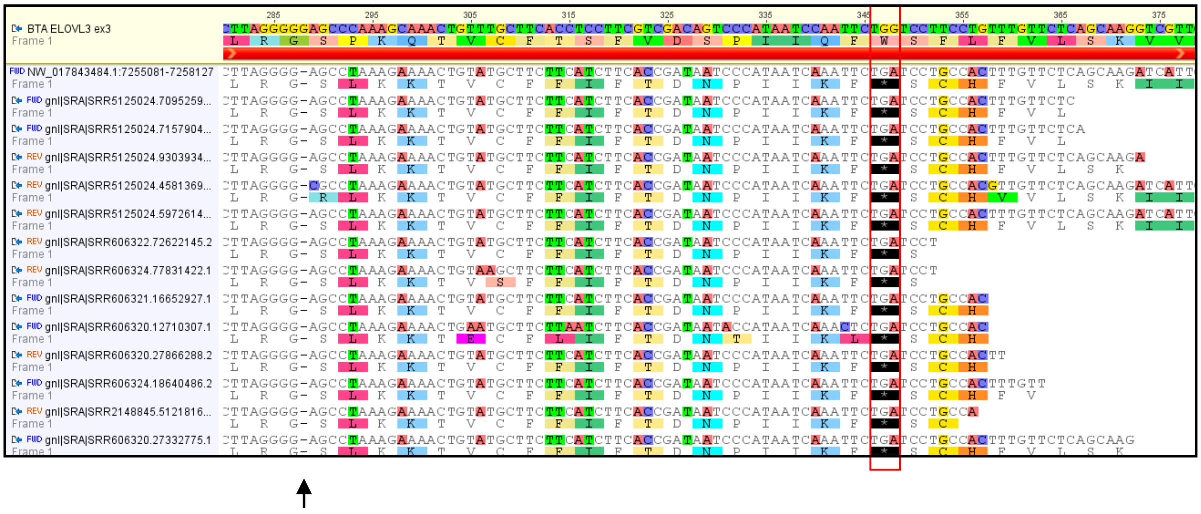

Delphinapterus leucas

SRA searched

SRR5197962- BC Cancer Agency Michael Smith Genome Sciences Centre 2017-06-27 **Sample ID:** SAMN06217832 (Qila21)

SRR5197961- BC Cancer Agency Michael Smith Genome Sciences Centre 2017-06-27 **Sample ID:** SAMN06216270 (Aurora29)

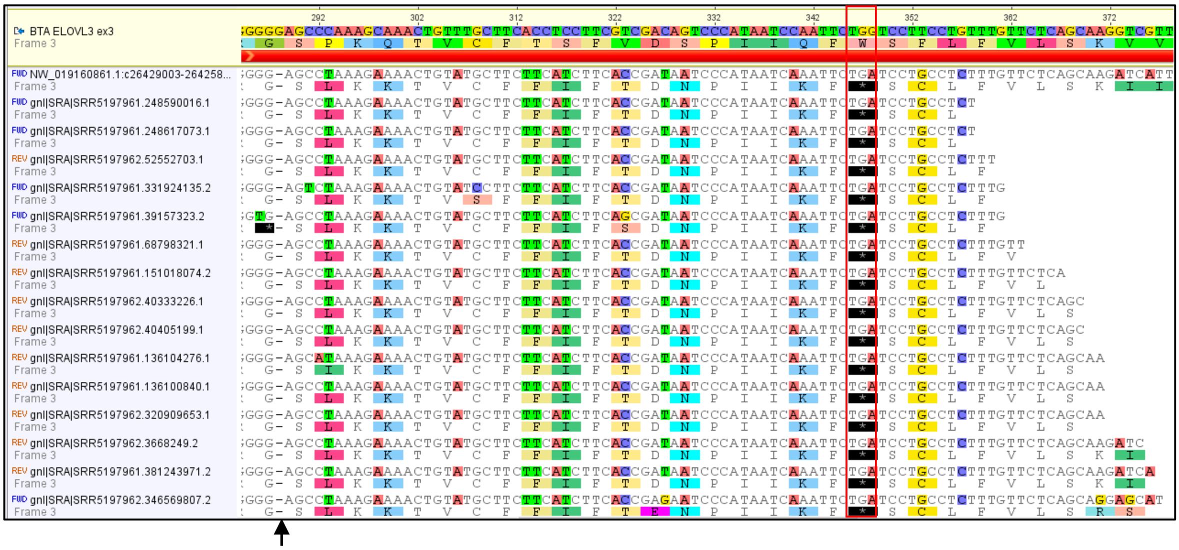

Physeter catodon

SRA searched

SRR5146847- The Genome Center at Washington University School of Medicine in St. Louis 2017-01-05 **Sample ID:** SAMN06187412

SRR5146843- The Genome Center at Washington University School of Medicine in St. Louis 2017-01-05 **Sample ID:** SAMN06187413

SRR5146865- The Genome Center at Washington University School of Medicine in St. Louis 2017-01-05 **Sample ID:** SAMN06187411

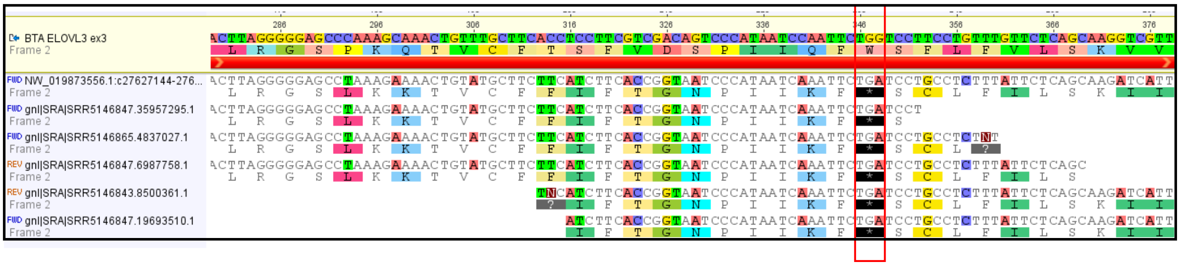

Balaenoptera acutorostrata

SRA searched

SRR924087- Korea Institute of Ocean Science and Technology 2013-10-31 **Sample ID:** SAMN02192644 **(**MinkeWhale-01)

SRR4011112- Institute of Marine Research 2016-08-13 **Sample ID:** SAMN05447714 (AT)

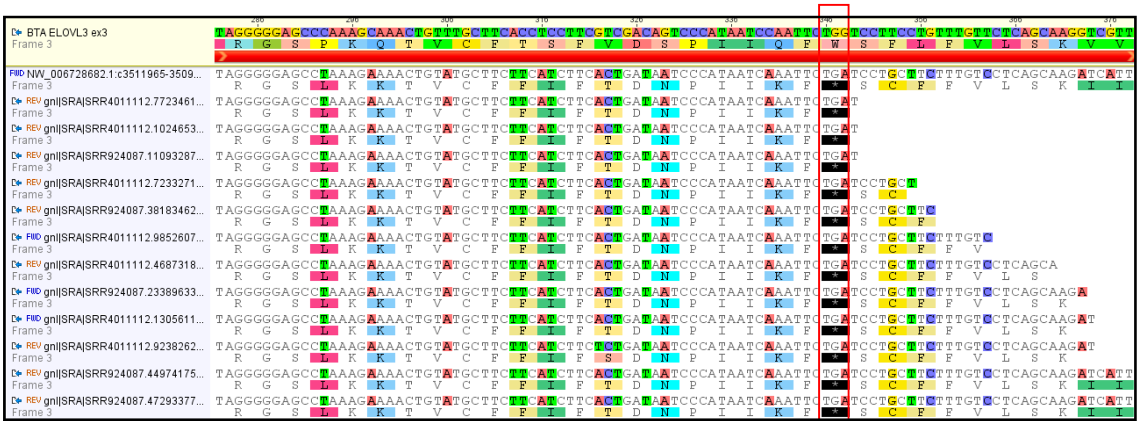

Balaenoptera bonaerensis

SRA searched

SRR4011114- Institute of Marine Research 2016-08-13 **Sample ID:** SAMN05447715

SRR4011113- Institute of Marine Research 2016-08-13 **Sample ID:** SAMN05447715

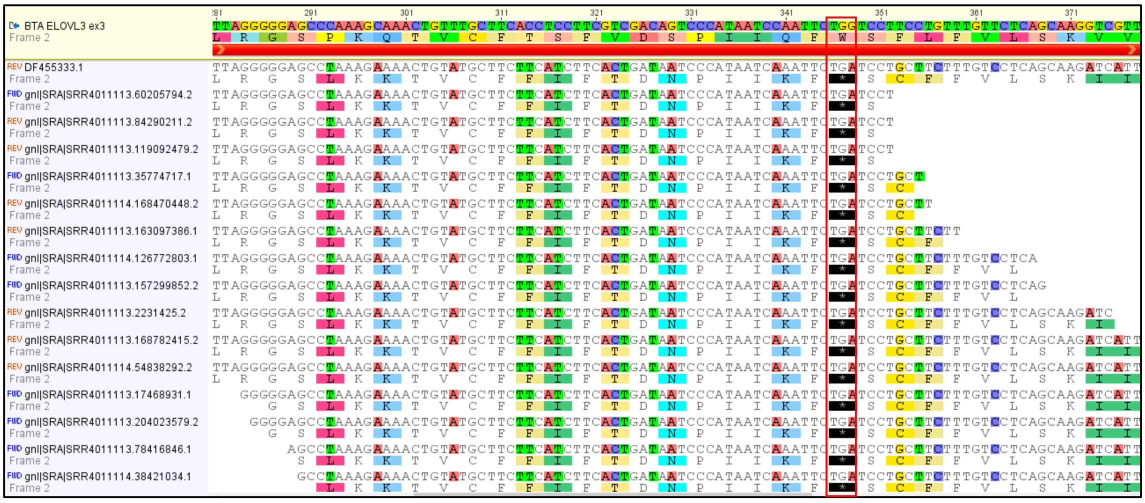

Eschrichtius robustus
SRA searched
SRR5495100- Purdue University 2017-05-02 **Sample ID:** SAMN06837694 (GFD-02)
SRR5495106- Purdue University 2017-05-02 **Sample ID:** SAMN06837692 (ER-14-168)

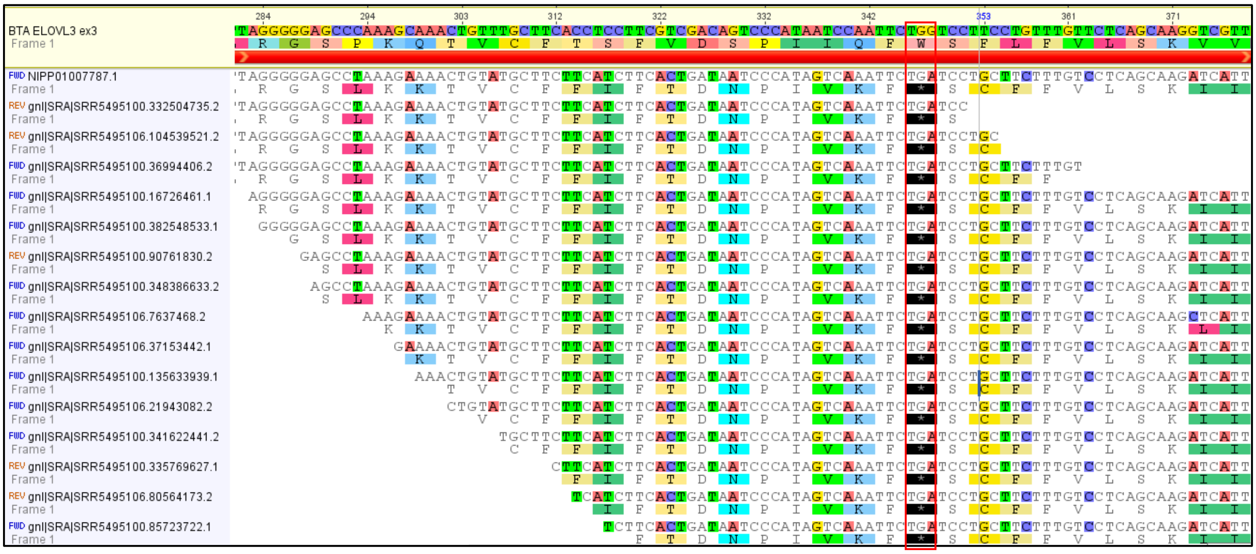

Balaena mysticetus
SRA searched
SRR1685385- University of Liverpool 2015-01-06 **Sample ID:** SAMN03225705 (Bowhead ID325)
SRR1685386- University of Liverpool 2015-01-06 **Sample ID:** SAMN03225705 (Bowhead ID325)

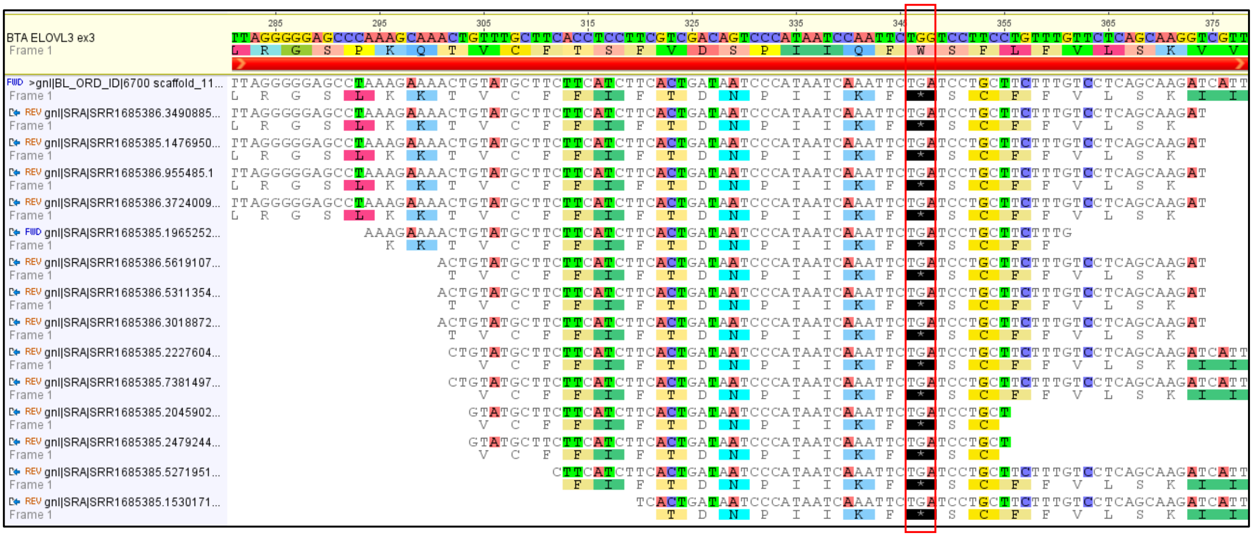

SRA validation of Exon1 splice site mutation of ***FABP9***

Orcinus orca

SRA searched

SRR1164379- University of Durham 2014-02-13 **Sample ID:** SAMN02595096 (AR-Genome)

SRR574977- Baylor College of Medicine 2012-09-20 **Sample ID:** SAMN01180276 (AForca1)

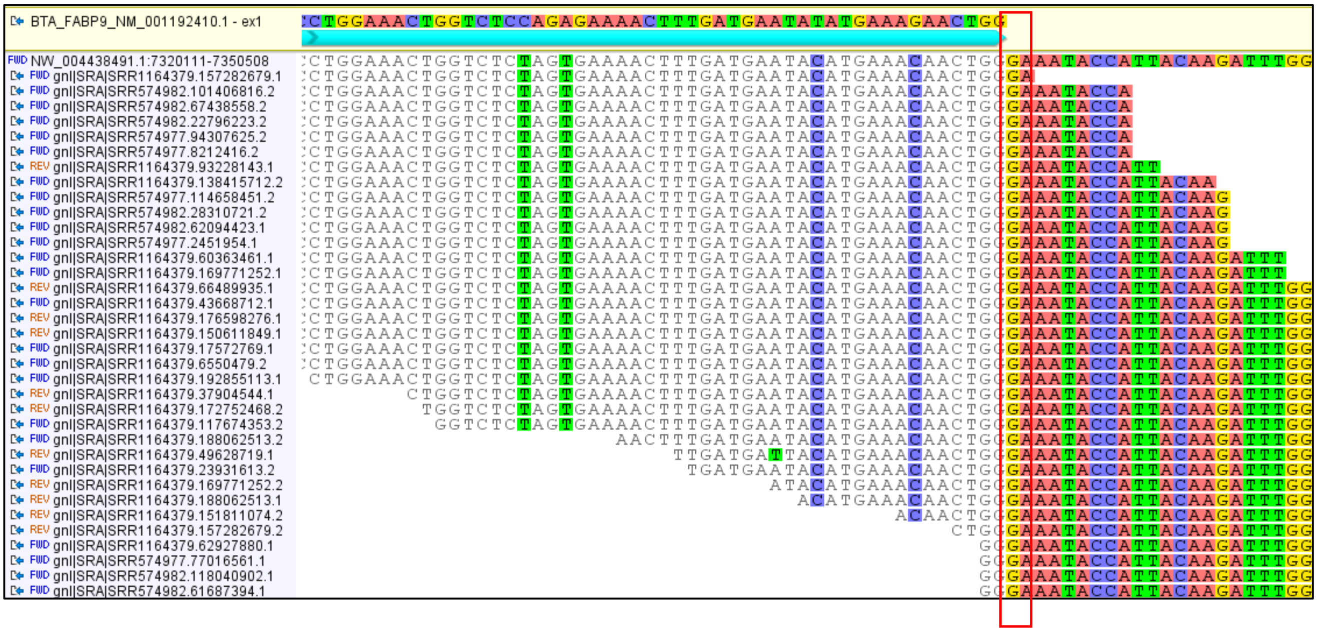

Tursiops truncatus

SRA searched

SRR5125024- National Institute of Standards and Technology 2016-12-27 **Sample ID:** SAMN06114300 (ACU285439A)

SRR606320/21/22/24- Baylor College of Medicine 2015-07-22 **Sample ID:** SAMN00000070

SRR2148845 - Beijing Genome Institute 2016-08-10 **Sample ID**: SAMN03968479

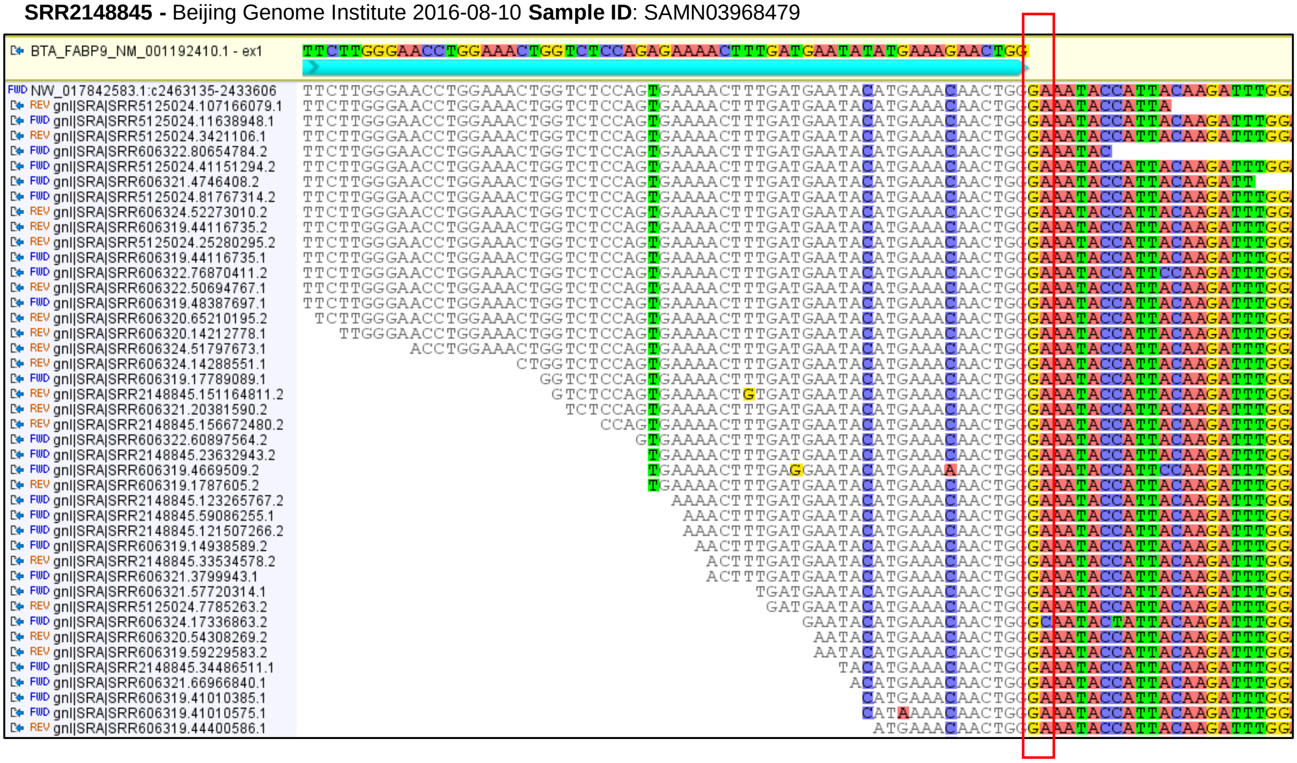

Delphinapterus leucas
SRA searched
SRR5197962- BC Cancer Agency Michael Smith Genome Sciences Centre 2017-06-27 **Sample ID:** SAMN06217832 (Qila21)
SRR5197961- BC Cancer Agency Michael Smith Genome Sciences Centre 2017-06-27 **Sample ID:** SAMN06216270 (Aurora29)

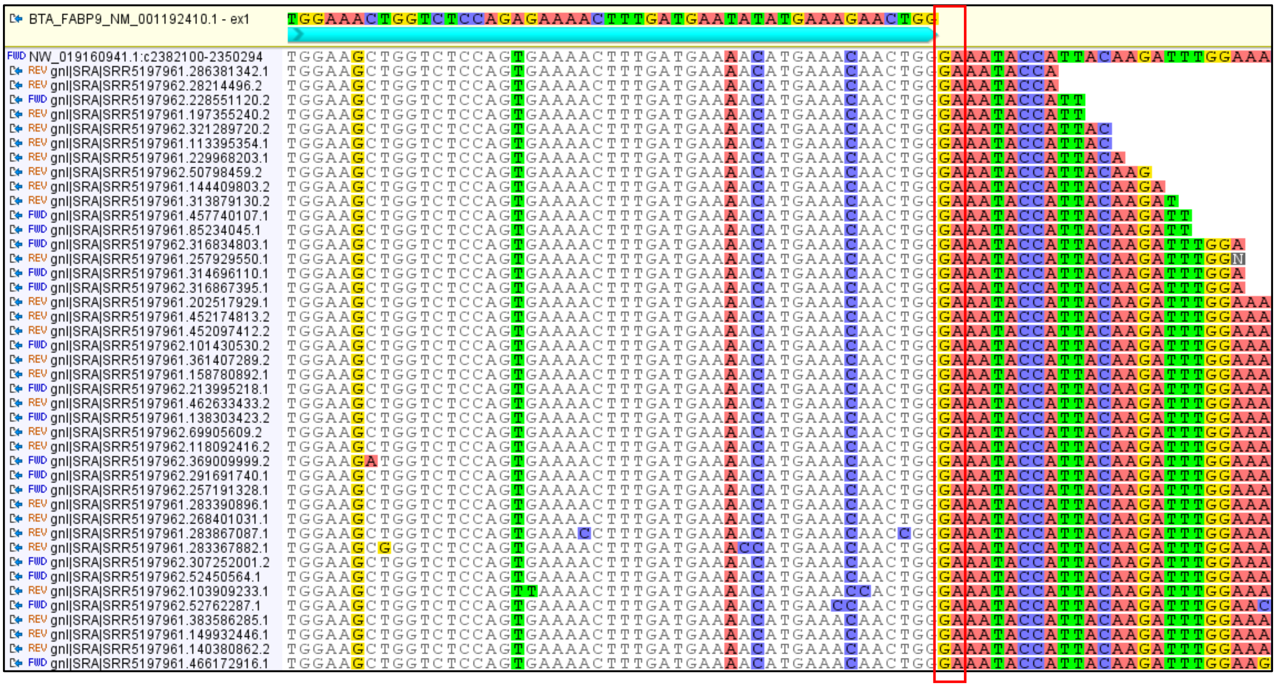

Physeter catodon
SRA searched
SRR5146847- The Genome Center at Washington University School of Medicine in St. Louis 2017-01-05 **Sample ID:** SAMN06187412
SRR5146843- The Genome Center at Washington University School of Medicine in St. Louis 2017-01-05 **Sample ID:** SAMN06187413
SRR5146865- The Genome Center at Washington University School of Medicine in St. Louis 2017-01-05 **Sample ID:** SAMN06187411

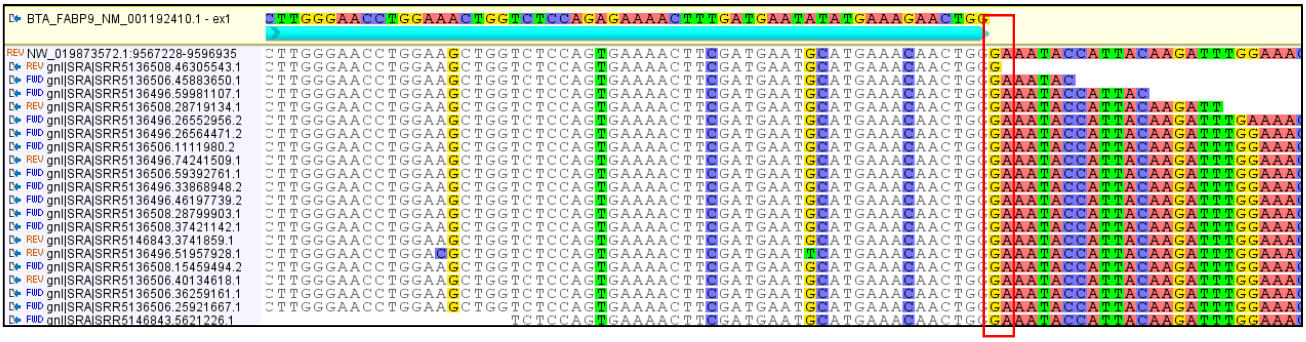

Balaenoptera acutorostrata
SRA searched
SRR924087- Korea Institute of Ocean Science and Technology 2013-10-31 **Sample ID:** SAMN02192644 **(**MinkeWhale-01)
SRR4011112- Institute of Marine Research 2016-08-13 **Sample ID:** SAMN05447714 (AT)

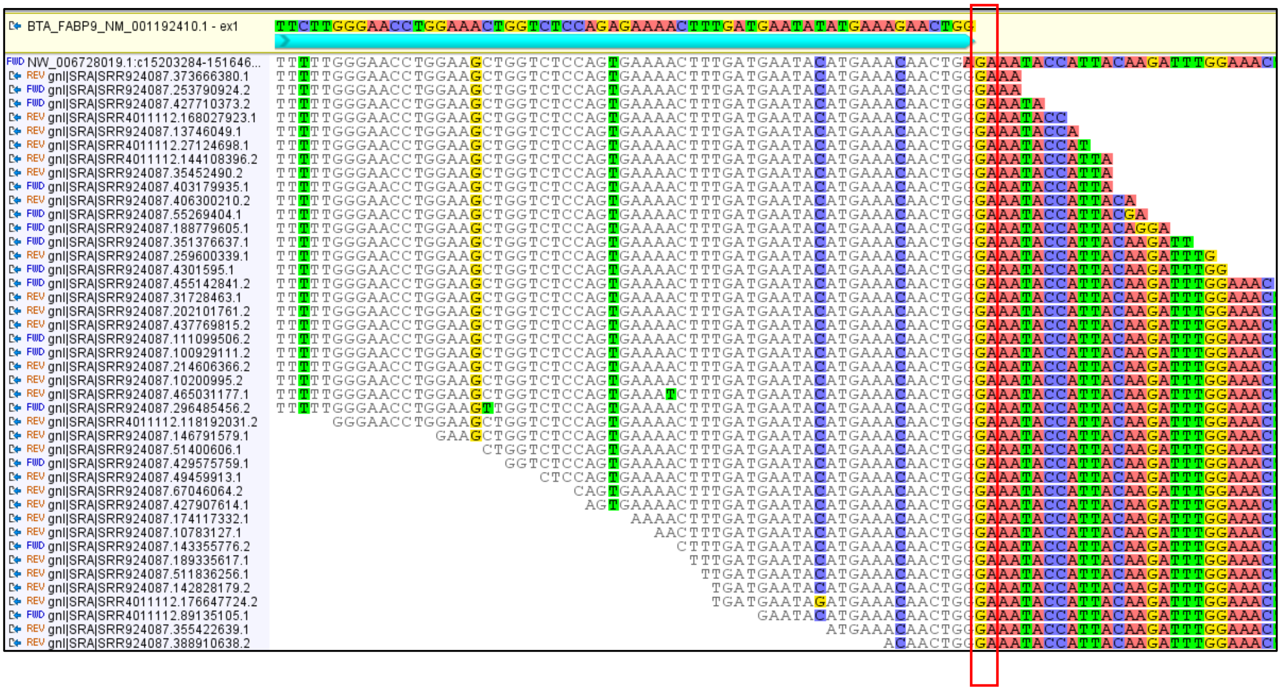

Balaenoptera bonaerensis
SRA searched
SRR4011114- Institute of Marine Research 2016-08-13 **Sample ID:** SAMN05447715
SRR4011113- Institute of Marine Research 2016-08-13 **Sample ID:** SAMN05447715

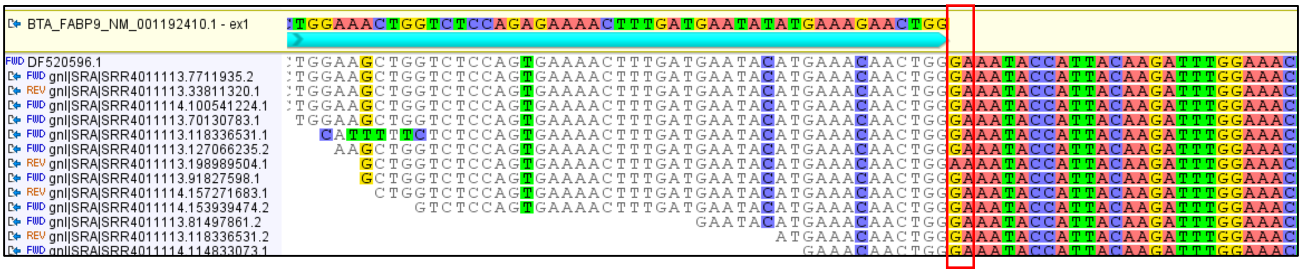

Eschrichtius robustus
SRA searched
SRR5495100- Purdue University 2017-05-02 **Sample ID:** SAMN06837694 (GFD-02)
SRR5495106- Purdue University 2017-05-02 **Sample ID:** SAMN06837692 (ER-14-168)

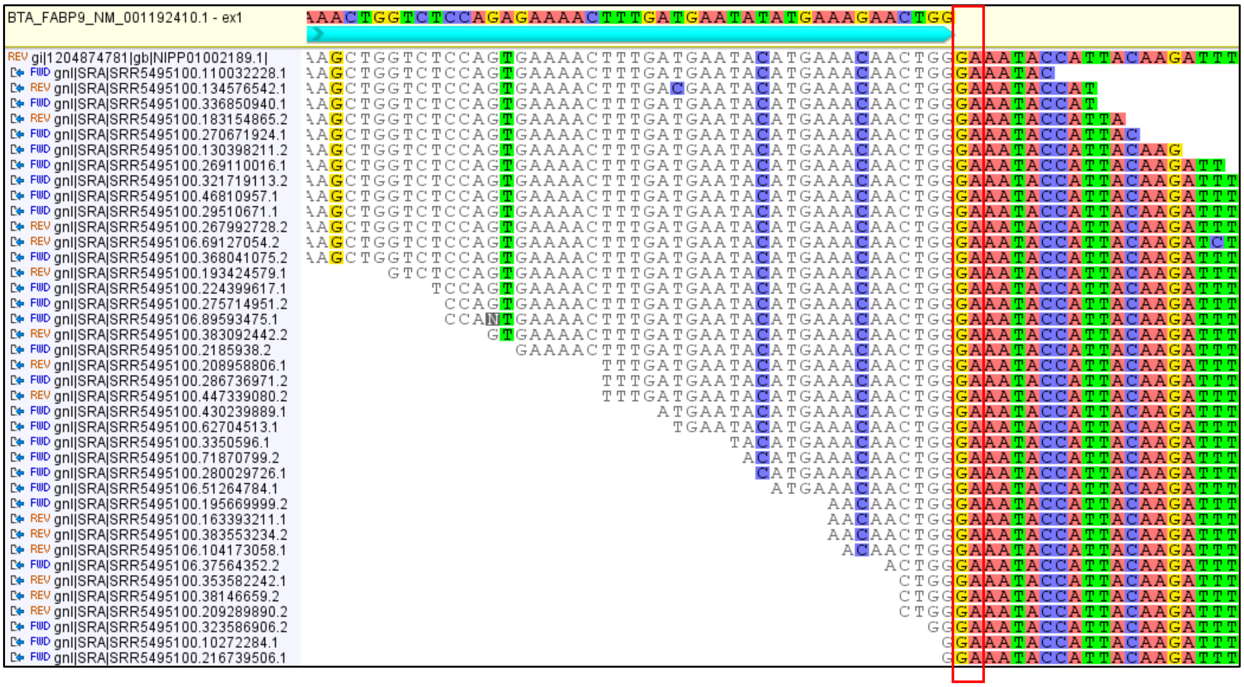

Balaena mysticetus
SRA searched
SRR1685385- University of Liverpool 2015-01-06 **Sample ID:** SAMN03225705 (Bowhead ID325)
SRR1685386- University of Liverpool 2015-01-06 **Sample ID:** SAMN03225705 (Bowhead ID325)

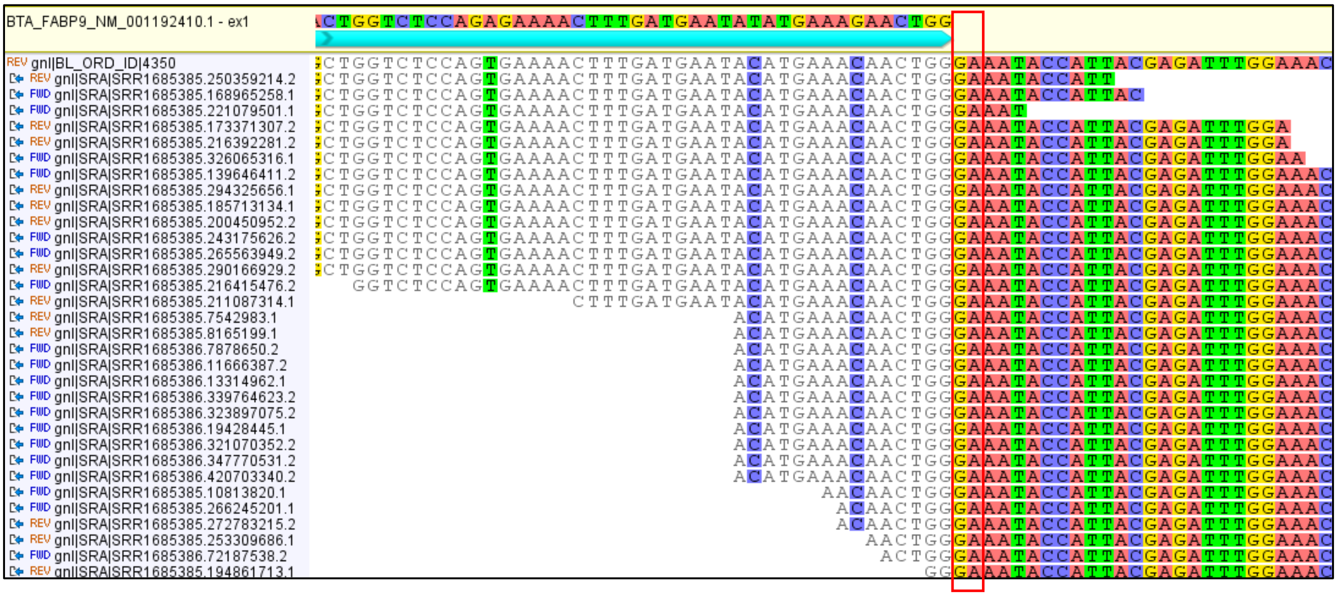

Supplement: msz068_Supplementary_Material [file msz068_supplementary_material.zip › SUPPLEMENTARY_MATERIAL_2.pdf]
