# Supplementary material for: Complete Inactivation of Sebum-Producing Genes Parallels the Loss of Sebaceous Glands in Cetacea
Source: Mol Biol Evol. 2019 Mar 20;36(6):1270–80. doi: 10.1093/molbev/msz068 (PMC6526905; doi:10.1093/molbev/msz068)
Supplement: msz068_Supplementary_Material [file msz068_supplementary_material.zip › SUPPLEMENTARY_MATERIAL_3.pdf]

Supplementary Material 3- SRA validation of mutations in other mammals

Hippopotamus amphibius

***Hippopotamus amphibius* - *MOGAT3* exon 2**

SRA searched

SRR5663647- Senckenberg Gesellschaft fuer Naturforschung 2018-04-05 **Sample ID:** SAMN07206988 (HIP001)

SRR5663648- Senckenberg Gesellschaft fuer Naturforschung 2012-09-20 **Sample ID:** SAMN07206988 (HIP001)

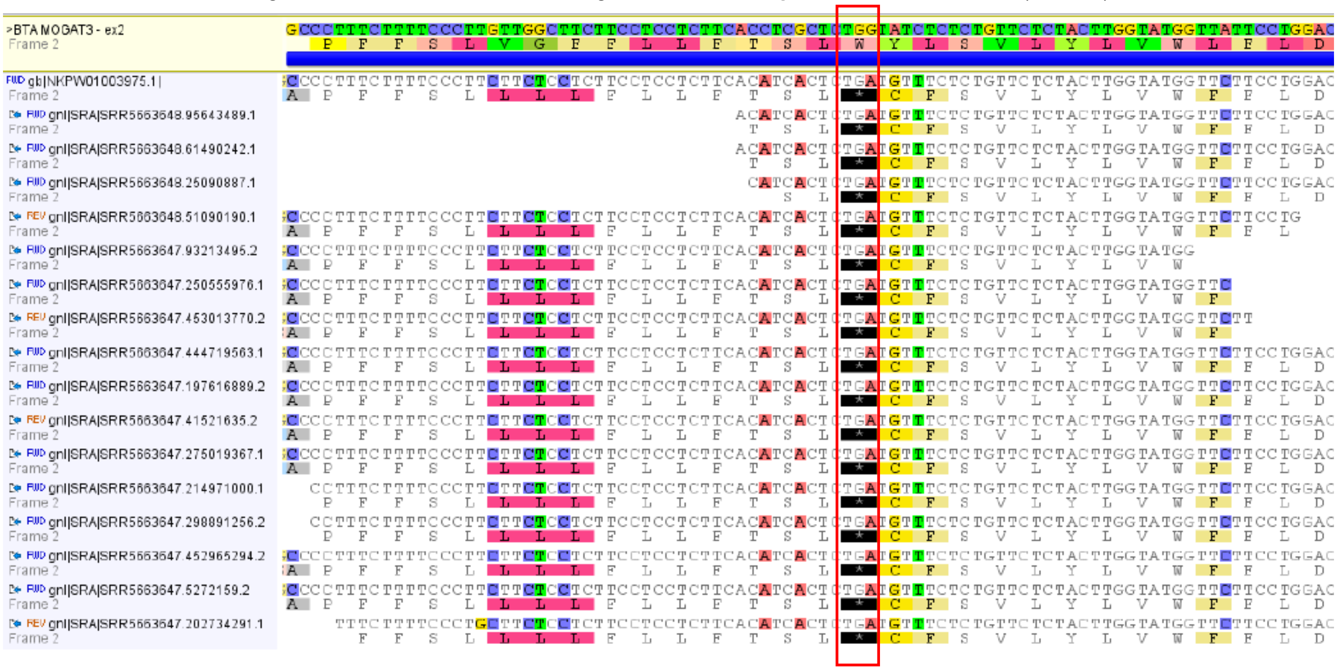

Hippopotamus amphibius* - *MOGAT3 vs MOGAT3-like

SRA searched

SRR5663647- Senckenberg Gesellschaft fuer Naturforschung 2018-04-05 **Sample ID:** SAMN07206988 (HIP001)

SRR5663648- Senckenberg Gesellschaft fuer Naturforschung 2012-09-20 **Sample ID:** SAMN07206988 (HIP001)

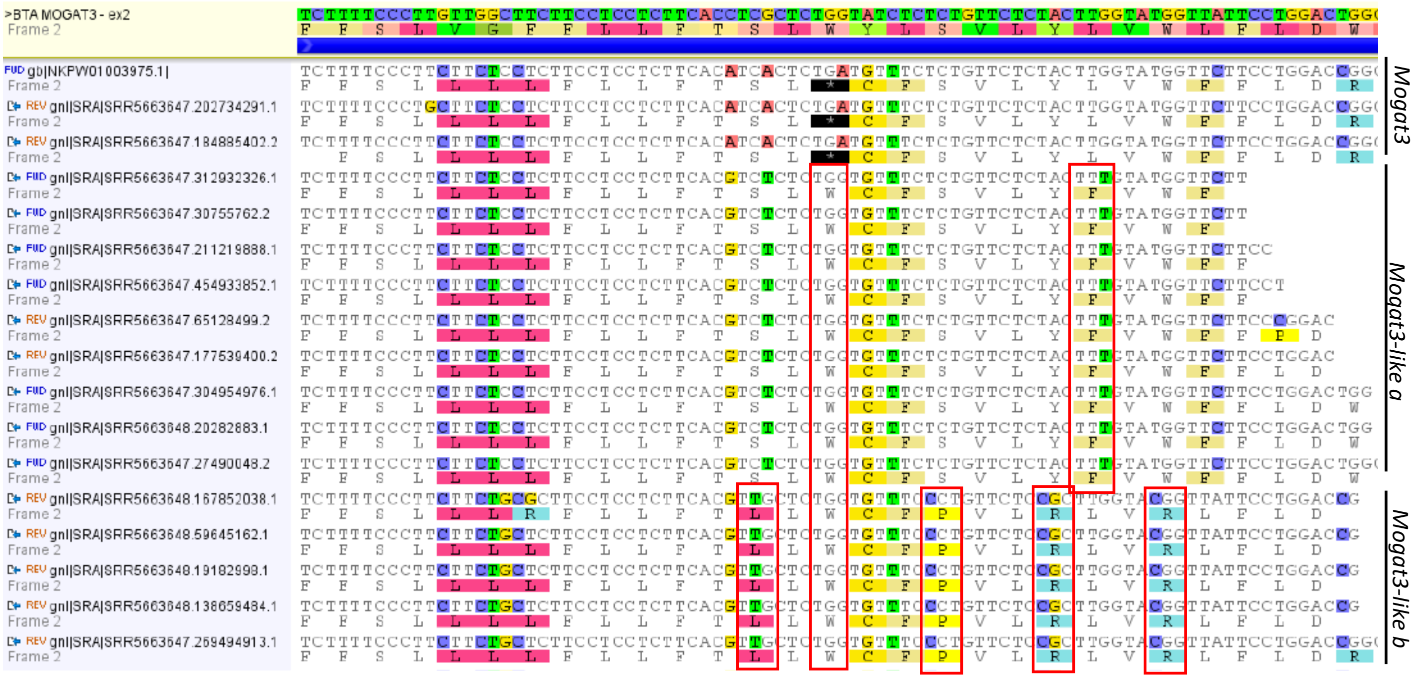

***Hippopotamus amphibius* - *MOGAT3* exon2 Transcriptomic reads**
SRA searched

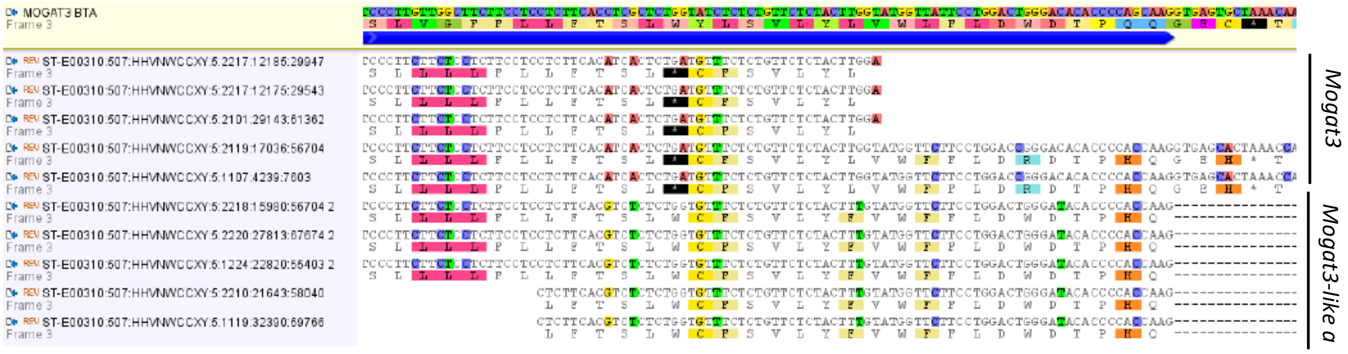

***Hippopotamus amphibius* - *AWAT1* exon 3**
SRA searched
SRR5663647- Senckenberg Gesellschaft fuer Naturforschung 2018-04-05 **Sample ID:** SAMN07206988 (HIP001)

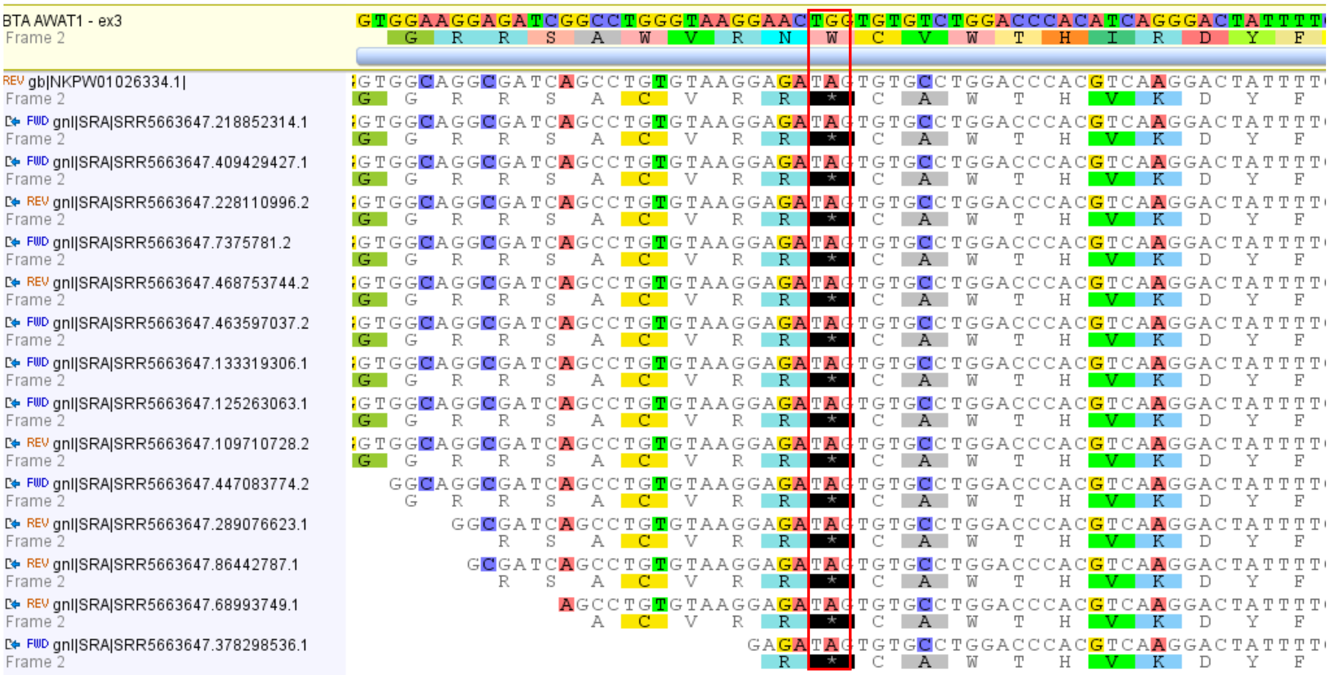

Trichechus manatus latirostris

Trichechus manatus latirostris* – *MOGAT3 exon4

SRA searched

SRR4228537- NCBI GEO 2017-10-11 **Sample ID:** SAMN05761505 (transcriptomic)

SRR331137 /39/ 42 - Broad Institute (BI) 2011-08-17 **Sample ID:** SAMN00632092 (Lorelei) (Genomic)

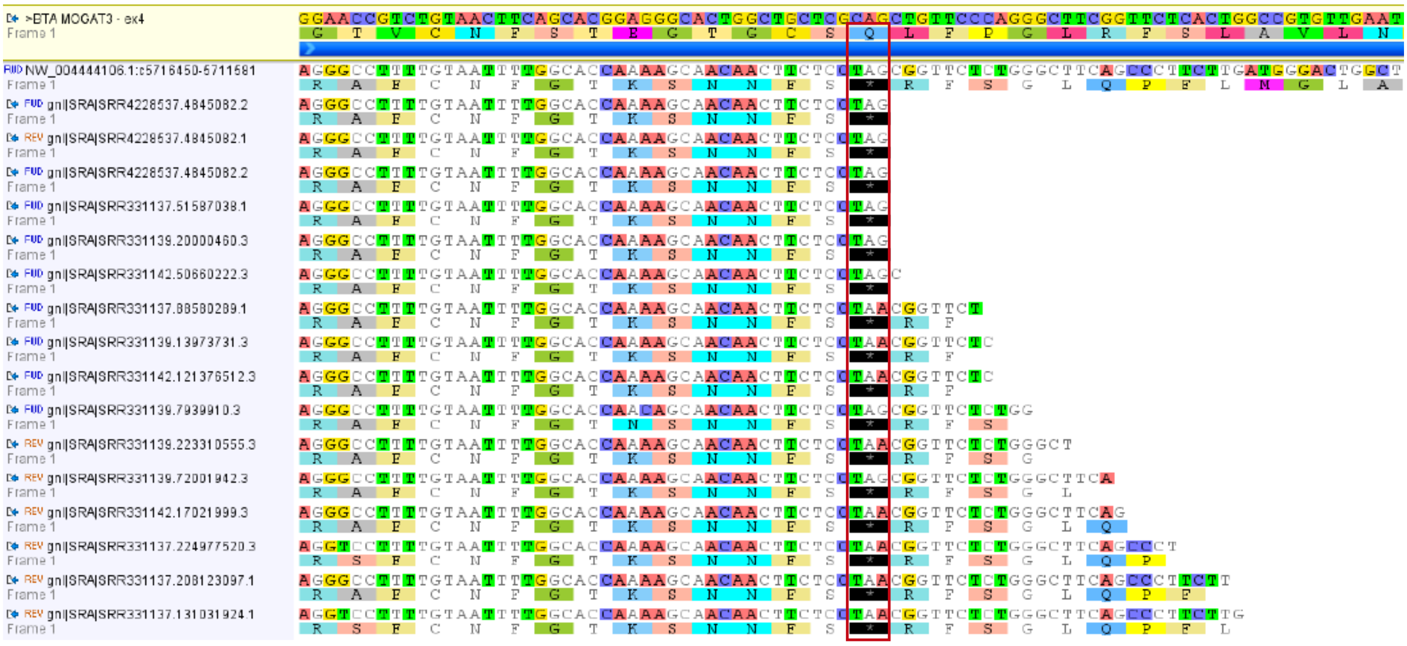

Trichechus manatus latirostris* – *AWAT2 exon 2

SRA searched

SRR4228537- NCBI GEO 2017-10-11 **Sample ID:** SAMN05761505 (transcriptomic)

SRR331137 /39/ 42 - Broad Institute (BI) 2011-08-17 **Sample ID:** SAMN00632092 (Lorelei) (Genomic)

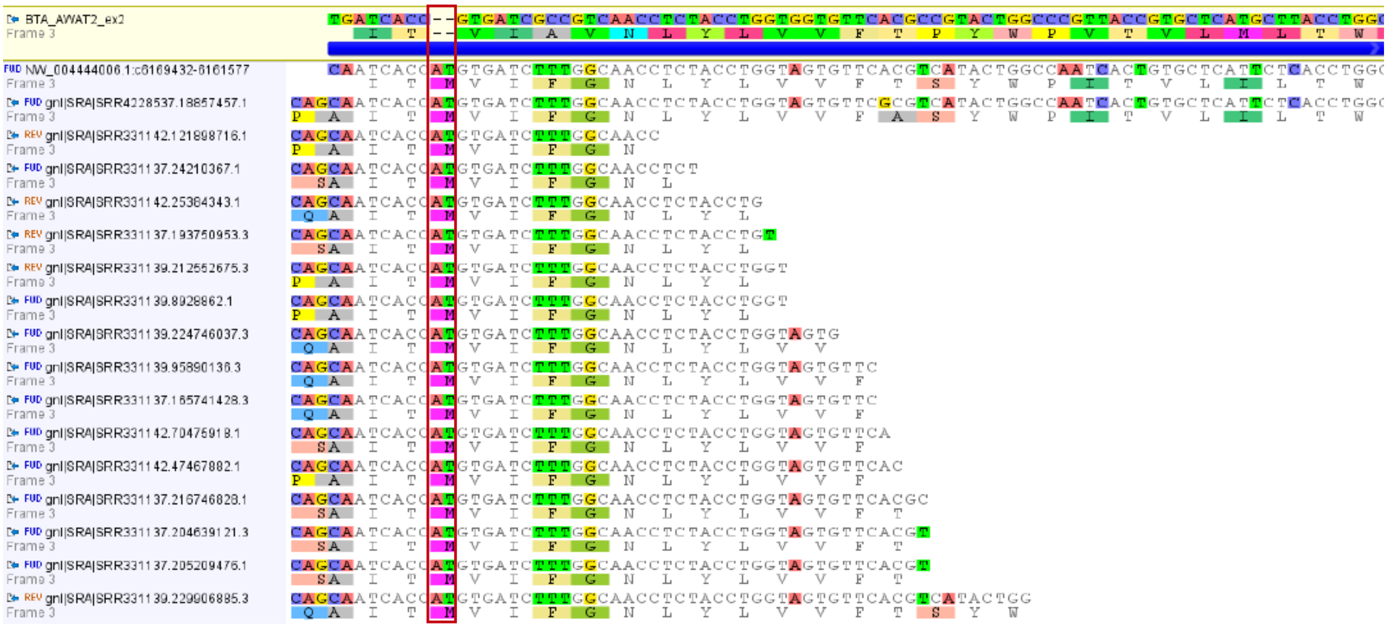

Sus scrofa

***Sus scrofa* MOGAT3 exon 3**

SRA searched

SRR5947501 - Jiangxi Agricultural University 2017-09-12 **Sample ID:** SAMN07503176 **(**Bamei)

SRR5351767- China Agricultural University 2018-04-01 **Sample ID:** SAMN06611050 (Duroc 4)

SRR5947513 - Jiangxi Agricultural University 2017-09-12 **Sample ID:** SAMN07503176 (Baoshan)

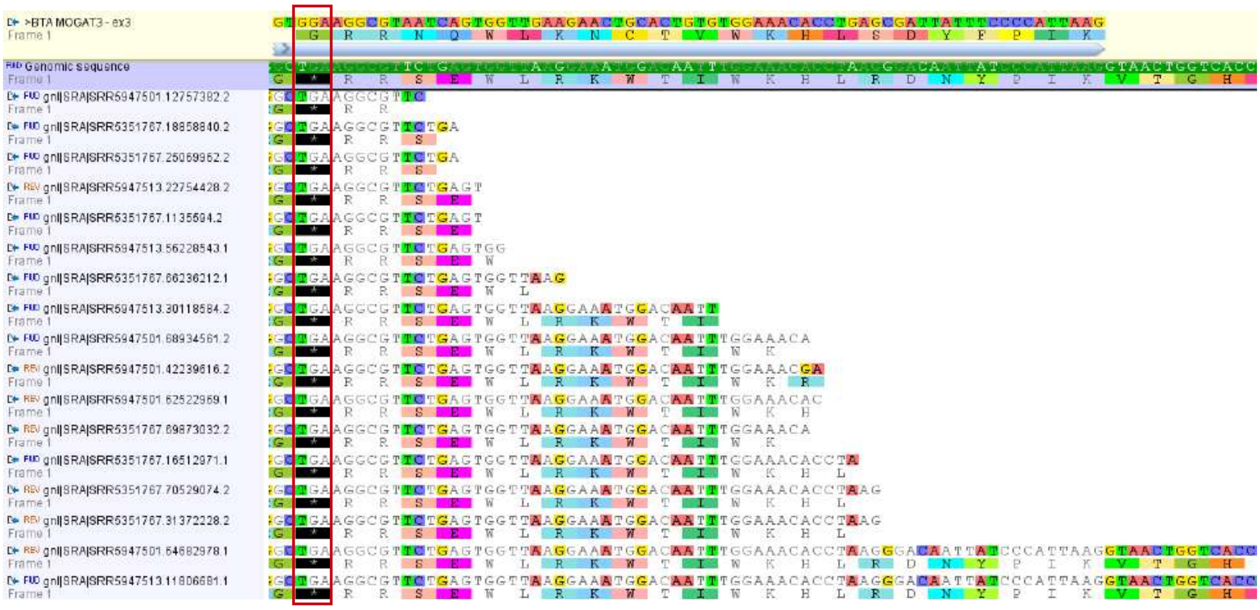

***Sus scrofa* AWAT1 exon2**

SRA searched

SRR5947501 - Jiangxi Agricultural University 2017-09-12 **Sample ID:** SAMN07503176 **(**Bamei)

SRR5351767- China Agricultural University 2018-04-01 **Sample ID:** SAMN06611050 (Duroc 4)

SRR5947513 - Jiangxi Agricultural University 2017-09-12 **Sample ID:** SAMN07503176 (Baoshan)

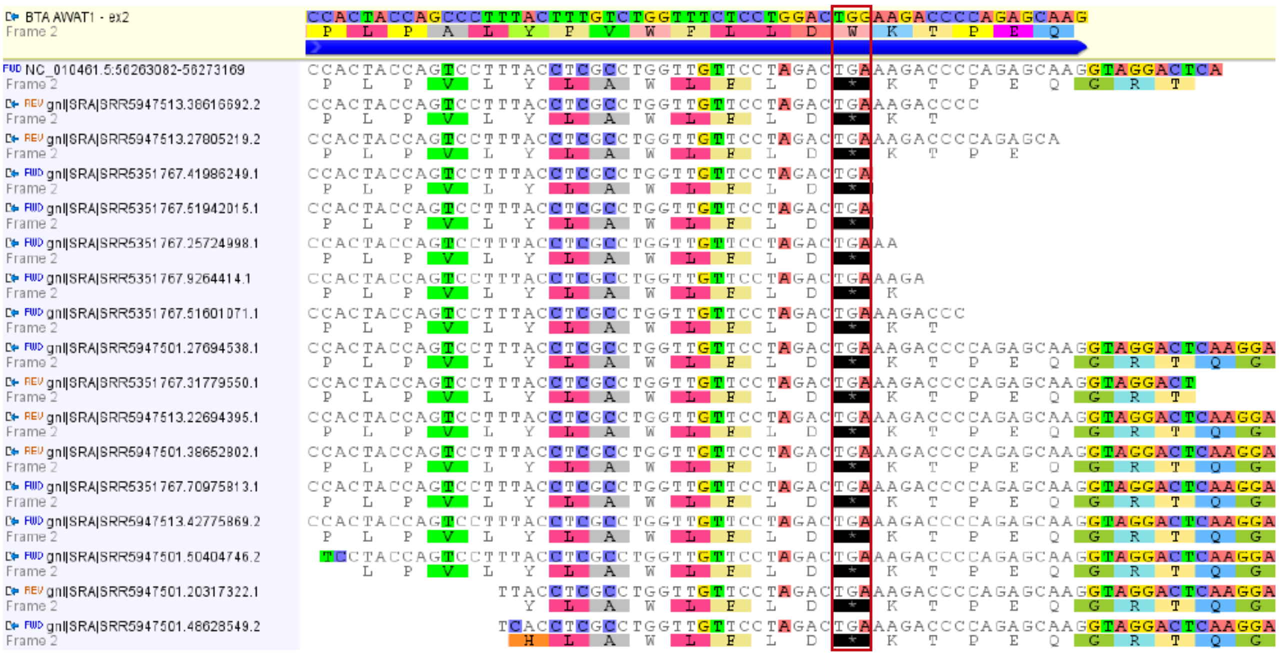

Sus scrofa AWAT 2 exon3

SRA searched

SRR5947501 - Jiangxi Agricultural University 2017-09-12 **Sample ID:** SAMN07503176 **(**Bamei)

SRR5351767- China Agricultural University 2018-04-01 **Sample ID:** SAMN06611050 (Duroc 4)

SRR5947513 - Jiangxi Agricultural University 2017-09-12 **Sample ID:** SAMN07503176 (Baoshan

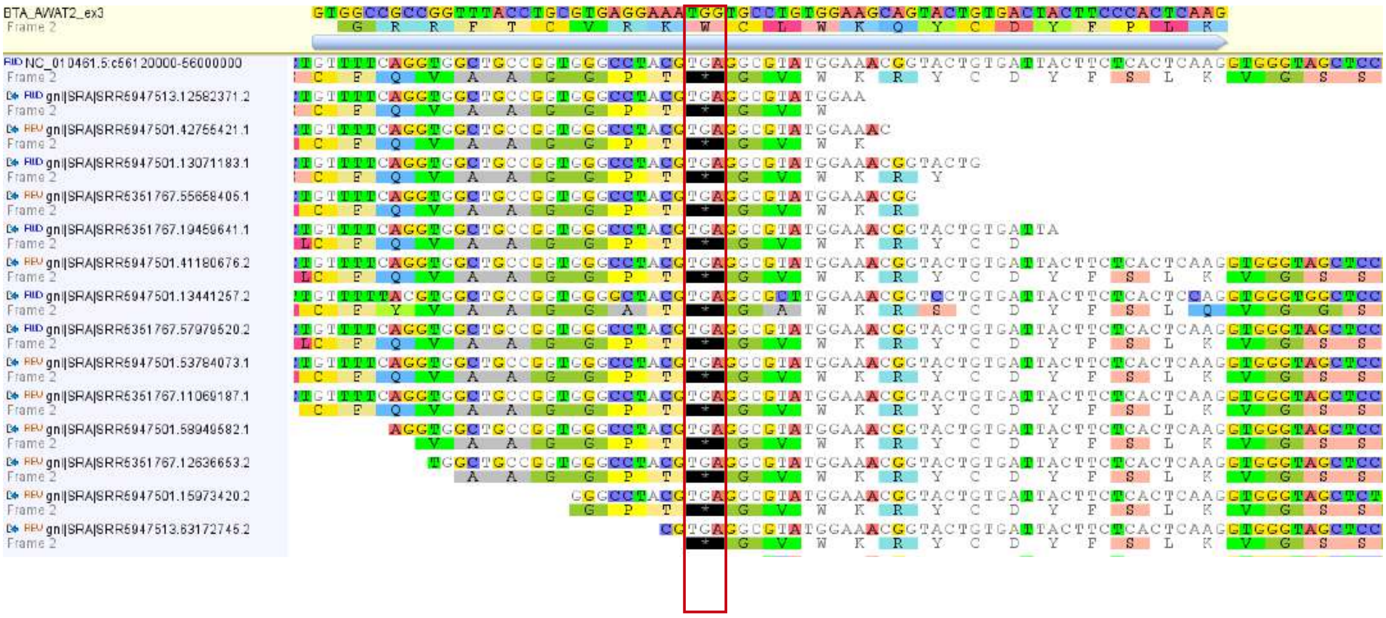

Loxodonta africana

***Loxodonta africana* – MOGAT3**
SRA searched
SRR958468- Lausanne University, Cig, Genopode 2014-04-23 **Sample ID:** SAMN02333826 (female)
SRR958467- Lausanne University, Cig, Genopode 2014-04-23 **Sample ID:** SAMN01180276 (male)

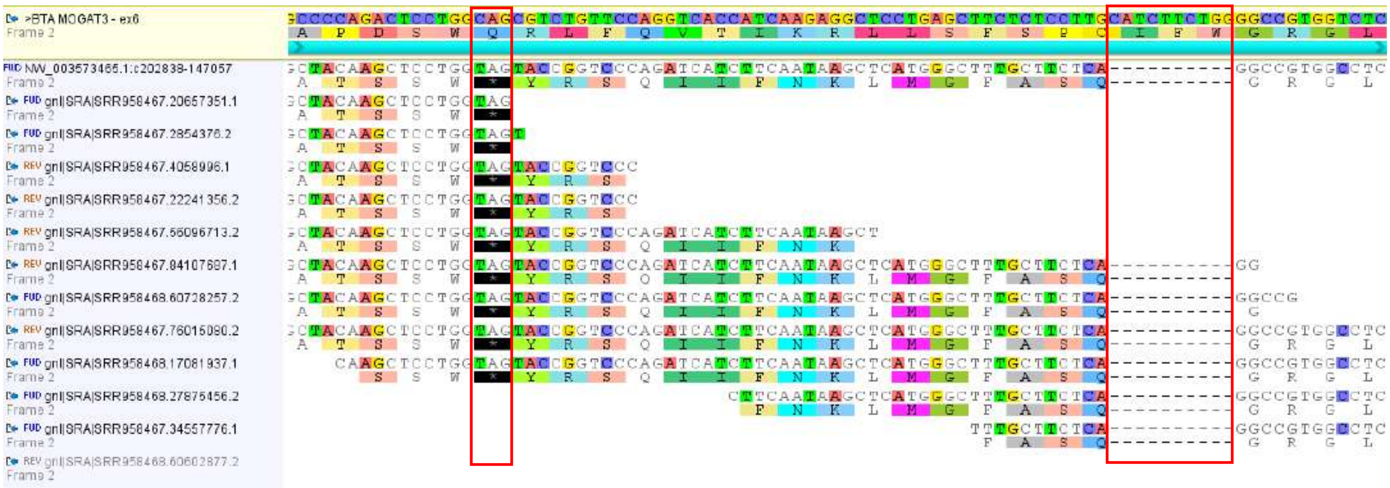

***Loxodonta africana* – AWAT2**
SRA searched
SRR958468- Lausanne University, Cig, Genopode 2014-04-23 **Sample ID:** SAMN02333826 (female)
SRR958467- Lausanne University, Cig, Genopode 2014-04-23 **Sample ID:** SAMN01180276 (male)

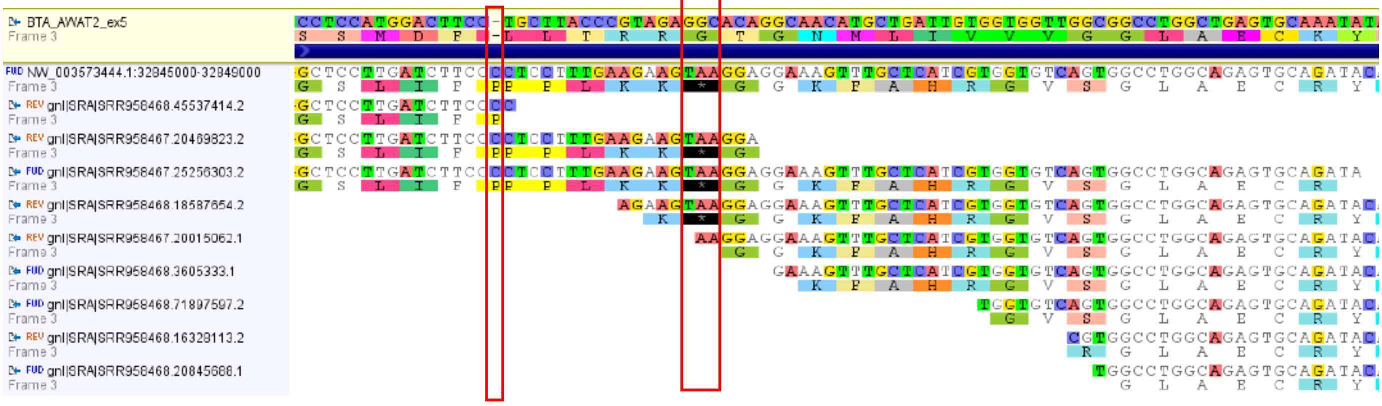

Ceratotherium simum simum

***Ceratotherium simum simum* MOGAT3 exon2**
SRA searched
SRR403463/64/66- Broad Institute (BI) 2012-01-31 **Sample ID:** SAMN00778988

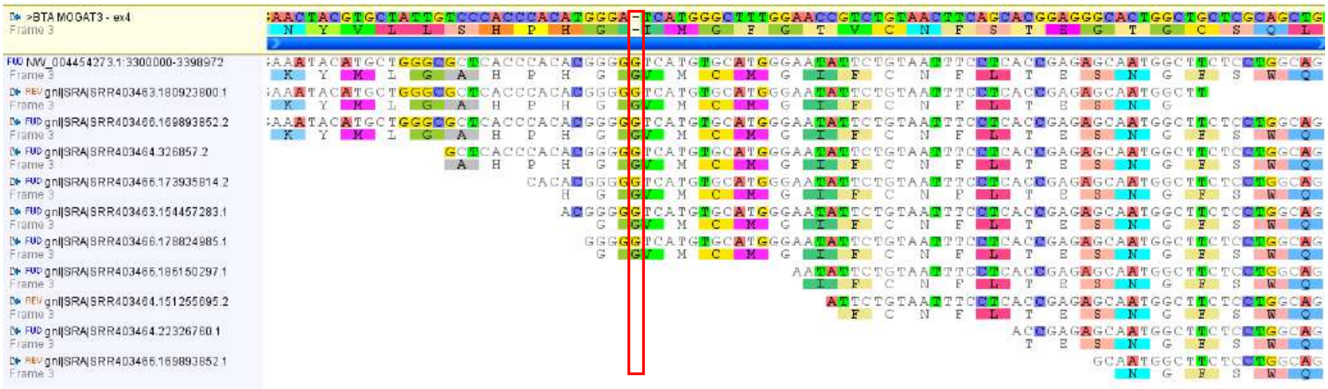

***Ceratotherium simum simum* DgGAT2L6 exon2**
SRA searched
SRR403463/64/65/66- Broad Institute (BI) 2012-01-31 **Sample ID:** SAMN00778988

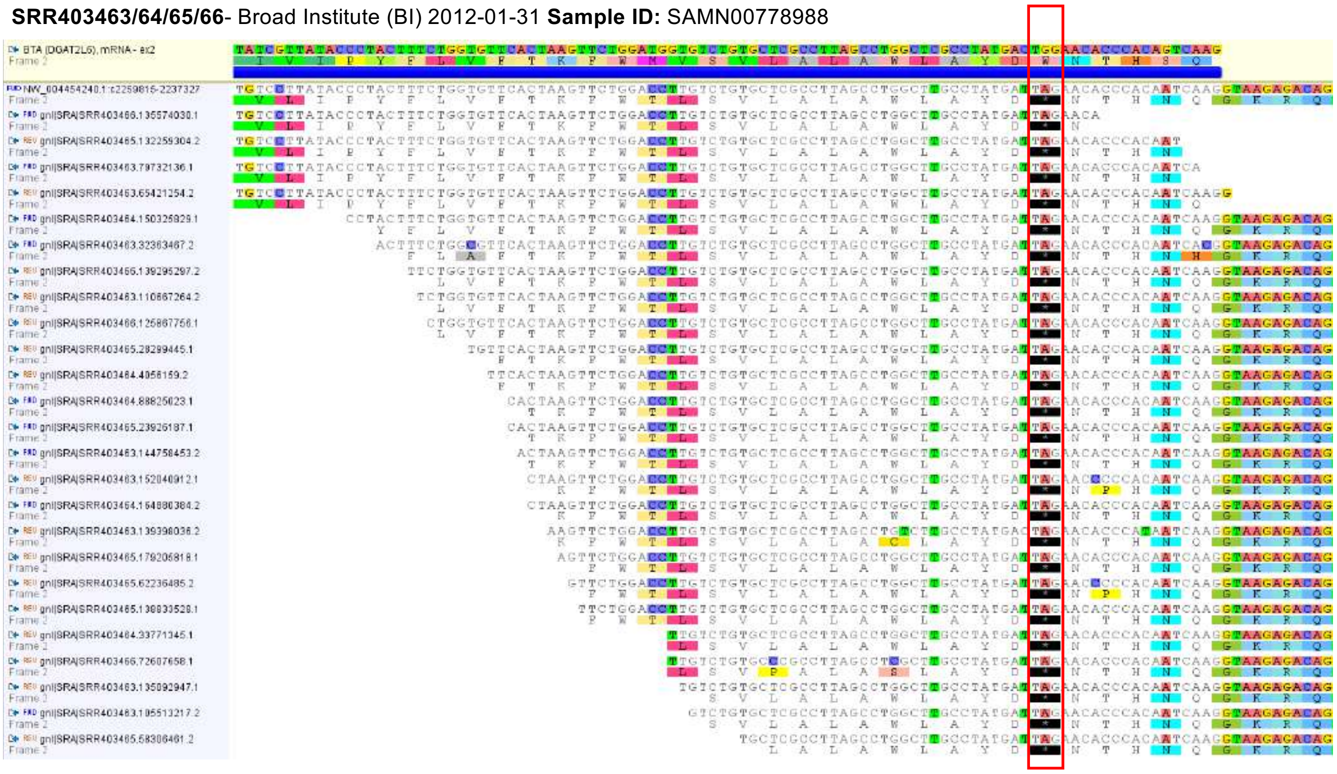

Ceratotherium simum simum AWAT1 exon2
SRA searched
SRR403463/64/65/66- Broad Institute (BI) 2012-01-31 **Sample ID:** SAMN00778988

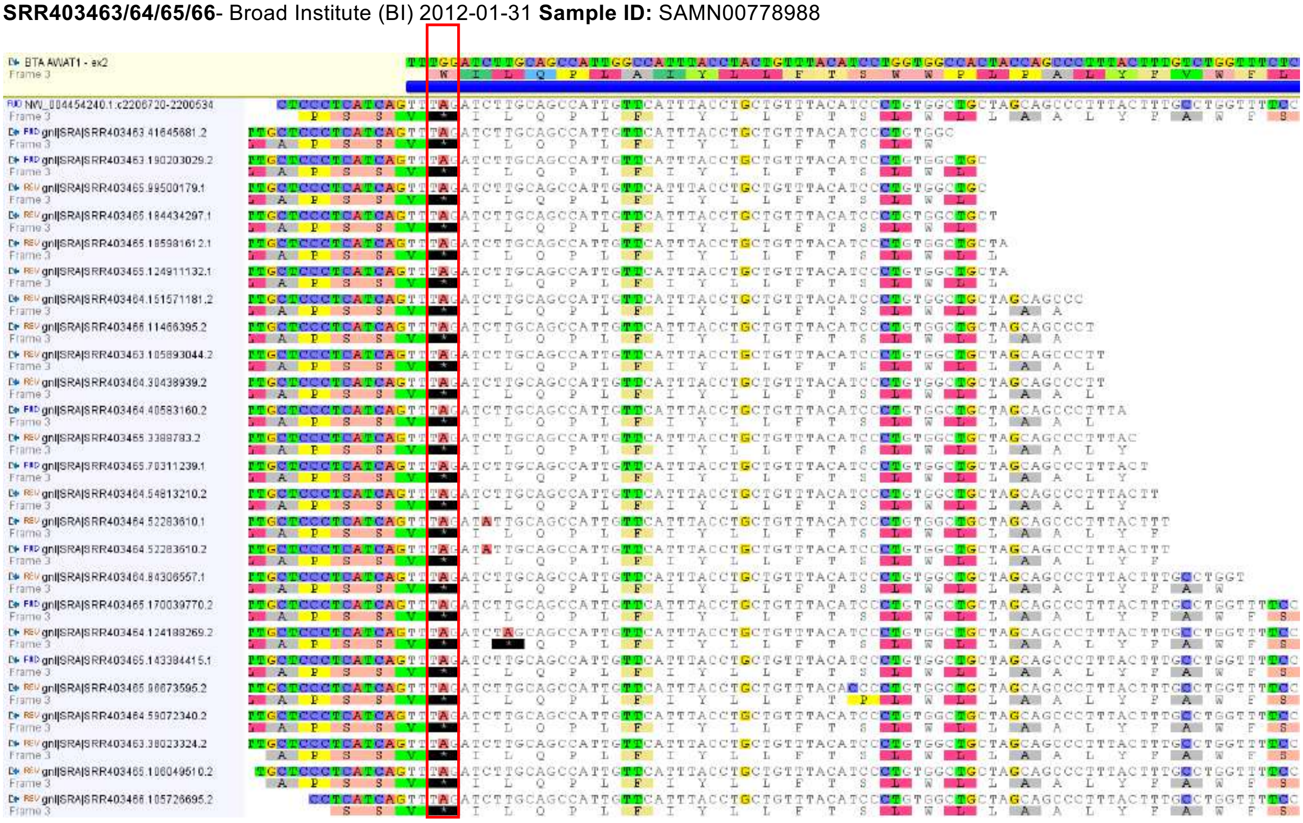

Ceratotherium simum simum FABP9
SRA searched
SRR403463/64/65/66- Broad Institute (BI) 2012-01-31 **Sample ID:** SAMN00778988

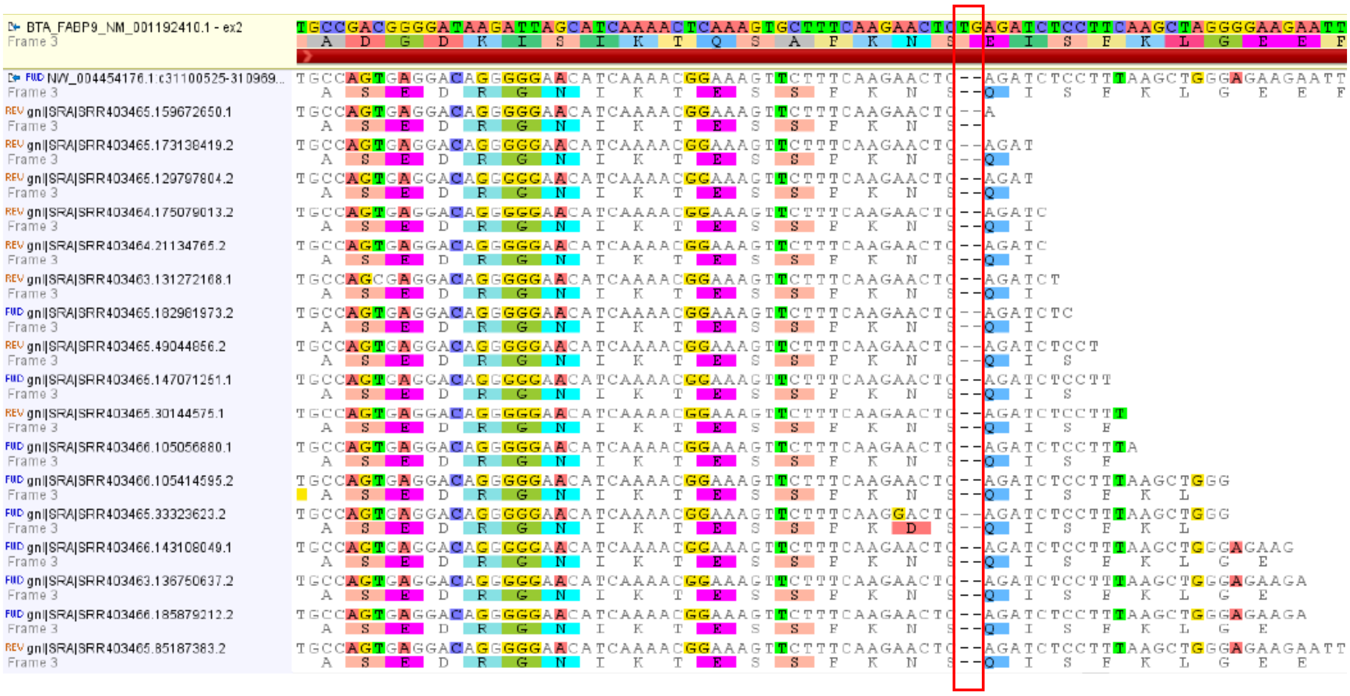

Multiple sequence alignment of studied genes in selected mammal species

Identified mutations are boxed: orange – start codon missing; red – stop codon; blue – deletion, and green – insertion. Alternating shade indicates exons.

Sequence alignment of predicted *Mogat3*.

```
                            10        20        30        40        50        60        70        80
                    ....|....|....|....|....|....|....|....|....|....|....|....|....|....|....|....|
B. taurus           ATGAAAACCTTAAAGAAACAGTGGTTAGAAGTACTGAGCACCTACCAATATGTGCTCTGTTTCCTCTTCCTGGGCCCTTT
S. scrofa           C........C.GT......-.CT.C.G......T.....GT.....T.ACG......AC................A....
H. amphibius        ...G.....C.GC.....G.....C....................T.C..C......AC....G.....A...C......
C. s. simum         -------------------------------------------------------------------------.......
T. m. latirostris   .G.......C.G.....G...C.T......AC.G..GC.G.........T..G.T..A...........T.....GT...
L. africana         -------------------------------------------------------------------------C..AGG.

                            90       100       110       120       130       140       150       160
                    ....|....|....|....|....|....|....|....|....|....|....|....|....|....|....|....|
B. taurus           CTTTTCCCTTGTTGGCTTCTTCCTCCTCTTCACCTCGCTCTGGTATCTCTCTGTTCTCTACTTGGTATGGTTATTCCTGG
S. scrofa           .C.G..TG..CC.TT.CC.C..-....T.....AGA.T.T....T.T.........C...T....C.C..C.C.A.....
H. amphibius        ..........C..CT.C................A..A.....A.G.T.........................C.......
C. s. simum         ..........C.---.C.A............C.G..........CG..............T.CA..G...C.C.......
T. m. latirostris   .C.C......C.---.C..C................AT......CAT..................CC...C.C.A.....
L. africana         G.GCATGGC...GCT.C...CT..G..GG.AGG.CG.GC....ATC..AG.G..C......C....C...C.C.ATAGA.

                           170       180       190       200       210       220       230       240
                    ....|....|....|....|....|....|....|....|....|....|....|....|....|....|....|....|
B. taurus           ACTGGGACACACCCCAGCAAGGTGGAAGGCGTAATCAGTGGTTGAAGAACTGCACTGTGTGGAAACACCTGAGCGATTAT
S. scrofa           ..........G...TTC.....CT........TC.G.......A.G...A..G..AA.T...........A..G..CA..
H. amphibius        ..C.............C...........A...TC.G.A...A...G......G...C.............A..T......
C. s. simum         .....C........A.C..............CTC.G.....A.A.GA.G...G.....T..............G......
T. m. latirostris   ..........G...AGC....A.......GA.TC.G.....A.A.GA.....G..AA.T...........A..G......
L. africana         .TA.A...T....T.G.ACG..A..CC.C..CTC.GCC...G.CCGC.....GG.G..C....G....T.CT.T..C...

                           250       260       270       280       290       300       310       320
                    ....|....|....|....|....|....|....|....|....|....|....|....|....|....|....|....|
B. taurus           TTCCCCATTAAGCTGGTGAAAACAGTAGAGCTGCCCCCAGACAGGAACTACGTGCTATTGTCCCACCCACATGGGA-TCA
S. scrofa           .AT....................T...........T..T...CA.....GTA..G..G.----.....C.......-...
H. amphibius        ..T.......................C...........CA..C.............GG.C..T...........A.-...
C. s. simum         .AT..T..CGG...............G..........TCA..C....A...A....GGGCG.T........C...GG...
T. m. latirostris   ..T..T..C................C.........T..CA..CA.......C..A.GGGCG.T....TG..CA...-...
L. africana         .....A..CTC..............C.........T..CA..CA..........A.GGGCGTT.............-...

                           330       340       350       360       370       380       390       400
                    ....|....|....|....|....|....|....|....|....|....|....|....|....|....|....|....|
B. taurus           TGGGCTTTGGAACCGTCTGTAACTTCAGCACGGAGGGCACTGGCTGCTCGCAGCTGTTCCCAGGGCTTCGGTTCTCACTG
S. scrofa           ..A..ACCAC.G..C.......T...TCT..CA..A....CA...T...C.....C.....TT.AG.GA.TC.TGAG...
H. amphibius        .AAA...G..G.G.AA.ATG......GC...T....A......A.T...C...AA....T.T...A..T..CC..TT.CA
C. s. simum         ..T..A.G....TAT.......T...CT...C...A...A.....T...CTG..A...T..T......T..CC.CGGACA
T. m. latirostris   C.T..AGCA.GG..T.T.....T..TG....CA.AA...ACAA..T...CT...G....T.T.......A.CC..TCT..
L. africana         ..T..GC...GG..T.......T........C..A....AG.A..T...C.T..A......C......G..CC...C.C.

                           410       420       430       440       450       460       470       480
                    ....|....|....|....|....|....|....|....|....|....|....|....|....|....|....|....|
B. taurus           GCCGTGTTGAATTGTCTCTTGTACGTGCCAGGCTGTCGAGAGTACATTATGTCCTGTGGAACATGTTCTGTGAACCGTCA
S. scrofa           AAT.GTGG..GGA.G.C.CCTAC.T....TCC.CCCAC.CCCCC.GCCCCCA.AGAGT----------------------
H. amphibius        A.A.G.C.....G.CA....C...C.C.......A.......C.AG..C.A...CT....CTG.....C....G...C.G
C. s. simum         ..T.CCC..GCCG.CG....CC..C.C....T..T...CA.C.....C.......AC...CTG.....C........CTG
T. m. latirostris   ATG.GAC..GC.-.C.....CA.TC.T...AT..T...CA.C...G.C...AG.AC....GTG...C...C..G.T.C..
L. africana         ATG.CAC..GCCG.C.....CAG.C.C....T..A..AT..CC..C.C...AG.AC.....T......C....G..AC..

                           490       500       510       520       530       540       550       560
                    ....|....|....|....|....|....|....|....|....|....|....|....|....|....|....|....|
B. taurus           GAGCCTGGATTATGTTCTATCTCAACCACAGCTCGG--CAGGGCTGTGGTCATCATGGTCGGAGGGGCCAATGAGGCCCT
S. scrofa           --------------------------------------------------------------------------------
H. amphibius        ...........T.A.......G.GG..C........--GCA....A.........CA.CT..G......C.CA..T....
C. s. simum         .........C.T......G.TG..G..C....GG..GG....-.C.........C.......G..T...C.C...T....
T. m. latirostris   C.....A..C.T.A.C..G..CTGG...GGC.AT.A--TCATCA.CATCGAGGGT..CC.A.GAATC..TG.AC.CTGGA
L. africana         ..C...AC.C.T.A.CTC...C..G..C....GT..--.CA...CT..A.......CA.T..G..T.G.C.G...TT...

                           570       580       590       600       610       620       630       640
                    ....|....|....|....|....|....|....|....|....|....|....|....|....|....|....|....|
B. taurus           GCATACCGTCCCAGGGGAGCACTGCCTCACTCTCCGGAATCGTAAAGGCTTCGTCCGCCTGGCACTGAGGCAC-GGTGCC
S. scrofa           ----------------------------------------------------------------------------G...
H. amphibius        .G.CG..A.....................T....T....CT.G.....T..T....A.T..........AG.T-..C...
```

```
C. s. simum         .T.CG.A...........A............T.T.A...G..G....A...T..GA-................-..G...
T. m. latirostris   C..G..T.G.A.C.----------..........T....G.AC....C...T..G.......TG...G.....A....AG
L. africana         .T.CT.GAG......CC........TGTTT....T..G.G..A........T..G.......TG...T....T-..G...

                           650       660       670       680       690       700       710       720
                    ....|....|....|....|....|....|....|....|....|....|....|....|....|....|....|....|
B. taurus           TCCCTGGTGCCCG----------TGTACTCCTTTGGGGAGAATGACGTCTTCAGAGTTAAGGCTTTTGCCCCAGACTCCT
S. scrofa           ............A----------CC....................T.......A.A.G................G.....
H. amphibius        ..T..........----------..........C...........T......................G.........G.
C. s. simum         ..T........T.----------.A....T...............T.........C.....A........A...G.....
T. m. latirostris   .GT.A..GC.T..CTGGTACCTG.A....................TA......A......A.........A......T..
L. africana         ........A..T.----------......................TA......A........T..C...TA..AG.....

                           730       740       750       760       770       780       790       800
                    ....|....|....|....|....|....|....|....|....|....|....|....|....|....|....|....|
B. taurus           GGCAGCGTCTGTTCCAGGTCACCATCAA-GAGGCTCCTGAGCTTCTCTCCTTGCATCTTCTGGGGCCGTGGTCTCTTCTC
S. scrofa           .....TA..A.C...........T.T..A..A.T..ACAGC.C.T......................ACA..........
H. amphibius        ......A.....GT..A......T....-..A.......G....TG............C............G........
C. s. simum         ......A.....G....AC....T....-..A....A..G....TG....C.................CA..........
T. m. latirostris   .....TACTGA.G......GT..T....-..A....A.AG....TG..............GCA.....CA.........T
L. africana         ..T..TAC.G..C....A...T.T....-T.A....A..G....TG..T..CA----------........C........

                           810       820       830       840       850       860       870       880
                    ....|....|....|....|....|....|....|....|....|....|....|....|....|....|....|....|
B. taurus           AGCCAAGTCCTGGGGCCTGATGCCCCTCGCCAGACCCATCACCACTGTGGTGGGCCGCCCCATCCCGGTGCCCCAGTGTC
S. scrofa           .......C...C.......C.....T.T....A............A.C........A......G.T...A....G...C.
H. amphibius        ......C....A.......G.....T.T.....G...................................C........C.
C. s. simum         G..TG.A............C.....T....TGTG..TG.G...................T...G.T..........C.C.
T. m. latirostris   G..TG.C............C.....T.T.T..TG..A.............---..T.........A.......-..C.C.
L. africana         T..TG.C..T.........C.....T.....GTG.....T...T..T..................A...A.T......C.

                           890       900       910       920       930       940       950       960
                    ....|....|....|....|....|....|....|....|....|....|....|....|....|....|....|....|
B. taurus           CACAGCCCACCGAGGAGCAGGTGGACCACTATCACAGGCTCTACATGAAGGCTCTGGAGCAACTGTTT-GAGGAGCACAA
S. scrofa           .........G.A......................T.T...............................-..A........
H. amphibius        TGTG..................T.............T......................G....C...-...........
C. s. simum         TCA.C....GT......G.A..T..................................T...G.....C-...........
T. m. latirostris   TCA.C.....T.G....G.A.GT.............T.........A...A.......A.........-..........G
L. africana         TCA.C.....T......G.A..T..........................A...A..........T...T...........

                           970       980       990      1000
                    ....|....|....|....|....|....|....|....|....|.
B. taurus           GAAGAGCTGCGGCCTCCCGGCTTCTACTCACCTCACCTTCATCTAG
S. scrofa           .G.A.....T..GG....A.................T.G...TG..
H. amphibius        .G.A.....T..TG....A..........G............G...
C. s. simum         .G.A....CT..GG..G....C......TG..........C.....
T. m. latirostris   .G.A.....T..TG...TT.....................C.....
L. africana         .G.A.....T..TG....T.....................C.....
```

Sequence alignment of predicted *Awat1*.

```
               10        20        30        40        50        60        70        80
                   ....|....|....|....|....|....|....|....|....|....|....|....|....|....|....|....|
B. taurus          ATGCCTTGTTTCAAGCAGCCTAAGCACTTCCAGAGTCTGGTACTTCTGCACTGGCCACTGAGCTACCTTGGCATGTTTTG
S. scrofa          C...........................C...........................C.........A...C...A.....
H. amphibius       ........................................................-.C...........T...A.....
C. s. simum        ......................................CAC.A.......T.....CT............C...A....A
T. m. latirostris  ........C.C............T...............A.G........G.....CT.......T...AC........T
L. africana        ........C.C............TT..............ACG........G.....TT............C...C....T

                            90       100       110       120       130       140       150       160
                   ....|....|....|....|....|....|....|....|....|....|....|....|....|....|....|....|
B. taurus          GATCTTGCAGCCATTGGCCATTTA----CCTACTGTTTACATCCTGGTGGCCACTACCAGCCCTTTACTTTGTCTGGTTT
S. scrofa          ...T......T......T......----...G...........T................T.......C.C.C......G
H. amphibius       .............C..T.......TTTA...G..............C.......C.G.............C.C.......
C. s. simum        ................TT......----...G............CT.....TG...G...............C.......
T. m. latirostris  ................TT......----...G.............T......GT.......G.................G
L. africana        ............G...TT......----...G...........T.TA.....G........AT.........C......G

                           170       180       190       200       210       220       230       240
                   ....|....|....|....|....|....|....|....|....|....|....|....|....|....|....|....|
B. taurus          CTCCTGGACTGGAAGACCCCAGAGCAAGGTGGAAGGAGATCGGCCTGGGTAAGGAACTGGTGTGTCTGGACCCACATCAG
S. scrofa          T....A.....A................A...C...CA..T......................T................
H. amphibius       T...............................C...C....A.....T.......GA.A.....C..........G...A
C. s. simum        TC..............................C...C.T..A.............T....C....T.........G....
T. m. latirostris  T...................T..A.G......C...C.T..A...................C..................
L. africana        T...................T..A........C...C.T..A.................................T..C.

                           250       260       270       280       290       300       310       320
                   ....|....|....|....|....|....|....|....|....|....|....|....|....|....|....|....|
B. taurus          GGACTATTTTCCCATTTCCATCCTGAAGACTAAAGAGCTATCACCAGAGCACAACTATCTCATGGGAGTTCACCCCCACG
S. scrofa          ......C..C........A....A......C.....C..G.....T..ATG......C........CA............
H. amphibius       ..................T........CG.......C..G.....T....................-.G.........T.
C. s. simum        .........C......A.G.................C..G.....TA..........C........G...........T.
T. m. latirostris  .........C......A.G.................C..G.....T....................G..G........T.
L. africana        .........C......A.G.................C..GC....T....................G..G.....T....

                           330       340       350       360       370       380       390       400
                   ....|....|....|....|....|....|....|....|....|....|....|....|....|....|....|....|
B. taurus          GCCTCTTGGCCTTCGGCGCCTTCTGTAACTTCTGCACTGAGGCCACAGGCTTCTTGAAGATCTTTCCAGGCATCACTCCC
S. scrofa          ........A.T..T..T........C.......A......A...........T.C.G.......C.....T..T...T..
H. amphibius       ........A.A..T..T..T.....C....C.......................CC.TC----...........G....T
C. s. simum        .......CA....T...........C............................CA........................
T. m. latirostris  ........A....T..T........C......................AT....C.........C............T.T
L. africana        ........A....T..T........C......................AT....C.........C..............T

                           410       420       430       440       450       460       470       480
                   ....|....|....|....|....|....|....|....|....|....|....|....|....|....|....|....|
B. taurus          CATCTGGCCACGCTGTCCTGGTTCTTCAAGATCCCCTTTATTAGGGACTACCTCATGGCCAAAGGTGTGTGCTCCGTGAG
S. scrofa          ...........AT...................T.T...C...CTT--.A......G......G..C..............
H. amphibius       .C........................G......A.....G...............C........A..........AC...
C. s. simum        ..CT................................C.....................................T.....
T. m. latirostris  ..CT....T...........................C.......................G.............T..T..
L. africana        ..CT....T...........................C.....................................T.....

                           490       500       510       520       530       540       550       560
                   ....|....|....|....|....|....|....|....|....|....|....|....|....|....|....|....|
B. taurus          TCAGCCAGCTATTGACTACCTGCTGAGCCATGGCACTGGCAACCTTGTGGGCATTGTAGTTGGAGGAGTGGGAGAGGCCC
S. scrofa          .........C..C....GT..T..A.A.................................G...................
H. amphibius       .........C..CA..........A.........G..........C..............G.....G.............
C. s. simum        ......G..C..CA.T..............C.A...........................G.....G......C...T..
T. m. latirostris  .........C........................C..........C..............G...........T.......
L. africana        .........C........................C....T.....C..............G...................

                           570       580       590       600       610       620       630       640
                   ....|....|....|....|....|....|....|....|....|....|....|....|....|....|....|....|
B. taurus          TGCAGAGTGTGCCCAACACCACCATGCTCATCCTGCAGAAGCGCAAGGGCTTTGTGCGCATGGCACTCCAGCACGGGGCT
S. scrofa          ...G....................CC.....T..C.......A.....C..CCA---......GTAGGT.T.C.TC....
H. amphibius       ...............G........CC.....T..C................C........CA..C.C...AG.AT.....
C. s. simum        ....A...................CCT.......C..............G..........CA..C........T......
T. m. latirostris  ....A..C................AC........T..............G........A.CA..C...............
L. africana        ....A...................AC........C..............G........A.CA..T........T......
```

```
                                650       660       670       680       690       700       710       720
                       ....|....|....|....|....|....|....|....|....|....|....|....|....|....|....|....|
B. taurus              CATCTGGTCCCCACCTACACTTTTGGAGAAACTGAGGTGTATGATCAGGTGCTGTTCCATAAGGACAGCCGTATGTACAA
S. scrofa              .....A..........T...............C........CA........T................TT..........
H. amphibius           .........T......C.....C...T.....G........CA..........................TA..G......
C. s. simum            ................T.....................C.T...G.................A......A.G.....T..
T. m. latirostris      .....T..T.......T............G..A...........G......T.A....C......T.....G.C......
L. africana            ........T.......T...C........G........A.....G......TCA....C..........T.G........

                                730       740       750       760       770       780       790       800
                       ....|....|....|....|....|....|....|....|....|....|....|....|....|....|....|....|
B. taurus              GTTCCAGAGCTACTTCCGCCAGATTCTTGGTTTCTATTTTTGCATCTTCTATGGACGAGGCTTCCGCCAAGGCTCCACTG
S. scrofa              ........A.......T.......CT..........CC....TG...........T.....................G..
H. amphibius           ...T.......G.......TG...CAC..........C....T.......G....TA........A.....CTC.TG.CA
C. s. simum            .....................T..CT................TG......C...TT.....C..................
T. m. latirostris      ...........GG......GGT..CT................TG..............A..............A...T..
L. africana            ........A..GG......AGT..CT................TG............A.A.....T........A...T..

                                810       820       830       840       850       860       870       880
                       ....|....|....|....|....|....|....|....|....|....|....|....|....|....|....|....|
B. taurus              GGCTCCTGCCGTACCCGCTGCCTATCGTCACTGTGGTGGGGGAGCCTCTGCCTCTGCCCAAAATCGAAAAACCAAGCCAG
S. scrofa              ..........A.........................A.TT......C.................T..G..........T.
H. amphibius           -----------.C.............A.........CGC.A...G...................T...............
C. s. simum            .C........A.....A.........A.......A..T.......T......A...........G.....T.........
T. m. latirostris      .A........A...G.T.G..................T..............A...........T.....G.........
L. africana            ..........A...G.T.G......A...........T..............AG..........T.....G........A

                                890       900       910       920       930       940       950       960
                       ....|....|....|....|....|....|....|....|....|....|....|....|....|....|....|....|
B. taurus              GAGATGGTGGACAAATACCACGCCCTCTACAAGAAGGCCCTGACCAAACTGTTTGATCAGCACAAGACCCAGTATGGCTG
S. scrofa              .....................A....-..T.T.G.C......CA....T.............T...........CA....
H. amphibius           .................G...A.......T.C.G.........A.....CAA.CAGCG----T...........CA....
C. s. simum            .....................A.A.....T.T.G.C......CA............C.....T...........C..T..
T. m. latirostris      .....A.....T...........A.....T.T.C.C...T..CAG..G........C.....T.....T.T.......G.
L. africana            .....A.....T...........A.....T.T.G.C...G..CA............C.....T..A..T...........

                                970       980       990
                       ....|....|....|....|....|....|.
B. taurus              CCCAGAGACCCAAAAGCTGCTTTTCCTGTGA
S. scrofa              .T........................C..A.
H. amphibius           .T.................T...........
C. s. simum            .T.C...T.......................
T. m. latirostris      .T....A..A...G.....G...........
L. africana            .T.......A...G....AG...........
```

Sequence alignment of predicted *Awat2*.

```
                         10        20        30        40        50        60        70        80
                 ....|....|....|....|....|....|....|....|....|....|....|....|....|....|....|....|
B. taurus        ATGGTTTTGCCTTCCAAGAAGGACCTCAAGATCTCCCTGGAGGTCTTTGCTATTTTCCAGTGGGCCCTTAGTGCCTTTGT
S. scrofa        ...C.C.....C..................C.A.G...............G...............CTC.......C.
H. amphibius     ...C...C...C..................CTG.................C...............C........G..
C. s. simum      ...C.CC....C..................CGG.................................C....T...G..
T. m. latirostris ...C.C.....C...........T....A.CTG.....C.AA..........C..............CG.......CC.
L. africana      --------------------------------------------------------------------------------

                         90       100       110       120       130       140       150       160
                 ....|....|....|....|....|....|....|....|....|....|....|....|....|....|....|....|
B. taurus        TATCGTGATCACC--GTGATCGCCGTCAACCTCTACCTGGTGGTGTTCACGCCGTACTGGCCCGTTACCGTGCTCATGCT
S. scrofa        ...T.GA.G....--...C..TTT.............T...........AT.A........T..C..T...G....T..
H. amphibius     ...T..A.G....--......CTT................A.........T.A........T..C..T.......CA..
C. s. simum      ...T.CA.C...T--......CT...........................A........TA.C..T........T..
T. m. latirostris ..CT.CA......AT.....TTT.G...............A.........T.A........AA.C..T........T..
L. africana      -----CA.....TAT....C.TGT.G.......C................AT.A........AA.C..T...T....TT.

                        170       180       190       200       210       220       230       240
                 ....|....|....|....|....|....|....|....|....|....|....|....|....|....|....|....|
B. taurus        TACCTGGCTGGCTTTTGACTGGAAGACCCCTGAGAGAGGTGGCCGCCGGTTTACCTGCGTGAGGAAATGGTGCCTGTGGA
S. scrofa        C.............G..............G...C.....TTT.AGGT.GC.G..G.T.G.CCT.CG..AG..G.A....
H. amphibius     ..................................C..........................C.T.............
C. s. simum      C.................................C....C...........G.....T......C.T...A........
T. m. latirostris C.................................C........T.....C.......T......C.T............
L. africana      C..........................T....C........T...A.C.......T......C.T...CA........

                        250       260       270       280       290       300       310       320
                 ....|....|....|....|....|....|....|....|....|....|....|....|....|....|....|....|
B. taurus        AGCAGTACTGTGACTACTTCCCACTCAAGCTTCTGAAGACTCATGATCTCTCCCCCAGCCTCAACTATATCGTCGCCTGC
S. scrofa        .A.G........T......T..........T.........................AT.A......C...C........
H. amphibius     .A..........T..................T..................................C...C........
C. s. simum      .A......G....T........C........T..............A..........G.A......C...C.T.T....
T. m. latirostris .A.......A...T........T........TG..T..........A....T....A..ATG.G..C...C.T.T....
L. africana      .ATG.........T........T.....T...TG..T.........A....T....A..A......C...C.T.T....

                        330       340       350       360       370       380       390       400
                 ....|....|....|....|....|....|....|....|....|....|....|....|....|....|....|....|
B. taurus        CACCCTCATGGGCTCTTGTCCCATTCATGGTTTGGCCACTTTGCCACAGAGATGTCAGGCTTCTCCAAGACATTTCCTGG
S. scrofa        ...........A...A.......CG...AC.............G.......CC..G.............TC........
H. amphibius     ...............A............C..........................................TC........
C. s. simum      ...............C.A..T......CAC..............A.......CC................TC........
T. m. latirostris T..........A..AA.........C.AC....C................C..CA..............TC........
L. africana      ...........CA..G......T...C.AC.............T.........CA..............TC...T....

                        410       420       430       440       450       460       470       480
                 ....|....|....|....|....|....|....|....|....|....|....|....|....|....|....|....|
B. taurus        CATCACTCCTTATGTCCTCACACTGGGGGCCTTTTTCTGGGTGCCTTTCCTCAGAGAATATATCATGTCTACAGGGGCCT
S. scrofa        ..............G......C.....TA..........C..........C....C.........G..GT....A...
H. amphibius     A...........A.................................G........G............C....
C. s. simum      ......C.......A..........A...............................G........G.....TG..
T. m. latirostris .C....C.TC...A.G..........A...........T................C....CA..A............
L. africana      .C....CTTC...A.G..........A...........T.........C......C....CA..A...G.....CT.A

                        490       500       510       520       530       540       550       560
                 ....|....|....|....|....|....|....|....|....|....|....|....|....|....|....|....|
B. taurus        GCTCTGTGAGCCAATCCTCCATGGACTTCC--TGCTTACCCGTAGAGGCACAGGCAACATGCTGATTGTGGTGGTTGGCG
S. scrofa        ............G.........A.......--.A..C...AAG.....AG.........................C..T.
H. amphibius     ..............................--.........C........................................T.
C. s. simum      ............G.............AT.--.........A..A...........................T.
T. m. latirostris .............T.............T.--.......G.AC.........A....A...T.C.........CC...T.
L. africana      C..GCC...TGTG.GG....T..AT.....CC.C...TGAA.A..TAAGGAG.AA.GTT....C..C......TCA.T-.

                        570       580       590       600       610       620       630       640
                 ....|....|....|....|....|....|....|....|....|....|....|....|....|....|....|....|
B. taurus        GCCTGGCTGAGTGCAAATATAGCCTGCCTGGATCTACCACTCTGTTCCTGAAGGGCCGCACTGGCTTCGTACGCACGGCC
S. scrofa        ...............G...C........A..........C...G..T.....AA...........T.......T....
H. amphibius     ...............G...C........G..........C...G..T.....AA.......C....T.......A...
C. s. simum      ......G........G...C........A..........A...G..T.....AAT..G........T.......T....
T. m. latirostris ......T...A....G...C.....A...A..C.......TC...G..T.A...AAT.AG.G......T..GT...T....
L. africana      .......A.......G...C.A..A...A..C........C...G..T.A...AAT.AG.G..C...T..GTA..T.C..

                        650       660       670       680       690       700       710       720
```

```
                    ....|....|....|....|....|....|....|....|....|....|....|....|....|....|....|....|
B. taurus           CTTCAGCACGGGGTGGCTCTAATCCCAGCGTACTCCTTTGGGGAGACAGAACTCTACAAACAGTACACTTTCACCCCAGG
S. scrofa           ....G...T..CA..TG.........T..C...A.........................T...C...T......T..T..
H. amphibius        ....G...T...........C.....C..C...G.........................T...C...T......T..T..
C. s. simum         ....G...T.................T..C..TG................C........T...C...T....T.T..T..
T. m. latirostris   ....G...T.......G.......T.CAGT..T.........T...TG..C.....TG.T...CTT.T......T..T.A
L. africana         ...TG...T...............T.CAGT..T.T...............CT....TG.T...CCT.T.....AT..T.A

                           730       740       750       760       770       780       790       800
                    ....|....|....|....|....|....|....|....|....|....|....|....|....|....|....|....|
B. taurus           GGGCTTCATCAATCGCTTCCAGAAGTGGTTCCAGAGTATGGTGCACATCTACCCTTGTGCTTTCTATGGGCGTGGCTTCA
S. scrofa           A............A..........C...C...C..CC...A.......................CA............
H. amphibius        .......G...G....................................................................
C. s. simum         ......GG....................C.T...........A...........C.........................
T. m. latirostris   ......TG....CT.......C.GCAT.GCA..T.------------......................A.AC....A..
L. africana         ..CT..GTC..C.-....G.................C....CA..T.......T...........G...A..C.-.....

                           810       820       830       840       850       860       870       880
                    ....|....|....|....|....|....|....|....|....|....|....|....|....|....|....|....|
B. taurus           CTGAGAACTCCCTGGGCTTTCTGCCCTACGCTCAGCCTGTTACCACCATTGTTGGGAAGCCTCTACCACTGCCCAAGATT
S. scrofa           ...........TG....C...............G......A...........G..............C......C...C.
H. amphibius        .....C.....TG....C...............G......A.......................................
C. s. simum         .C.........TG....C..T.......T....G......A...........C...............G.A.........
T. m. latirostris   .CA........AG....C.........GT..C........A.......................................
L. africana         TCAG.......AGA...CC.....................A...........G...G.......................

                           890       900       910       920       930       940       950       960
                    ....|....|....|....|....|....|....|....|....|....|....|....|....|....|....|....|
B. taurus           GAGAACCCAAGCAAGAAGACGGTGGCTAAATACCACGCAATCTATGTGGATGCCCTGCGCCA---ACTGTTTGACCAGCA
S. scrofa           ........G...C..G....A...............A.GC.....A.CA....T....A.A.---.........T.....
H. amphibius        ........G......G....T...........T.....GG.....A.C............A.---...............
C. s. simum         ........G...C..G...TT..................C.....A.T............A.---...............
T. m. latirostris   ............C.......TA...T.........T...C.....A.T......A.A.A.A.TAC...A......T....
L. africana         ............C.......TA.CA..............C.....A.T........A.A.A.---...............

                           970       980       990      1000
                    ....|....|....|....|....|....|....|....|....|....
B. taurus           CAAGACCAAGTTTGGCTTCTCAGAGGCCCAAGAGCTGGTGGTAACTTGA
S. scrofa           ...A.......A.............A....GA........A..GT....
H. amphibius        ..........C..............A....G.........C........
C. s. simum         T...............A........A....G.....A...A........
T. m. latirostris   T.......C.........G......A....G.........A.GGT....
L. africana         T....T..CT......A.G......A....GT........A.GGT....
```
