# Supplementary material for: Complete Inactivation of Sebum-Producing Genes Parallels the Loss of Sebaceous Glands in Cetacea
Source: Mol Biol Evol. 2019 Mar 20;36(6):1270–80. doi: 10.1093/molbev/msz068 (PMC6526905; doi:10.1093/molbev/msz068)

Supplementary Material 4: Analysis of *Hippopotamus amphibius* RNA-seq SRA (SRX1164570) avaliable at NCBI.

H. amphibius	*Dgat2l6*	*Mogat3*	*Awat1*	*Awat2*	*Elovl3*	*Fabp9*	*Scd1*
Mature	156	>330	0	0	12	0	>500
Imature	6	<5	0	0	1	0	<5

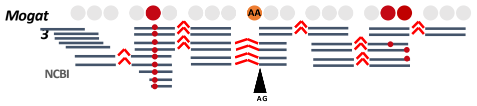

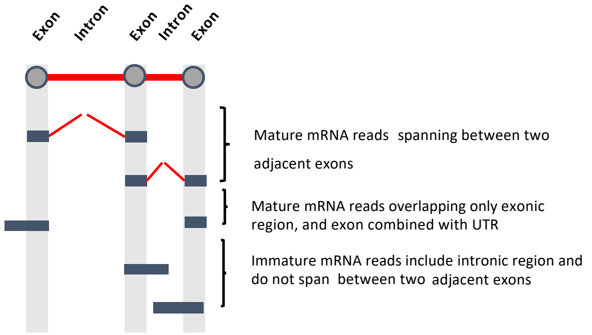

Supplementary Material 4 - *H. amphibius Mogat3* transcriptomic read alignment.

***Hippopotamus amphibius* - *MOGAT3* exon2 Transcriptomic reads**
In-house skin transcriptome

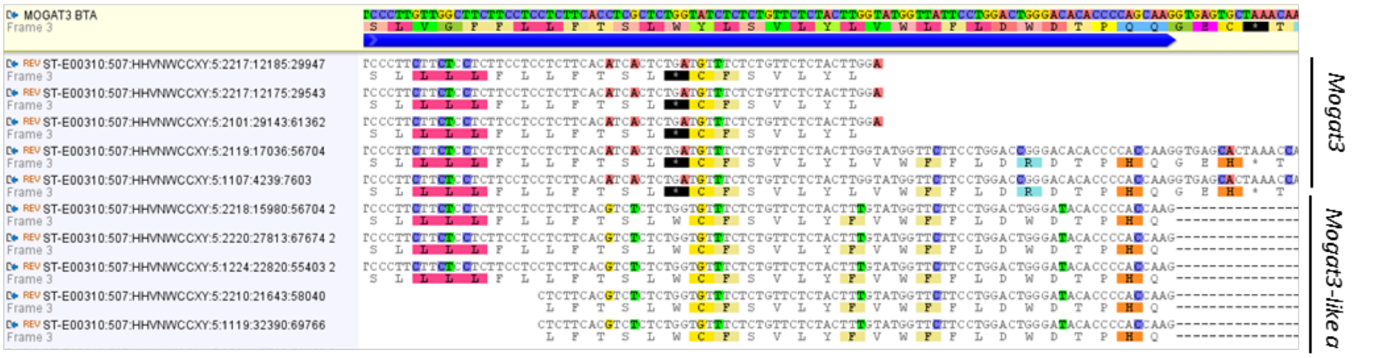

Hippopotamus amphibius* - *MOGAT3* vs *MOGAT3-like
SRA searched
SRR5663647- Senckenberg Gesellschaft fuer Naturforschung 2018-04-05 **Sample ID:** SAMN07206988 (HIP001)
SRR5663648- Senckenberg Gesellschaft fuer Naturforschung 2012-09-20 **Sample ID:** SAMN07206988 (HIP001)

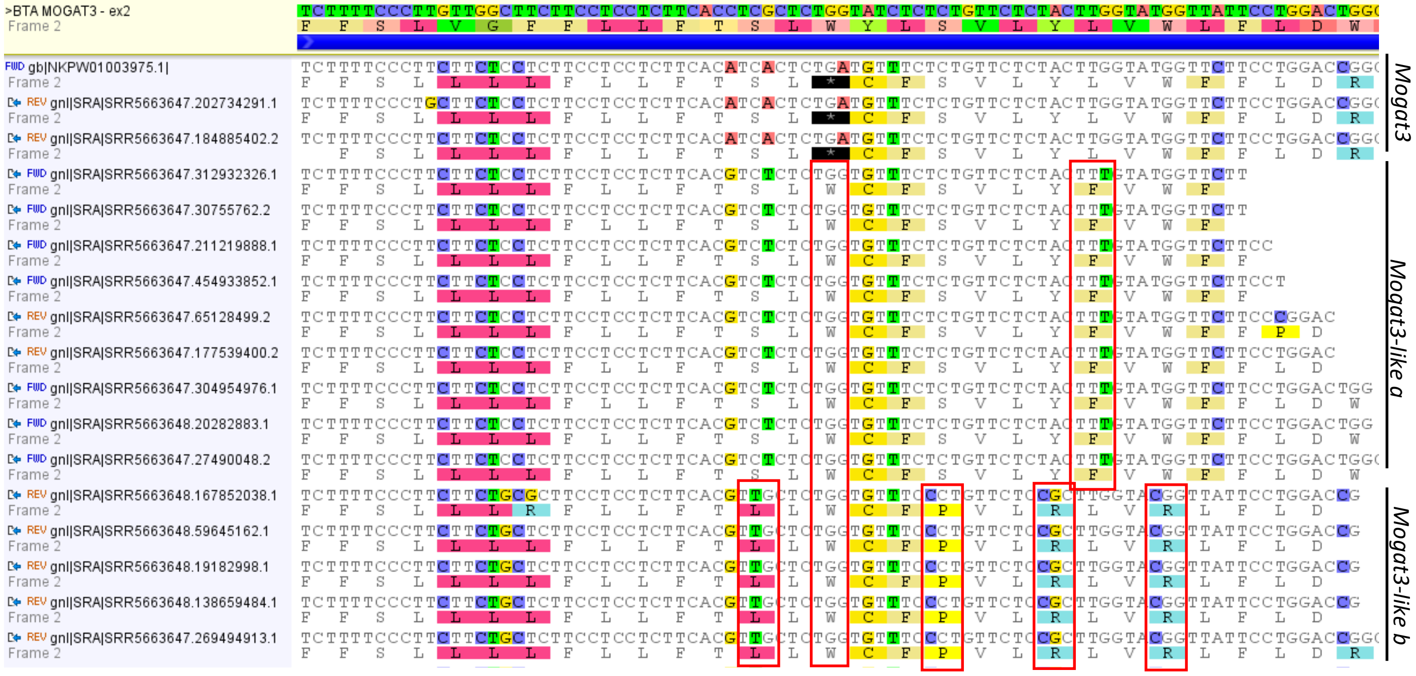

Supplement: msz068_Supplementary_Material [file msz068_supplementary_material.zip › SUPPLEMENTARY_MATERIAL_4.pdf]
