# Supplementary material for: Complete Inactivation of Sebum-Producing Genes Parallels the Loss of Sebaceous Glands in Cetacea
Source: Mol Biol Evol. 2019 Mar 20;36(6):1270–80. doi: 10.1093/molbev/msz068 (PMC6526905; doi:10.1093/molbev/msz068)
Supplement: msz068_Supplementary_Material [file msz068_supplementary_material.zip › SUPPLEMENTARY_MATERIAL_5.pdf]

Supplementary Material 5
Predicted CDS sequences of T. truncatus (TTR) and H. amphibius (HAM)used as query in transcriptomic searches

>TTR_AWAT1
ATGCCTTGTTTCAAGCAACCTAAGCACTTCCAGAGTCTGGTATTTCTGTACTGGCCCCTGAGCGACCTTGCCA
TATTTTGGATCTTGCAACCATTGTCCATTTACCTGCTGTTTACATCTTAGTGGCCGCTACCAGCCCTCTACTT
TGCCTGTTTTTTCCTGGACTGGAAGACCCCAGAGCAAGGTGGCAAGCAATCAGCCTGGGTAAGGAACTGGTGT
GCCTGGACCCGCATCAGGGACTATTTTCCCATTTCT

>TTR_AWAT2
ATGCTCTTGCCCTCCAAGAAGGACCTCAAGACCGCCCTGGAGGTCTTTGCTCTTTTCCAGTGGGCCCTCAGCG
CATTGGTTATCGTAAGCACTGTGATCCTTGTCAATCTCTACCTGGTAGTGTTCACACCATACTGGCCTGTCAC
TGTGCTGCTCACATGGCTGGCTTTTGACTGGAAGATCCCTGAGCGAGGTGGCTGCCGGTTTACCTGCGTAAGG
CACTGGTGCCTGTGGAAACAGTACTGTGATTACTTCCCACTCAAGCTTTTGAAGACTCACGATCTCTCCCCTA
GCCTCAACTACATCCTCGCCTGCCATCCTCATGGGCTCATGTCCCACTCATGCTTTGGCCACTTTGCCACAGA
GAAGTCAGGCTTCTCCAAGATCTTTCCTGGCATCACTCCTTATGTCCTCACACTGGGGGCCTTTTTCTGGGAG
CCTTTCCTCAGAGAATATGTCATGTCTACAGGGGCCTGCTCTGTGAGCCAGTCCTCCATGGACTTCCTGCTTA
CCCGTAGAGGCACAGGCAACATGCTGATCGTGGTGGTCAGTGGCCTGGCTGAGTGCAGATACAGCCTGCCAGG
ATCTGCCACCTTGGTCTTGAAGAACCTCACCGGCTTTGTACGCACGGCCCTTCGGCATGGGGTGGCTCTAATC
CTTGCCTATGCCTTTGGGGAGACAGAACTCTACATAAAGCAGATTTTCACTCCCGGGGGCTTCGTTAATCGCT
TCCAAAAGTGGTTCCAGAGTATGGTACACATCTACCCTTGTGCTTTCTACGGGCATGGCTTCATTGGAACTCC
TGGGGCCTTCTGCCCTACGCTCGGCCTGTAACCACCATTGTTGGGAAGCCTCTACCACTGCCCAAGATTGAGA
ACCCGAGCAAGGAGACGGTGGTAAAATACCATGTGGTCTATATCGATGCCCTGCACAAACTGGCCAGCACAAG
ACCAAGTTTGGCTTCGCAGAGACCCAGGAGCTGGTGCTAACTTGA

>TTR_DGAT2L6
ATGGCCATGGCCTTCCTCTCCCATCTGGATCTTCAGGAAAGCCTCCAAACACTCTCTGTTTTGCAGTGGATCT
CAGTCTATGTCTTTTTAGGAGCTATTCCTATTCTTCTTATACCCTACTTTTTGGTGTTCACTAAGTTCGGCAT
GCTGTCCGTGCTCGCCTTAGCCTGGCTCTCCTATGACGAACACCCACAGTCAAGGTGGTAGGCGTTCAACTTG
GGTACGAAACTGGACCCTCTGGAAGTATTTCCAAATTTCTTCCCAGTAAAGCTGGTGAAGACTCATGACCTCT
GTCCCAAACACAACTATATCATTGCCAACCACCCCCATGGCATTCTCTCTTATGGTGTCTTCATCATCTTTCC
CACTGAGGCCACTGGCTTTGCTCGGATTTTCCCAGCCATCACTCCTTATGTAGGGACCTTGGAAGAGATCTTC
TGGATCCCAATTGTGCGAGATTATGTGATGTCAATGGGTGTGTGCCCAGTGAGTGAGTTGGCCTTGAAGTATT
TGCTGACCAAGAAAGGCTCGGGCAATGCTGTGGTTATTGTGGAGGGTGGATCTGCTGAAGCCCTCTTGTGCCA
CGCAGGAGCCTCTACCGTCCTCCTTAAACAGCATAAAGGTTTTGTGAGGTTGGCACTGAAGACAGGATCATAC
CTTGTTCCCTCCTATTCCTTTGGAGAGAATGAGGTTCACAATCAAGAGACCTTCACTGAGGGCACGTGGCTAA
GGTTCTTCCAAATAAACCTTGCAGGACAGAATCAAAAAAACCCTGAGACTAAGTTTCTGTACCTTCCACGGCC
GGGGCCTCACTCGAGGATCCTGGGGCTTCTTGCCTTTCAATCGGCCCATTACCACTGTTGCTGGGGAGCCCCT
GCCAATTCCCAAGATTAAGAAGCCAAACAAAAAGATGGTGGACAAGTACCACGCACTCTACATCAGGACCCTG
TGCAAGCTGTTTGACAAAGTACAGTATGGCCTCCCTGAGACCCAGGAGCTGACAATCATATAA

>TTR_ELOVL3
ATGGTCACAGCCATGAATGTCTCAGATGAAGTAGAGCAGATGTTCCAGCCCTACAACTTCGAGCTGTTCCAGG
ACATAAGGCCCGTTTTGGAGGAGTACTGGGCAACCTCATTCACCATAGCTCTGATCTACCTGCTGCTCATCTT
TGTGGGGCAGAACTACATGAGGGCACGGAAGGGCTTCAACCTGCAGGGACCTCTCATCCTTTGGTCATTCTGC
CTTGCAATCTTCAGTATCCTTGGGGCAGTGAGGACATGGGGCTATACGGGGACTGTGCTACTTAGGGGAGCCT
AAAGAAAACTGTATGCTTCTTCATCTTCACCGATAATCCCATAATCAAATTCTGATCCTGCCACTTTGTTCTC
AGCAAGATCATTGAACTTGGAGACACAGCCTTCATCATCCTGTATAAATGGCCACTCATCTTTGTGCACTGGT
ACCACCACAGCACAGAGCTAGTGTACAAGAGCTTTGGATACAAGAACAAGGTGGCTGCAGGCGGCTGGTTCAT
GACCATGAACTACGGTGTATGTGCCATCATGTACACCTACTATACTCTGAAGGCTGCCAAAGTAAAGCCCCCC
AGGTGGTTTCCCATGCTCGTCACCAGCCTGCAAATCCTGCAGATGTTTATGGGAGCTACTGTCGGTATCCTGA
CTTACATCTGGAGACAGGAACAGGGATGCCATACAACAAAGGAACACCTCTTCTGGTCCTTCATCTTGTATAC
AACCTATTTCCACCAAAACTATGTAATTACCAAGGTCAAGGCCAAGACCAAGAGCCAGTGA

>TTR_FABP9
ATGACTGAGCCCTTCTTGGGAACCTGGAAACTGGTCTCCAGTGAAAACTTTGATGAATACATGAAACAACTGG
GAGTGAGTGCTGCAGTCCAGAACCTTGCTGGGTTGACAAAGCCAAGAATCACTATTAGCGCCGACAGGAATAA
GGTTAACAATAAAACAGAAAGTTCTTTCAAAAATACTGAGATCGTGCTTCCCTGGTGGCGCAGTGGTTGGGAG
TCCGCCTGCCGATGCAGGGGACACGGGTAGAATCATAACATTAGATGGTGGCTCAATGATTCATTTCCAAAAA
TGGCTTGGCAAAGAGACAACAATCAAAAGACAAATTGTAGATGGAAAAATGGTAGTGGAATATATCATGAATA
ATATTGTCAGCATTGGAATCTACAAAAAGGTATGA

>HAM_AWAT1
ATGCCTTGTTTCAAGCAGCCTAAGCACTTCCAGAGTCTGGTACTTCTGCACTGGCCCCGAGCTACCTTGTCAT
ATTTTGGATCTTGCAGCCACTGTCCATTTATTTACCTGCTGTTTACATCCTGCTGGCCACCAGCAGCCCTTTA
CTTCGCCTGGTTTTTCCTGGACTGGAAGACCCCAGAGCAAGATCCTGAACGCTAAAGACCTGTCACCTGAGCA
CAACTATCTCATGGGGGTCACCCCCATGGCCTCTTGACATTTGGTGCTTTCTGCAACTCCTGCACTGAGGCCA
CAGGCTTCTCCATCTTCCAGGCATCGCTCCTCCTCTGGCCACGCTGTCCTGGTTCTTGAAGATCACCTTTGTT
AGGGACTACCTCACGGCCAAAGATGTGTGCTCCACGAGTCAGCCAGCCATCAACTACCTGCTAAGCCATGGCG
CTGGCAACCTCGTGGGCATTGTAGTGGGAGGGGTGGGAGAGGCCCTGCAGAGTGTGCCCAGCACCACCACCCT
CATTCTCCAGAAGCGCAAGGGCTCTGTGCGCACAGCCCCCCAAGAATGGGCTCATCTGGTCTCCACCTCCACT
TCTGGTGAAACGGAGGTGTACAATCAGGTGCTGTTCCATAAGGACAGCTATAGGTACAAGTTTCAGAGCTGCT
TCCGCTGGATCACTGGTTTCTATCTTTGTATCTTCTGTGGATAAGGCTTCCACCAAGCTCCTGCCATCCCCGC
TGCCTATCATCACTGTGCGCGGAGAGGCTCTGCCTCTGCCCAAAATTGAAAAACCAAGCCAGGAGATGGTGGA
CAAATAGCACACCCTCTATACGGAGGCCCTGAACAAACCAATCAGCGTAAGACCCAGTACAGCTGCTCAGAGA
CCCAAAAGCTGTTTTTCCTGTGA

>HAM_AWAT2
ATGCTTTCGCCCTCCAAGAAGGACCTCAAGACTGCCCTGGAGGTCTTTGCTCTTTTCCAGTGGGCCCTCAGTG
CCTTGGTTATTGTAAGCACCGTGATCCTTGTCAACCTCTACCTGGTAGTGTTCACGTCATACTGGCCTGTCAC
TGTGCTCACACTTACCTGGCTGGCTTTTGACTGGAAGACCCCTGAGCGAGCTTTTGAAGACTCATGATCTCTC
CCCCAGCCTCAACTACATCCTCGCCTGCCACCCTCATGGGCTCATGTCCCATTCATGCTTTGGCCACTTTGCC
ACAGAGATGTCAGGCTTCTCCAAGATCTTTCCTGGAATCACTCCTTATATCCTCACACTGGGGGCCTTTTTCT
GGGTGCCTTTCCTGAGAGAATATGTCATGTCTACAGGCGCCTGCTCTGTGAGCCAATCCTCCATGGACTTCCT
GCTTACCCGCAGAGGCACAGGCAACATGCTGATTGTGGTGGTTGGTGGCCTGGCTGAGTGCAGATACAGCCTG
CCGGGATCTACCACCCTGGTCTTGAAGAACCGCACCGGCTTTGTACGCACAGCCCTTCGGCATGGGGTGGCTC
TCATCCCCGCCTACGCCTTTGGGGAGACAGAACTCTACAATCAGCACATTTTCACTCCTGGGGGCTTCGTCAG
TCGCTTCCAGAAGTGGTTCCAGAGTATGGTGCACATCTACCCTTGTGCTTTCTATGGGCGTGGCTTCACTGAG
CACTCCTGGGGCCTTCTGCCCTACGCTCGGCCTGTAACCACCATTGTTGGGAAGCCTCTACCACTGCCCAAGA
TTGAGAACCCGAGCAAGGAGACTGTGGCTAAATATCACGCGGTCTATATCGATGCCCTGCGCAAACTGTTTGA
CCAGCACAAGACCAAGCTTGGCTTCTCAGAGACCCAGGAGCTGGTGCTAACTTGA

>HAM_DGAT2L6
ATGGCCATGGGCTTCTTCTCCCGGCTGGATCTTCAGGAAACCCTCCAAACCCTCTCTGTTTTGCAGTGGGTCC
CCGTCTATGTCTTTTTAGGAGCTATTCCTATTCTTCTTATACCCTACTTTCTGGTGTTCACTAAATTCTGGAT
AGTATCCATGCTTGCCTTAGCCTGGCTCGCCTATGACTGGAACACCCACAGTCAAGCTGGTGAAGACTCATGA
CCTCTGTCCCAAACACAACTATATCATTGCCAGCCACCCCCATGGCATTCTCTCTTATGGTGTCTTCGTCAAC
TTTGCCACTGAGGCCACTGGCTTTGCTCGGATTTTCCCAGCCATCACTCCTTATGTAGGGACCCTGGAAGGGA
TCTTCTGGATCCCAATTGTGCGAGATTATGTGATGTCAATGGGTGTGTGCCCAGTGAGTGAGCTGGCCTTGAA
GTATTTGCTGACCAAGAAAGGTTCAGGCAATGCTGTGGTTATCGTGGTGGGTGGAGCTGCTGAAGCCCTCTTA
TGCCACCCAGGAGCCTCTACCATCCTCCTTAAACAGCGTAAAGGTTTTGTGAAGTTGGCACTGAAGACAGGAT
CATACCTTGTCCCTTCCTATTCCTTTGGAGAGAATGAGGTTCACAAACAACCAACCTTCCCTGAGGGCACATG
GCTAAGGTTCTTCCAAAAAACCTTGCAGGACACAATCAAAAAAACCCTGAGGCTAAGTTTCTGTACCTTCTAT
GGCCGGGGCCTCACTCGAGGATCCTGGGGCTTCCTGCCTTTCAATAGGCCCATTACCACGGTTGTTGGGGAAC
CCCTGCCAATTCCCAAAATTAAGAAGCCAAACAAGAAGACGGTGGACAAGTACCACACACTCTACGTCAGTGC
CCTGCGCAAGCTGTTTGACCAGCACAAAGTTCAGTATGGCCTCCCTGAGACCCAGGAGCTGACAATCATATAA

>HAM_MOGAT3
ATGGAAACCCTGCAGAAAGAGTGGCTAGAAGTACTGAGCACCTACTACTACGTGCTCACTTTCGTCTTCATGG
CCCCTTTCTTTTCCCTTCTTCTCCTCTTCCTCCTCTTCACATCACTCTGATGTTTCTCTGTTCTCTACTTGGT
ATGGTTCTTCCTGGACCGGGACACACCCCACCAAGCTGGTGAAAACAGTCGAGCTGCCCCCCAACCGGAACTA
CGTGCTGGTCTCTCACCCACATGGAATCATAAACTTGGGGAGCAACATGAACTTCGCCACTGAGGACACTGGA
TTCTCCCAGAAGTTCTCTGGGATTTGGCCCTTTCCAACAGGGCTGAATGGCATCTTCTACCTCCCAGGCTATC
GAGAGCAAGTTCTATCCCTTGGACTGTGTTCCGTGAGCCGCCGGAGCCTGGATTTTATTCTATCGCGGCCCCA
GCTCGGGCAGGCTATGGTCATCACAGCTGGGGGGGCCCACAAGTCCCTGGACGCCATCCCAGGGGAGCACTGC
CTCATTCTCTGGAACTGGAAAGGTTTTGTCCACTTGGCACTGAGAGATGGCGCCTCTCTGGTGCCCGTGTACT
CCTTCGGGGAGAATGATGTCTTCAGAGTTAAGGCTTTTGGCCCAGACTCGTGGCAGCATCTGTGTCAAGTCAC
CTTCAAGAAGCTCCTGGGCTTTGCTCCTTGCATCTCCTGGGGCCGTGGGCTCTTCTCAGCCAACTCCTAGGGC
CTGGTGCCCTTTGCCAGGCCCATCACCACTGTGGTGGGCCGCCCCATCCCGGTCCCCCAGTGCCTGTGGCCCA
CCGAGGAGCAGGTTGACCACTATCACATGCTCTACATGAAGGCTCTGGAGGAACTCTTTGAGGAGCACAAGGA
AAGCTGTGGTGTCCCAGCTTCTACTCGCCTCACCTTCATGTAG

>HAM_ELOVL3
ATGGTCACAGCCATGAATGTGTCAGATGAAATAGAGCAGATGTTCCAGCCCTACAACTTCGAGCTCTTCCAGG
ACATAAGGCCCTTTTTGGAGGAGTACTGGGCAACCTCATTCCCCATAGCTCTGATCTACCTGCTGCTCATCTT
TGTGGGGCAGAACTACATGAAGTCACGGAAGGGCTTCAACCTGCAGGGGCCTCTCATCCTTTGGTCCTTCTGC
CTTGCAATCTTCAGTATCCTCGGGGCAGTGAGGACGTGGGGCTATATGGGGACCGTGCTACTTATGGGGAGCC
TAAAGCAATCTGTATGCTTCTCCATCTTCGTCGACAGTCCCATAATCAAATTCTGGTCCTGCCTCTTTGTTCT
CAGCAAGATCATTGAACTTGGAGACACGGCCTTCATCATCCTGCGTAAGCGGCCACTCATCTTTATGCACTGG
TACCACCACAGCACAGTGCTAGTGTACACAAGCTTTGGATACAAGAACAAGGTGGCTGCAGGCGGCTGGTTCA
TGACCACGAACTACGGTGTACATGCCATCATGTACACCTACTACACTCTGAAGGCTGCCAAAGTGAAGCCCCC
CAGGTGGTTTCCCGTGCTCATCACCAGCCTGCAGATCCTGCAGATGTGTATTGGAGCCACTGTTGGTATCCTG
ACTTACATCTGGAGACAGGAACAGGGATGCCACACCACAAAGGAACAAGTCTTCTGGTCCTTCATCTTGTATA
CAACCTATTTCCTCCTCTTTGCCCAGTTCTTCCACCAAAACTATATGATTCCCAAGGTCAAAGCCAAGATCAA
GAGCCAGTGA

>HAM_FABP9
ATGATTGAGCCCTTCTTGGGAACCTGGAAGATGGTCTCCAGTGAAAACTTTGATGAATACATGAAACAATTGG
GAGTGAGTGTTGCAGTCCAGAACCTTGCAGGGTTGGCAAAGCCACAAATCACTATTAGTACCAACAGGGATAA
GGTTAACATCAAAACAGAAAGTTCTTTCAAGAACACTGAGATCTCCTTCAAGCTGGGGGAAGAATTTGATGAA
ACCACAGTAGATAACTGGAAAGTGAAGAGCATCATAACATTAGATGGTGGCTCAATGATTCATGTCCAAAAAT
GGCTTGACAAAGAGACAACAATTAAAAGAAAAATTATAGATGGAAAAATGGTAGTGGAATATATTATGAACAA
TATTGTCAGCACTGGAATCTATGAAAAGATATGA
